# Traditional Chinese medicine for non-alcoholic fatty liver disease: an overview of systematic reviews with evidence mapping and metabolic outcome assessment

**DOI:** 10.3389/fphar.2025.1675793

**Published:** 2025-12-10

**Authors:** Juanjuan Li, Ruimin Jiao, Wenquan Su, Wei Chen, Xiangyu Hu, Shuai Xu, Lie Xu, Weiwei Yao, Kejia Liu, Hong You, Jingjie Zhao

**Affiliations:** 1 Department of Traditional Chinese Medicine, Beijing Friendship Hospital, Capital Medical University, Beijing, China; 2 Department of Acupuncture, Beijing Dongzhimen Hospital, Beijing University of Chinese Medicine, Beijing, China; 3 Institute of Basic Research in Clinical Medicine, China Academy of Chinese Medical Sciences, Beijing, China; 4 Liver Research Center, National Clinical Research Center of Digestive Diseases, Beijing Friendship Hospital, Capital Medical University, Beijing, China; 5 Clinical Center for Metabolic Associated Fatty Liver Disease, Capital Medical University, Beijing, China

**Keywords:** non-alcoholic fatty liver disease, traditional Chinese medicine, systematic review, meta-analysis, overview

## Abstract

The paradigm shift from non-alcoholic fatty liver disease (NAFLD) to metabolic dysfunction-associated fatty liver disease (MAFLD) emphasizes metabolic pathogenesis, yet the efficacy of Traditional Chinese Medicine (TCM) under this framework remains unevaluated. Prior reviews focused on NAFLD with outdated data (<2020), lacking clinical translation tools and methodological standards for TCM systematic reviews and meta-analyses (SRs/MAs). This overview integrates NAFLD criteria, visualizes TCM efficacy via evidence mapping, proposes a methodological framework to standardize TCM SRs/MAs, and focuses on evaluating metabolism-related indicators. Nine databases were searched (from database inception to December 30, 2024) for TCM SRs/MAs in NAFLD. Methodological quality was assessed via AMSTAR-2, PRISMA/PRISMA-CHM, and GRADE. Evidence mapping visualized outcomes (liver enzymes, metabolism) to identify clinical priorities. Standardized reporting guidelines for TCM preparations were adhered to, and a ConPhYMP tool assessment evaluated botanical drugs composition and processing disclosure. Thirty-seven SRs/MAs (35 low/critically low quality) reported trends of reduced ALT (−8.2 U/L, 95% CI: −10.1 to −6.3), improved metabolic parameters (e.g., TG: −0.5 mmol/L), and enhanced B-ultrasound resolution (RR: 1.62), though these findings are limited by methodological flaws and low-quality evidence. Evidence mapping highlighted Xiaoyao Powder and Danning Tablet as top-performing formulas. A methodological framework addressing TCM heterogeneity (formula standardization, dosage) and reporting biases was proposed. This is the first overview integrating NAFLD criteria to visualize TCM-related evidence, offering preliminary observations on potential associations between TCM interventions and metabolic outcomes in NAFLD (interpreted with caution due to low evidence quality). The evidence map and methodological guidelines provide a foundation for standardized future TCM research, while clinical translation of current findings is not recommended due to insufficient high-quality evidence.

## 1 Introduction

Non-alcoholic fatty liver disease (NAFLD) is presently recognized as the most prevalent chronic liver disease in the world (Gofton et al., 2023; Han et al., 2023). Recent academic discourse has led to the proposal of a nomenclature update from NAFLD to metabolic dysfunction–associated fatty liver disease (MAFLD) or Metabolic dysfunction-associated steatotic liver disease (MASLD) (Hsu and Loomba, 2024). In China, experts contend that there is a significant overlap in the essence of MAFLD and MASLD (Fan et al., 2024), and that the two terms can be adopted concurrently. This study adopts the term NAFLD to align with primary literature, while acknowledging its relevance to MAFLD/MASLD metabolic mechanisms. Beyond metabolic dysfunction, MAFLD introduces more explicit diagnostic criteria, integrating metabolic syndrome features (e.g., obesity, insulin resistance, dyslipidemia) to enable precise patient stratification. Furthermore, to maintain consistency with the original literature, this paper continues to use the NAFLD nomenclature and focuses on the analysis of metabolism-related indicators.

Consequently, accumulated evidence from NAFLD-related research remains clinically relevant and applicable for ongoing scientific investigations under the new nomenclature. NAFLD affects approximately 30% of adults globally, with metabolic dysfunction as a key driver (Younossi et al., 2023). Current therapeutic approaches for the disease emphasize increased physical activity (Smart et al., 2018), dietary modification (Tsompanaki et al., 2023), the use of liver-protective (Eslam et al., 2020) and lipid-lowering (Rinella et al., 2023) pharmacological agents, and bariatric surgery (Rinella et al., 2023; Fernando and Pedro, 2021). Uncertainty about the clinical efficacy of these approaches and the potential for adverse event occurrence limits their clinical application (Rinella et al., 2023; Cusi et al., 2022). Persistent uncertainty regarding the clinical efficacy of current NAFLD/MAFLD/MASLD therapies is partly attributable to the condition’s profound heterogeneity in progression. The disease exhibits a broad spectrum of clinical outcomes, ranging from indolent, asymptomatic disease to the development of nonalcoholic steatohepatitis (NASH), fibrosis, and cirrhosis. A major limitation of conventional pharmacotherapy is its inconsistent ability to halt disease progression in advanced stages, yielding dichotomous responses among different patient subgroups (Kumar et al., 2021). This variability underscores the rationale for exploring alternative modalities, including TCM, which potentially targets unique metabolic and hepatic pathways. A critical imperative exists to evaluate such complementary and alternative medical approaches within the NAFLD management paradigm.

TCM has demonstrated significant efficacy in the treatment of NAFLD (Chen et al., 2021; Yang et al., 2019), primarily via the enhancement of liver function, glucose regulation, lipid metabolism and the promotion of weight loss (Kim et al., 2024; Peng et al., 2022; Zhang L. et al., 2022; Zhang Y. F. et al., 2022). Numerous SRs/MAs have indicated that TCM confers certain benefits in the treatment of the disease. One similiar overview of SRs/MAs (Dai L. et al., 2021) synthesized TCM efficacy for NAFLD but reported results: for ultrasound-visualized improvements, 4/7 included SRs showed significant benefits (e.g., liver/spleen CT ratio reduction, RR = 1.28, 95% CI: 1.05–1.56), while 3/7 found no significant differences (RR = 1.06, 95% CI: 0.90–1.25); for ALT normalization, effect sizes were small and heterogeneous (pooled MD = −3.2 U/L, 95% CI: −6.5 to 0.1; I^2^ = 68%), with 5/8 SRs/MAs showing non-significant trends. These inconsistencies-attributed to variable TCM formulations (e.g., Xiaoyao Powder vs. Danning Tablet) and small sample sizes (median n = 286 per SR)-highlight the need for updated synthesis incorporating 2020–2024 data to resolve discrepancies. To enhance the reliability and validity of findings, we updated the data and comprehensively evaluated the scientific quality of SRs/MAs of TCM for NAFLD. We identified methodological limitations of existing SRs to guide future research in this area. The results can inform clinicians, patients with NAFLD, and policymakers.

## 2 Methods

### 2.1 Protocol and registration

The protocol for this overview was registered in the PROSPERO database (CRD42024554175) on 10 June 2024.

### 2.2 Search strategy

Nine electronic databases (PubMed, The Cochrane Library, Web of Science, the China National Knowledge Infrastructure, the China Biology Medicine disc, Wanfang Data, the VIP Database for Chinese Technical Periodicals, the Excerpta Medica Database, and the Scopus abstract database) were searched for relevant SRs/MAs published in English or Chinese from database inception to December 30, 2024, to ensure inclusion of the most recent evidence. Clinical trial registries (the World Health Organization’s International Clinical Trials Registry Platform, Clinical Trials, and the Chinese Clinical Trial Registry) and the Allied and Complementary Medicine Database were also searched. The search strategy involved the integrated use of subject headings with free text terms. The key terms included “nonalcoholic fatty liver disease,” “Chinese medicine,” and “systematic review.” The detailed search strategy used for each database is shown in Supplementary Table S1.

### 2.3 Inclusion and exclusion criteria

SRs/MAs covering the use of TCM to treat NAFLD that were published in peer-reviewed journals were eligible for study inclusion. Eligible studies involved participants of any sex, age, and race who were diagnosed with NAFLD (Eslam et al., 2020), treatment with TCM alone or in combination with Biomedicine (Biomed), and comparison with Biomed treatment or placebo. TCM interventions, defined as standardized TCM formulas complying with the Chinese Pharmacopoeia (excluding single-botanical drug extracts and injectables. Non-pharmacological TCM interventions (e.g., acupuncture) were excluded to focus on botanical drug efficacy. These modalities differ in mechanism (physical stimulation vs. pharmacological effects) and standardization (practitioner-dependent vs. formula-based), preventing conflation of results and ensuring conclusions specifically reflect botanical drug efficacy. Eligible studies must report at least one of these outcomes, including clinical total effective rate, laboratory test parameters related to liver function [alanine aminotransferase (ALT)/aspartate aminotransferase (AST) for hepatocellular injury, gamma-glutamyl transferase (GGT) for biliary dysfunction, total bilirubin (TBil) for cholestasis], laboratory test parameters related to glucose and lipid metabolism [total cholesterol (TC)/triglyceride (TG) for dyslipidemia, fasting blood glucose (FBG)/homeostatic model assessment for insulin resistance (HOMA-IR) for insulin resistance, glycated hemoglobin (HbA1c) for long-term glycemic control], imaging examination-based parameters [liver/spleen computed tomography (CT) ratio and B-ultrasound for steatosis quantification, liver stiffness for fibrosis], anthropometric parameters related to metabolism [body mass index (BMI) for adiposity], and safety outcomes (adverse events).

### 2.4 Reporting standards for TCM preparations

All botanical ingredients in the TCM formulations included in this study underwent rigorous taxonomic validation to ensure scientific accuracy and reproducibility. The validation was performed using two authoritative taxonomic databases: Kew Plants of the World Online (http://www.plantsoftheworldonline.org) — a globally recognized resource for plant taxonomic information, and Flora of China, which provides region-specific taxonomic confirmation for Chinese medicinal plants. For each botanical ingredient, the complete taxonomic information was verified and standardized, including the accepted scientific name (genus + species), authority (naming author), and family classification. Ingredients with initially incomplete taxonomic records (e.g., undefined family or missing authority, such as Polygonum cuspidatum and *Lonicera japonica*) were supplemented through cross-referencing with taxonomic monographs, peer-reviewed literature, and official pharmacopoeial records. For example, Polygonum cuspidatum was validated as Polygonum cuspidatum Sieb. & Zucc. (Polygonaceae), and *Lonicera japonica* was confirmed as *Lonicera japonica* Thunb. (Caprifoliaceae), consistent with the latest taxonomic revisions.

All botanical ingredients are presented in a unified standard format: Chinese common name (validated Latin name + authority) [family; pharmacopoeial drug name]. A representative example is Lamiaceae (Salvia miltiorrhiza Bunge)[Lamiaceae; Salviae Miltiorrhizae Radix et Rhizoma]. The pharmacopoeial drug names and corresponding quality standards were adopted from the Chinese Pharmacopoeia (2020 Edition, Volume Ⅰ), with specific volume and page references provided for each ingredient to facilitate quality verification (see Supplementary Table S7).

### 2.5 Study selection

After the removal of duplicate publications using EndNote X 9.1 (Clarivate Analytics, Philadelphia, PA, USA), the titles and abstracts of all potentially eligible publications were screened and irrelevant studies were excluded. Then, the full texts of the remaining studies were read to compile the final study sample. Two reviewers (Shuai Xu and Wei Chen) independently reviewed all studies. Any disagreement was resolved by a third reviewer (Hong You or Jingjie Zhao). Inter-rater agreement quantified using Cohen’s kappa (κ) coefficients:κ = 0.82 (95% CI: 0.71–0.93), indicating substantial agreement.

### 2.6 Data extraction

Two reviewers (Lie Xu and Kejia Liu) independently extracted the following data from the included studies using a standardized form: basic information (title and first author name, year of publication, number of included trials, total sample size, intervention and control treatments, outcome measures, effect measures for the outcomes of this overview), methodological quality, reporting quality, evidence quality, and adverse events. A third reviewer (Hong You or Jingjie Zhao) checked the accuracy of the data and resolved any inconsistencies through discussion with the two reviewers. Inter-rater agreement quantified using Cohen’s kappa (κ) coefficients: κ = 0.78 (95% CI: 0.65–0.91), indicating substantial agreement.

### 2.7 Assessment of methodological quality

The Assessing the Methodological Quality of Systematic Reviews 2 (AMSTAR-2) tool (Shea et al., 2017) was used to assess the methodological quality of the included SRs/MAs. This tool has shown good inter-rater agreement, test-retest reliability, and face and construct validity (Shea et al., 2017). It consists of 16 items, with the response options of “yes,” “partial yes,” and “no.” Seven of the items (2, 4, 7, 9, 11, 13, and 15) are critical for SR evaluations. According to the AMSTAR-2 guidelines, the overall confidence in each evaluated SR was classified as “high,” “moderate,” “low,” or “critically low.” Two reviewers (Xiangyu Hu and Weiwei Yao) independently assessed all studies. Any disagreement was resolved by a third reviewer (Hong You or Jingjie Zhao).

### 2.8 Assessment of reporting quality

The Preferred Reporting Items for Systemic Reviews and Meta-Analyses (PRISMA) 2020 (Page et al., 2021) and PRISMA Extension for Chinese Herbal Medicines (CHM) (Zhang X. et al., 2020) statements were used to assess the quality of reporting on interventions in the SRs/MAs. The PRISMA 2020 statement consists of 27 items in 7 modules, and the PRISMA-CHM statement consists of 27 extension items and subitems. All items are graded as “completely reported” (“yes”; 1 point), “partially reported” (“partial yes”; 0.5 points), or unreported (“no”; 0 points), with a maximum possible total score for each instrument of 27 points. Scores <16 indicate critical information failure, scores of 16–21 reflect certain information deficits, and scores >21 indicate relatively complete reporting. Two reviewers (Juanjuan Li and Ruimin Jiao) independently assessed all studies. Any disagreement was resolved by a third reviewer (Hong You or Jingjie Zhao).

### 2.9 Assessment of evidence quality

The Grading of Recommendations Assessment, Development, and Evaluation (GRADE) tool (Balshem et al., 2011) was used to evaluate the quality of evidence for each of our outcome measures. Evidence quality was assessed using GRADE, with downgrading for: (1) risk of bias, (2) inconsistency, (3) imprecision, (4) indirectness, and (5) publication bias. No downgrading of any criterion reflects high-level evidence, the downgrading of one criterion indicates moderate-level evidence, the downgrading of two criteria indicates low-level evidence, and the downgrading of three or more criteria reflects very low-level evidence. Two reviewers (Wei Chen and Xiangyu Hu) independently assessed all studies. Any disagreement was resolved by a third reviewer (Hong You or Jingjie Zhao).

### 2.10 Compilation and graphical visualization of results

The basic information, clinical characteristics, and quality metrics for all included SRs/MAs were descriptively synthesized and summarized. In addition, bubble plots based on the GRADE (Balshem et al., 2011) results were created. The plots depict the outcomes (*x* axis), *P* values for the overall TCM treatment effects (*y* axis), number of randomized controlled trials (RCTs) included (bubble size), and SR/MA quality according to GRADE assessment (color).

### 2.11 Evaluation of inter-rater agreement

Agreement between reviewers for each variable was evaluated by calculating Cohen’s *κ* values using SPSS (version 20; IBM Corporation, Armonk, NY, USA). It was characterized as good (*κ* > 0.8), substantial (0.6 < *κ* ≤ 0.8), moderate (0.4 < *κ* ≤ 0.6), fair (0.2 < *κ* ≤ 0.4), or poor (*κ* ≤ 0.2). The agreement of AMSTAR 2 between the two researchers was judged strong or relatively strong (κ > 0.60). The agreement of PRISMA2020 and PRISMA-CHM between the two researchers were judged strong or relatively strong (κ > 0.60). Inter-rater agreement for AMSTAR-2 assessments was substantial to almost perfect (κ > 0.60), indicating strong consistency between reviewers. Similarly, substantial agreement was observed for PRISMA 2020 and PRISMA-CHM evaluations (κ > 0.60), confirming reliable application of reporting quality criteria across reviewers.

## 3 Results

### 3.1 Study selection

**FIGURE 1:**
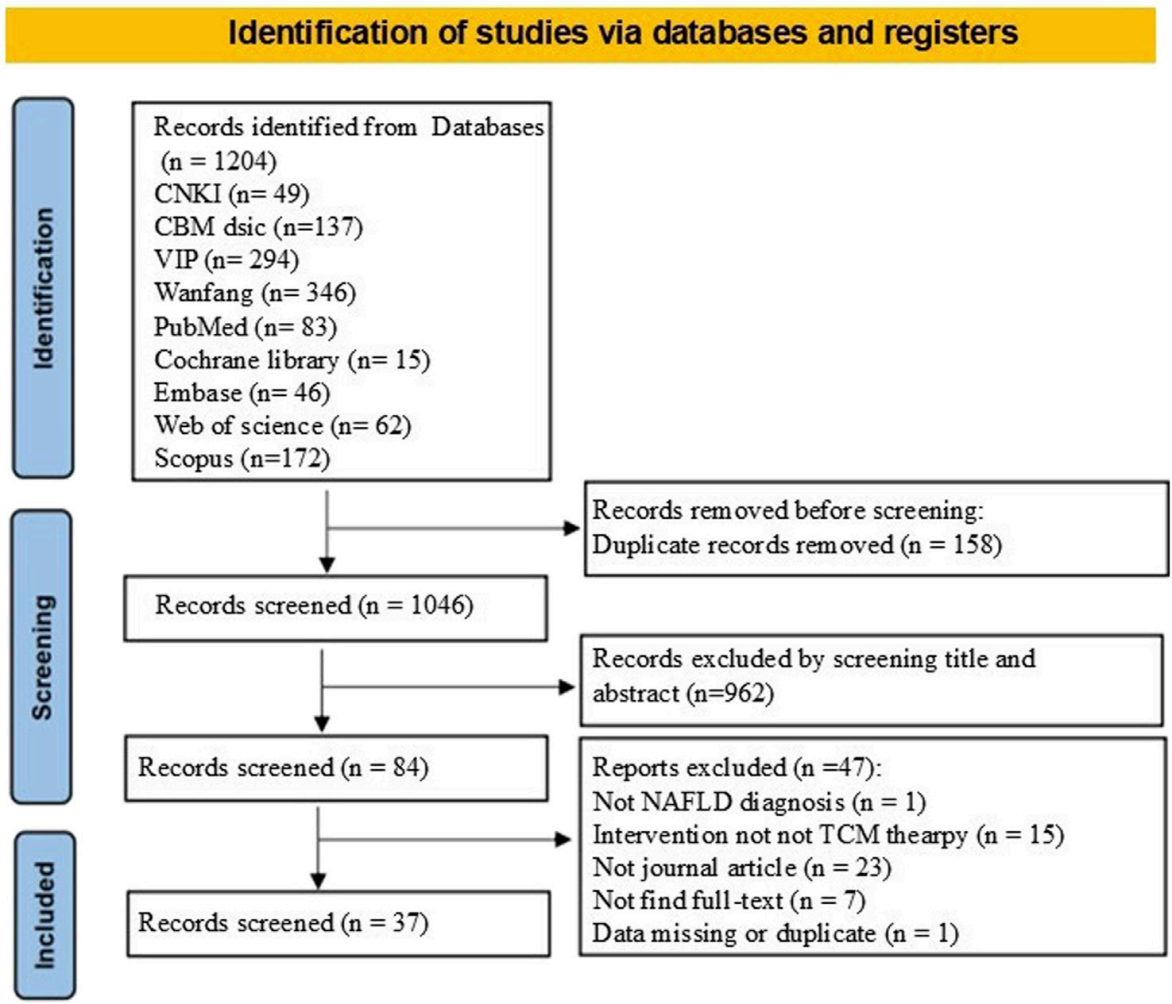
PRISMA diagram of study selection process.

In total, 1,204 relevant studies were identified across all nine databases. Following the application of the inclusion and exclusion criteria, the full texts of 84 studies were screened. Ultimately, 37 SRs/MAs were included in this overview (Figure 1).

### 3.2 Study characteristics

The characteristics of the SRs/MAs are summarized in Table 1. All of the studies were published between 2010 and 2024. Ten were published in English (Cai et al., 2019; Dulmini et al., 2024; Kim et al., 2024; Liu et al., 2022; Peng et al., 2016; Peng et al., 2022; Shi et al., 2012; Wang et al., 2021; Zhang L. et al., 2022; Zhang X. et al., 2024), and 27 were published in Chinese (Ding et al., 2021; Dong et al., 2023; Gao and Xue, 2020; Gong et al., 2014; Han et al., 2024; He and Jiang, 2010; Huang et al., 2017; Li et al., 2011; Liu et al., 2023; Ma et al., 2018; Mou et al., 2017; Qi et al., 2015; Qin et al., 2022; Shen and Gong, 2024; Shi et al., 2022; Wei and Ji, 2012; Wu et al., 2018; Wu et al., 2017; Xie et al., 2018; Yang and G, 2019; Yi et al., 2018; Zhang and Zhang, 2014; Zhang et al., 2014; Zhang P. P. et al., 2024; Zhang X. W. et al., 2020; Zhao et al., 2022; Zhou and Gao, 2018). Sample sizes ranged from 4 to 62, and the numbers of participants ranged from 500 to 13,741. Four of the SRs/Mas (Peng et al., 2022; Xie et al., 2018; Zhang P. P. et al., 2024; Zhou and Gao, 2018) focused on populations with NAFLD and diabetes mellitus. Interventions consisting of TCM alone were examined in 17 SRs/MAs (Cai et al., 2019; Dong et al., 2023; Gao and Xue, 2020; Gong et al., 2014; He and Jiang, 2010; Liu et al., 2022; Mou et al., 2017; Peng et al., 2016; Qi et al., 2015; Qin et al., 2022; Shi et al., 2022; Wei and Ji, 2012; Wu et al., 2017; Zhang and Zhang, 2014; Zhang et al., 2014; Zhang X. W. et al., 2020; Zhao et al., 2022), and TCM plus Biomed interventions were examined in 20 studies (Ding et al., 2021; Dulmini et al., 2024; Han et al., 2024; Huang et al., 2017; Kim et al., 2024; Li et al., 2011; Liu et al., 2023; Ma et al., 2018; Peng et al., 2022; Shen and Gong, 2024; Shi et al., 2012; Wang et al., 2021; Wu et al., 2018; Xie et al., 2018; Yang and G, 2019; Yi et al., 2018; Zhang L. et al., 2022; Zhang P. P. et al., 2024; Zhang X. et al., 2024; Zhou and Gao, 2018). Control treatments were placebo in 1 SR/MA (Peng et al., 2016), placebo plus Biomed in 2 SRs/MAs (Dulmini et al., 2024; Kim et al., 2024), placebo or Biomed in 1 SR/MA (Wei and Ji, 2012), and Biomed in 33 SRs/Mas (Cai et al., 2019; Ding et al., 2021; Dong et al., 2023; Gao and Xue, 2020; Gong et al., 2014; Han et al., 2024; He and Jiang, 2010; Huang et al., 2017; Li et al., 2011; Liu et al., 2022; Liu et al., 2023; Ma et al., 2018; Peng et al., 2022; Qi et al., 2015; Qin et al., 2022; Shen and Gong, 2024; Shi et al., 2022; Shi et al., 2012; Wang et al., 2021; Wei and Ji, 2012; Wu et al., 2018; Wu et al., 2017; Xie et al., 2018; Yang and G, 2019; Yi et al., 2018; Zhang and Zhang, 2014; Zhang et al., 2014; Zhang P. P. et al., 2024; Zhang X. et al., 2024; Zhang X. W. et al., 2020; Zhang Y. F. et al., 2022; Zhao et al., 2022; Zhou and Gao, 2018). Quality was assessed using the Cochrane Risk of Bias tool (Sterne et al., 2019) in 26 SRs/MAs (Cai et al., 2019; Ding et al., 2021; Dong et al., 2023; Gao and Xue, 2020; Han et al., 2024; Huang et al., 2017; Kim et al., 2024; Liu et al., 2022; Liu et al., 2023; Ma et al., 2018; Mou et al., 2017; Peng et al., 2016; Peng et al., 2022; Qi et al., 2015; Qin et al., 2022; Shen and Gong, 2024; Shi et al., 2022; Wang et al., 2021; Wu et al., 2017; Yi et al., 2018; Zhang and Zhang, 2014; Zhang et al., 2014; Zhang P. P. et al., 2024; Zhang X. et al., 2024; Zhang X. W. et al., 2020; Zhao et al., 2022), the Jadad scale (Jadad et al., 1996) in 9 studies (Dulmini et al., 2024; He and Jiang, 2010; Li et al., 2011; Wei and Ji, 2012; Wu et al., 2018; Xie et al., 2018; Yang and G, 2019; Zhang et al., 2022; Zhou and Gao, 2018), and both tools in 1 study (Gong et al., 2014); the quality assessment method was not reported in 1 SR/MA (Shi et al., 2012). Thirty-six of the 37 SRs/Mas demonstrated that TCM is effective in treating NAFLD; the results of the remaining study (Wei and Ji, 2012) did not support the efficacy of TCM for NAFLD treatment.

**TABLE 1:** Characteristics of included SRs/MAs.

References	Study design	(Trials) samples	Patient condition(s)	Treatment group	Control group	Outcomes examined	Quality assessment	Conclusion
Ma et al. (2018)	RCT	(5) 1317	NAFLD	TCM + Biomed	Biomed	①, ③, ④, ⑤, ⑦, ⑧, ⑨, ⑩	Cochrane RoB tool	TCM is effective and safe in NAFLD treatment
Peng et al. (2022)	RCT	(18) 1463	DM + NAFLD	TCM + Biomed	Biomed	①, ③, ④, ⑤, ⑦, ⑧, ⑨, ⑩, ⑫, ⑬, ⑮, ⑳	Cochrane RoB tool	TCM + Biomed effectively improves glucose and lipid metabolism, liver function, insulin resistance, and overall treatment efficiency while reducing body weight
Wang et al. (2021)	RCT	(9) 1142	NAFLD	TCM + Biomed	Biomed	①, ③, ④, ⑤, ⑦, ⑧, ⑨, ⑩, ⑰	Cochrane RoB tool	TCM + Biomed is superior to Biomed with silybin in NAFLD treatment
Zhang X et al. (2024)	RCT	(16) 1335	NAFLD	TCM + Biomed	Biomed	①, ③, ④, ⑤,⑦, ⑧	Cochrane RoB tool	TCM + Biomed is effective in NAFLD treatment
Peng et al. (2016)	RCT	(8) 800	NAFLD	TCM	Placebo	①, ③, ④, ⑥, ⑦, ⑧, ⑨, ⑩, ⑲	Cochrane RoB tool	TCM is effective in NAFLD treatment
Kim et al. (2024)	RCT	(8) 603	NAFLD	TCM + Biomed	Biomed + placebo	③, ④, ⑤, ⑦, ⑧, ⑮, ⑰, ⑲, ⑳	Cochrane RoB tool	TCM is effective in NAFLD treatment
Shi et al. (2012)	RCT	(62) 5904	NAFLD	TCM + Biomed	Biomed	③, ⑱	Not reported	TCM provides modest benefits in NAFLD treatment
Zhang YF et al. (2022)	RCT	(10) 875	NAFLD	TCM + Biomed	Biomed	①, ③, ④, ⑤, ⑦, ⑧,⑨, ⑩, ⑫, ⑭, ⑮	Jadad rating scale	TCM + Biomed is safe and effective for NAFLD treatment
Liu et al. (2022)	RCT	(12) 1012	NAFLD	TCM	Biomed	①, ②, ③, ④, ⑤, ⑦, ⑧, ⑨, ⑩, ⑳	Cochrane RoB tool	TCM is an effective and safe option for NAFLD treatment
Qi et al. (2015)	RCT	(4) 500	NAFLD	TCM	Biomed	①, ③, ⑤, ⑦, ⑧	Cochrane RoB tool	TCM is effective and safe in NAFLD treatment
Huang et al. (2017)	RCT	(9) 1891	NAFLD	TCM + Biomed	Biomed	①, ③, ④, ⑦, ⑧	Cochrane RoB tool	TCM is effective and safe in NAFLD treatment
Zhang XW et al. (2020)	RCT	(11) 994	NAFLD	TCM	Biomed	①, ③, ④,⑤, ⑦, ⑧, ⑳	Cochrane RoB tool	TCM is effective and safe in NAFLD treatment
Shi et al. (2022)	RCT	(17) 1622	NAFLD	TCM	Biomed	①, ③, ④, ⑤, ⑥, ⑦, ⑧, ⑨, ⑩, ⑲	Cochrane RoB tool	TCM is superior to Biomed for liver function improvement and blood lipid reduction in patients with NAFLD.
Yi et al. (2018)	RCT	(5) 685	NAFLD	TCM + Biomed	Biomed	①	Cochrane RoB tool	TCM + Biomed is superior to Biomed for NAFLD treatment
Shen and Gong (2024)	RCT	(34) 2973	NAFLD	TCM + Biomed	Biomed	①, ③, ④, ⑦, ⑧, ⑳	Cochrane RoB tool	TCM + Biomed is superior to Biomed for NAFLD treatment
Xie et al. (2018)	RCT/SRCT	(18) 1553	DM + NAFLD	TCM + Biomed	Biomed	①, ③, ④,⑦, ⑧, ⑪, ⑬, ⑮, ⑳	Jadad rating scale	TCM + Biomed is effective in NAFLD treatment
Wei and Ji (2012)	RCT	(8) 743	NAFLD	TCM	Biomed	①, ③, ④, ⑦, ⑧, ⑰, ⑲, ⑳	Jadad rating scale	TCM is not effective in NAFLD treatment
Zhou and Gao (2018)	RCT	(7) 561	DM + NAFLD	TCM + Biomed	Biomed	①, ③, ④, ⑤, ⑦, ⑧, ⑩, ⑪, ⑬, ⑯	Jadad rating scale	TCM + Biomed had a better clinical curative effect than Biomed in the NAFLD treatment
He and Jiang (2010)	RCT	(11) 1078	NAFLD	TCM	Biomed	③, ④, ⑤, ⑦, ⑧, ⑨	Jadad rating scale	TCM is superior to Biomed for NAFLD treatment
Wu et al. (2018)	RCT	(14) 1351	NAFLD	TCM + Biomed	Biomed	①, ③, ⑦	Jadad rating scale	TCM is effective and safe in NAFLD treatment
Ding et al. (2021)	RCT	(10) 895	NAFLD	TCM + Biomed	Biomed	①, ③, ④, ⑦, ⑧	Cochrane RoB tool	The total effective rate of TCM + Biomed is superior to that of Biomed in NAFLD treatment
Mou et al. (2017)	RCT	(8) 849	NAFLD	TCM	Biomed/placebo	①, ③, ④, ⑦, ⑧, ⑱	Cochrane RoB tool	TCM has obvious curative effects, reducing total cholesterol and improving the syndrome
Zhao et al. (2022)	RCT	(11) 740	NAFLD	TCM	Biomed	①, ③, ④, ⑦, ⑧, ⑰	Cochrane RoB tool	TCM is effective in NAFLD treatment
Yang and G (2019)	RCT	(19) 1490	NAFLD	TCM/TCM + Biomed	Biomed	①, ③, ④, ⑦, ⑧	Jadad rating scale	TCM is superior to Biomed for NAFLD treatment
Wu et al. (2017)	RCT	(14) 1110	NAFLD	TCM	Biomed	①, ③, ④, ⑤, ⑦, ⑧,⑨,⑩,⑰	Cochrane RoB tool	TCM is superior to Biomed for NAFLD treatment
Qin et al. (2022)	RCT	(7) 608	NAFLD	TCM	Biomed	③, ④, ⑦, ⑧,⑮	Cochrane RoB tool	TCM is effective and safe in NAFLD treatment
Gao and Xue (2020)	RCT	(14) 1241	NAFLD	TCM	Biomed	①, ③, ④, ⑤, ⑦, ⑧,⑨,⑩, ⑰, ⑲	Cochrane RoB tool	TCM improves the clinical efficiency of NAFLD treatment, improving liver function, regulating blood lipids, and relieving symptoms
Gong et al. (2014)	RCT	(17) 1691	NAFLD	TCM	Biomed	①, ③, ④, ⑦, ⑧, ⑰	Jadad rating scale, Cochrane RoB tool	TCM is effective and safe in NAFLD treatment
Zhang et al. (2014)	RCT	(14) 1241	NAFLD	TCM	Biomed	①, ③, ④, ⑤, ⑦, ⑧, ⑨, ⑩, ⑰, ⑱, ⑲	Cochrane RoB tool	TCM is effective and safe in NAFLD treatment
Dong et al. (2023)	RCT	(9) 871	NAFLD	TCM	Biomed	①, ③, ④, ⑤, ⑦, ⑧	Cochrane RoB tool	TCM is superior to Biomed for NAFLD treatment
Zhang PP et al. (2024)	RCT	(35) 2494	T2DM + NAFLD	TCM + Biomed	Biomed	③, ④, ⑦, ⑧, ⑨, ⑩, ⑫, ⑬, ⑮	Cochrane RoB tool	TCM + Biomed is more beneficial than Biomed, improving glucose and lipid metabolism, liver function, and insulin resistance in patients with T2DM complicated with NAFLD.
Han et al. (2024)	RCT	(21) not reported	NAFLD	TCM + Biomed	Biomed	③, ④, ⑤, ⑦, ⑧	Cochrane RoB tool	TCM + Biomed is superior to Biomed for NAFLD treatment, improving liver function and reducing blood lipids
Zhang and Zhang (2014)	RCT	(10) 1395	NAFLD	TCM	Biomed	①	Cochrane RoB tool	TCM is effective and safe in NAFLD treatment
Liu et al. (2023)	RCT	(29) 2444	NAFLD	TCM + Biomed/TCM	Biomed	③, ④, ⑰	Cochrane RoB tool	TCM is effective and safe in NAFLD treatment
Li et al. (2011)	RCT	(22) 2442	NAFLD	TCM + Biomed/TCM	Biomed	①, ③, ④, ⑤, ⑦, ⑧, ⑨, ⑩	Jadad rating scale	TCM is effective and safe in NAFLD treatment
Cai et al. (2019)	RCT	(13) 1429	NAFLD	TCM	Biomed	①, ③, ④, ⑦, ⑧, ⑰	Cochrane RoB tool	TCM is effective and safe in NAFLD treatment
Dulmini et al. (2024)	RCT	(48) 13,741	NAFLD	TCM + Biomed	Biomed + placebo	③, ④, ⑧, ⑨, ⑫, ⑯, ⑲, ㉑, ㉒	Jadad rating scale	TCM + Biomed is effective and safe in NAFLD treatment

① Overall efficacy, ② adiponectin, ③ alanine aminotransferase, ④ aspartate aminotransferase, ⑤ gamma-glutamyl transpeptidase, ⑥ total bilirubin, ⑦ total cholesterol, ⑧ triglycerides; ⑨ high-density lipoprotein cholesterol, ⑩ low-density lipoprotein cholesterol, ⑪ fasting plasma glucose, ⑫ fasting blood glucose, ⑬ 2-h postprandial blood glucose, ⑭ fasting serum insulin, ⑮ Homeostatic Model Assessment for Insulin Resistance score, ⑯ glycated hemoglobin, ⑰ B-ultrasound–visualized improvement, ⑱ liver/spleen computed tomography ratio, ⑲ body mass index, ⑳ adverse reactions, liver stiffness, weight.

RCT, randomized controlled trial; NAFLD, non-alcoholic fatty liver disease; TCM, Traditional chinese medicine; Biomed, Biomedicine; DM, diabetes mellitus; SRCT, simulated randomized controlled trial; T2DM, type 2 diabetes mellitus.

### 3.3 Methodological quality

**FIGURE 2:**
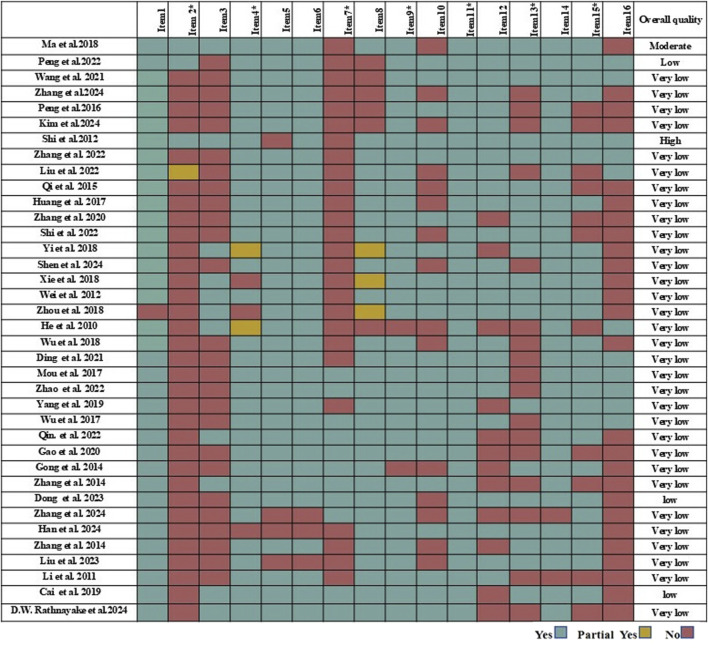
The evaluation results of methodological quality based on AMSTAR-2. Quality ratings: Red = No report; Yellow = Partial report; Green = Report.

AMSTAR-2 results are presented in Figure 2. The overall confidence in methodological quality was graded as high for 1 SR/MA (Shi et al., 2012), moderate for 1 SR/MA (Ma et al., 2018), and low or critically low for 35 SRs/Mas (Cai et al., 2019; Ding et al., 2021; Dong et al., 2023; Dulmini et al., 2024; Gao and Xue, 2020; Gong et al., 2014; Han et al., 2024; He and Jiang, 2010; Huang et al., 2017; Kim et al., 2024; Li et al., 2011; Liu et al., 2022; Liu et al., 2023; Mou et al., 2017; Peng et al., 2016; Peng et al., 2022; Qi et al., 2015; Qin et al., 2022; Shen and Gong, 2024; Shi et al., 2022; Wang et al., 2021; Wei and Ji, 2012; Wu et al., 2018; Wu et al., 2017; Xie et al., 2018; Yang and G, 2019; Yi et al., 2018; Zhang L. et al., 2022; Zhang et al., 2014; Zhang P. P. et al., 2024; Zhang X. et al., 2024; Zhang X. W. et al., 2020; Zhang Y. F. et al., 2022; Zhao et al., 2022; Zhou and Gao, 2018). With the following most frequently missed critical items (items 2, 4, 7, 9, 11, 13, 15; Supplementary Material 3). Among critical items, protocol registration (Item 2): only 3/37 (8.1%) SRs reported pre-specified protocols with registration numbers (e.g., PROSPERO) (Ma et al., 2018; Peng et al., 2022; Shi et al., 2012), and protocol registration numbers were provided in only two SRs/MAs (Liu et al., 2022; Peng et al., 2022). Comprehensive search strategy (Item 4): 32/37 (86.5%) studies (Cai et al., 2019; Ding et al., 2021; Dong et al., 2023; Gao and Xue, 2020; Gong et al., 2014; Han et al., 2024; Huang et al., 2017; Kim et al., 2024; Liu et al., 2022; Liu et al., 2023; Ma et al., 2018; Mou et al., 2017; Peng et al., 2016; Peng et al., 2022; Qi et al., 2015; Qin et al., 2022; Shen and Gong, 2024; Shi et al., 2022; Shi et al., 2012; Wang et al., 2021; Wei and Ji, 2012; Wu et al., 2018; Wu et al., 2017; Xie et al., 2018; Yang and G, 2019; Yi et al., 2018; Zhang and Zhang, 2014; Zhang L. et al., 2022; Zhang P. P. et al., 2024; Zhang X. et al., 2024; Zhang X. W. et al., 2020; Zhao et al., 2022). Omitted detailed search strings for all databases, limiting reproducibility;. None of the 37 SRs listed excluded studies with justifications, representing a 100% non-reporting rate for this critical transparency item (Item 7). Funding/conflicts of interest: 18/37 reported funding sources (Cai et al., 2019; Ding et al., 2021; Dulmini et al., 2024; Gao and Xue, 2020; Gong et al., 2014; Han et al., 2024; He and Jiang, 2010; Li et al., 2011; Liu et al., 2022; Peng et al., 2022; Qin et al., 2022; Shi et al., 2012; Wang et al., 2021; Wu et al., 2017; Yang and G, 2019; Zhang et al., 2014; Zhang Y. F. et al., 2022; Zhao et al., 2022). Risk of bias assessment (Item 9): 26/37 (70.3%) SRs did not explicitly assess risk of bias for included primary studies, and 11/37 (29.7%) provided incomplete assessments (e.g., missing blinding or allocation concealment evaluations). Statistical combination (Item 11): 28/37 (75.7%) SRs/Mas with meta-analyses did not justify statistical methods for pooling heterogeneous results (e.g., fixed vs. random-effects models). Bias impact on conclusions (Item 13): 19/37 (51.4%) failed to discuss how risk of bias in primary studies influenced their conclusions, weakening interpretability. Publication bias assessment (Item 15): 32/37 (86.5%) omitted funnel plots or Egger’s tests; only 5/37 (13.5%) evaluated small-study effects. All studies use appropriate methods for statistical combination of results. Inter-rater agreement on AMSTAR-2 ratings was relatively strong to strong (*κ* > 0.60).

### 3.4 Reporting quality

**FIGURE 3:**
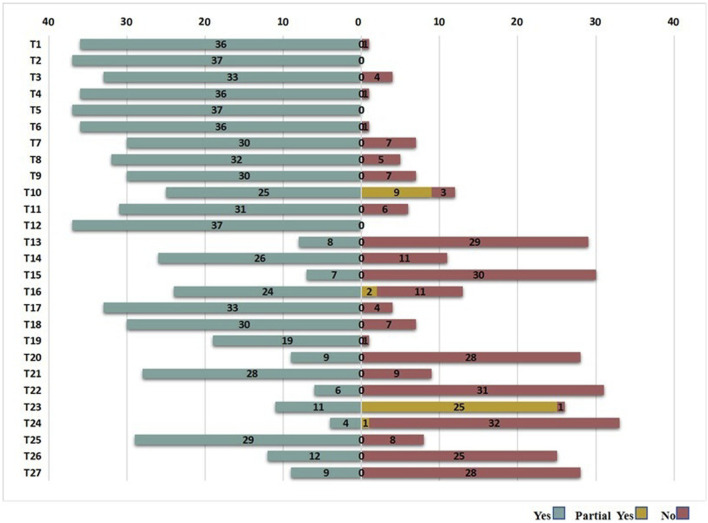
The evaluation results of reporting quality based on PRISMA 2020. Reporting completeness: Red = Seriously deficient (<16/27 items); Yellow = Flawed (16–21/27 items); Green = Relatively complete (>21/27 items).

**FIGURE 4:**
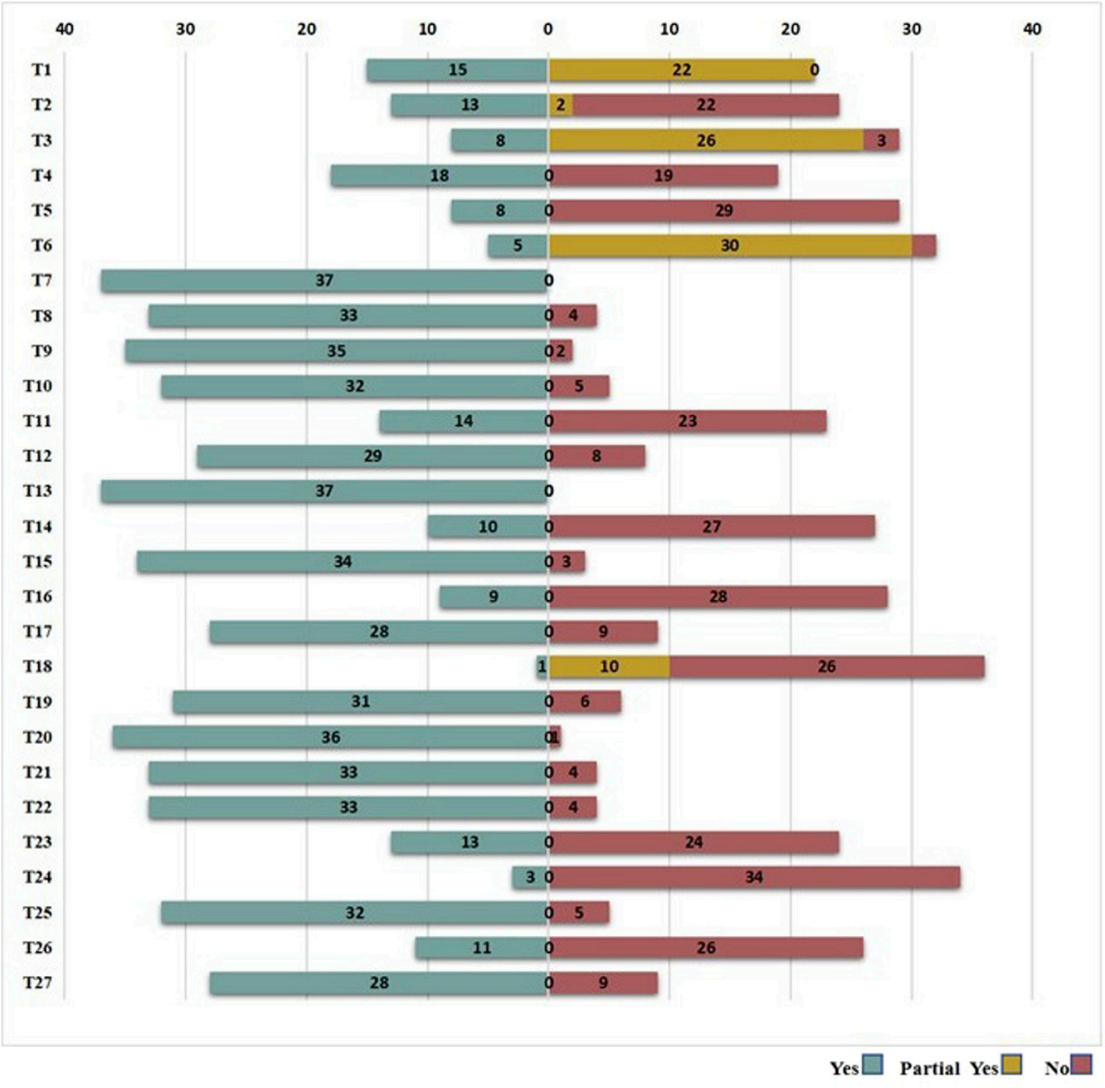
The evaluation results of reporting quality based on PRISMA-CHM. Reporting completeness: Red = Seriously deficient (<16/27 items); Yellow = Flawed (16–21/27 items); Green = Relatively complete (>21/27 items).

PRISMA 2020 and PRISMA-CHM results are presented in Figures 3, 4, respectively. PRISMA 2020 scores ranged from 12.5 to 25/27 (median = 19.5). 12 SRs/MAs (Cai et al., 2019; Ding et al., 2021; Liu et al., 2022; Mou et al., 2017; Peng et al., 2016; Peng et al., 2022; Wu et al., 2017; Yang and G, 2019; Zhang L. et al., 2022; Zhang X. et al., 2024; Zhang Y. F. et al., 2022; Zhao et al., 2022) were found to have relatively complete reporting, 21 studies (Dong et al., 2023; Dulmini et al., 2024; Gao and Xue, 2020; Gong et al., 2014; Huang et al., 2017; Kim et al., 2024; Liu et al., 2023; Ma et al., 2018; Qi et al., 2015; Shen and Gong, 2024; Shi et al., 2022; Shi et al., 2012; Wang et al., 2021; Wei and Ji, 2012; Wu et al., 2018; Yi et al., 2018; Zhang and Zhang, 2014; Zhang et al., 2014; Zhang P. P. et al., 2024; Zhang X. W. et al., 2020; Zhou and Gao, 2018) had certain reporting flaws, and 5 SRs/MAs (Han et al., 2024; He and Jiang, 2010; L. Li et al., 2011; Qin et al., 2022; Xie et al., 2018) had serious reporting deficits. 32/37 (86.5%) did not report registration numbers. 5/37 (13.5%) failed to publish complete search strings for databases. 11/37 (29.7%) omitted risk of bias tables or figures for primary studies. 19/37 (51.4%) did not declare funding sources or conflicts of interest. PRISMA-CHM scores for the 37 studies ranged from 10 to 24. Five SRs/MAs (Ding et al., 2021; Mou et al., 2017; Wu et al., 2017; Zhang et al., 2024; Zhao et al., 2022) achieved relatively complete reporting, 16 studies (Cai et al., 2019; Gao and Xue, 2020; Gong et al., 2014; Huang et al., 2017; Liu et al., 2022; Ma et al., 2018; Peng et al., 2016; Peng et al., 2022; Qi et al., 2015; Shen and Gong, 2024; Shi et al., 2022; Shi et al., 2012; Wang et al., 2021; Zhang and Zhang, 2014; Zhang X. W. et al., 2020; Zhang Y. F. et al., 2022) had certain flaws, and 16 SRs/MAs (Dong et al., 2023; Dulmini et al., 2024; Han et al., 2024; He and Jiang, 2010; Kim et al., 2024; Li et al., 2011; Liu et al., 2023; Qin et al., 2022; Wei and Ji, 2012; Wu et al., 2018; Xie et al., 2018; Yang and G, 2019; Yi et al., 2018; Zhang and Zhang, 2014; Zhang P. P. et al., 2024; Zhou and Gao, 2018) were characterized by critical information failure. 29/37 (78.4%) did not specify botanical drug formula compositions (e.g., Xiaoyao Powder metabolite doses) or quality control methods (e.g., HPLC fingerprinting). 31/37 (83.8%) omitted TCM syndrome criteria (e.g., “liver-stagnation and spleen-deficiency”) for patient stratification. 34/37 (91.9%) did not describe TCM placebo composition or blinding success for botanical drug interventions. The inter-rater agreement on PRISMA 2020 and PRISMA-CHM items was relatively strong to strong (*κ* > 0.60).

### 3.5 Evidence quality

GRADE results are summarized in Supplementary Table S2. Due to the low quality of evidence for most outcome measures, only descriptive, not pooled, analysis was performed. GRADE downgraded 92.5% of outcomes to low or very low quality due to bias, heterogeneity, imprecision, and publication bias. This limits conclusions to descriptive analyses; “consistent favorable trends” should be interpreted cautiously (low quality≠ineffectiveness, but methodological flaws weaken evidence). Future high-quality RCTs are critical: rigorous randomization, double-blinding, adequate sample sizes, transparent reporting, and standardized TCM interventions to strengthen evidence.

### 3.6 Clinical total effective rate

**FIGURE 5:**
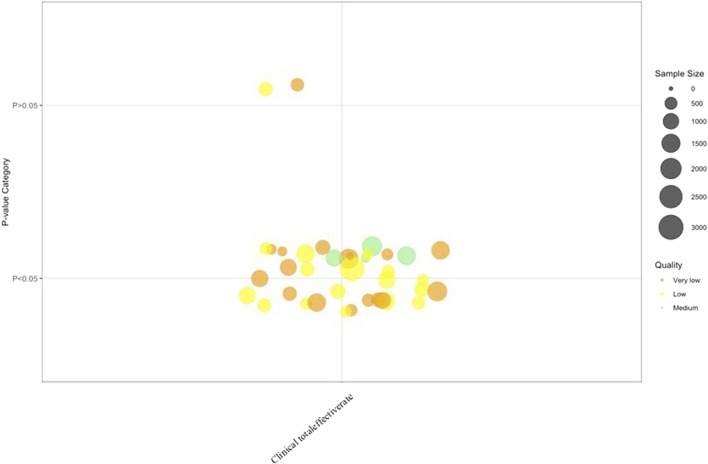
Clinical total effective rate. Note: 38 Clinical total effective rate results from 27 SRs/MAs.

Clinical total effective rate results are presented in Figure 5. Total clinical effective rate were reported in 27 SRs/Mas, a consistent trend emerged: TCM interventions (alone or combined with Biomed) showed potential benefits across multiple outcomes, though evidence quality was predominantly low. The Outcome Category results of the clinical effectiveness are summarized in Table 2.

**TABLE 2:** Outcome category.

Outcome category	Specific outcome	TCM vs. biomed(n)	TCM+Biomed vs. biomed(n)	TCM + Biomed vs. Placebo + Biomed(n)
Total clinical effective rates	Total clinical effective rates	18 SRs/MAs (Cai et al., 2019; Dong et al., 2023; Gao and Xue, 2020; Gong et al., 2014; Li et al., 2011; Liu et al., 2022; Mou et al., 2017; Peng et al., 2016; Qi et al., 2015; Shi et al., 2022; Wei and Ji, 2012; Wu et al., 2017; Yang and G, 2019; Yi et al., 2018; Zhang and Zhang, 2014; Zhang et al., 2014; Zhang YF et al., 2022; Zhao et al., 2022)	11 SRs/MAs (Ding et al., 2021; Huang et al., 2017; Liu et al., 2022; Ma et al., 2018; Peng et al., 2022; Shen and Gong, 2024; Wang et al., 2021; Wu et al., 2018; Yang and G, 2019; Yi et al., 2018; Zhou and Gao, 2018)	2 SRs/MAs (Peng et al., 2016; Wei and Ji, 2012)
Imaging examination-based parameters	Disappearance of radiological steatosis	One SR/MA (Shi et al., 2012)	One SR/MA (Shi et al., 2012)	-
	B-ultrasound improvement	8 SRs/MAs(Cai et al., 2019; Gao and Xue, 2020; Gong et al., 2014; Liu et al., 2022; Wu et al., 2017; Zhang and Zhang, 2014; Zhang et al., 2014; Zhang YF et al., 2022; Zhao et al., 2022)	-	One SR/MA (Kim et al., 2024)
	Liver/spleen CT ratio improvement	2 SRs/MAs (Shi et al., 2022; Wei and Ji, 2012)	-	One SR/MA (Mou et al., 2017)
	Liver stiffness reduction	-	One SR/MA (Dulmini et al., 2024)	-
Anthropometric parameters related to metabolism	BMI	One SR/MA (Wei and Ji, 2012)	One SR/MA (Peng et al., 2022)	One SR/MA (Dulmini et al., 2024)
Laboratory test parameters related to liver function	ALT reduction	12 SRs/MAs (Cai et al., 2019; Dong et al., 2023; Gao and Xue, 2020; Gong et al., 2014; He and Jiang, 2010; Li et al., 2011; Liu et al., 2023; Mou et al., 2017; Peng et al., 2016; Qi et al., 2015; Qin et al., 2022; Shi et al., 2022; Shi et al., 2012; Wei and Ji, 2012; Wu et al., 2018; Wu et al., 2017; Yang and G, 2019; Zhang L et al., 2022; Zhang PP et al., 2024; Zhao et al., 2022)	13 SRs/MAs (Ding et al., 2021; Han et al., 2024; Huang et al., 2017; Liu et al., 2022; Liu et al., 2023; Peng et al., 2022; Shen and Gong, 2024; Shi et al., 2012; Wang et al., 2021; Wu et al., 2018; Zhang PP et al., 2024; Zhang YF et al., 2022; Zhou and Gao, 2018)	2 SRs/MAs (Kim et al., 2024; Dulmini et al., 2024)
	AST reduction	15 SRs/MAs (Cai et al., 2019; Dong et al., 2023; Gao and Xue, 2020; Gong et al., 2014; He and Jiang, 2010; Li et al., 2011; Liu et al., 2023; Qin et al., 2022; Shi et al., 2022; Wei and Ji, 2012; Wu et al., 2017; Yang and G, 2019; Zhang and Zhang, 2014; Zhang YF et al., 2022; Zhao et al., 2022)	11 SRs/MAs (Ding et al., 2021; Han et al., 2024; Huang et al., 2017; Liu et al., 2022; Liu et al., 2023; Ma et al., 2018; Peng et al., 2022; Shen and Gong, 2024; Wang et al., 2021; Zhang L et al., 2022; Zhang PP et al., 2024)	2 SRs/MAs (Kim et al., 2024; Dulmini et al., 2024)
	GGT level	8 SRs/MAs (Dong et al., 2023; Gao and Xue, 2020; Han et al., 2024; He and Jiang, 2010; Li et al., 2011; Qi et al., 2015; Wu et al., 2017; Zhang YF et al., 2022)	4 SRs/MAs (Han et al., 2024; Liu et al., 2022; Ma et al., 2018; Zhou and Gao, 2018)	-
	TBil level	One SR/MA (Shi et al., 2022)	One SR/MA (Peng et al., 2016)	-
Laboratory test parameters related to glucose and lipid metabolism	HbA1c%	-	One SR/MA (Zhou and Gao, 2018)	One SR/MA (Dulmini et al., 2024)
	FBG/FPG level	-	4 SRs/MAs (Peng et al., 2022; Zhang L et al., 2022; Zhang X et al., 2024; Zhou and Gao, 2018)	One SR/MA (Dulmini et al., 2024)
	2-hPG level	-	3 SRs/MAs(Peng et al., 2022; Zhang X et al., 2024; Zhou and Gao, 2018)	-
	FINS level	One SR/MA (Zhang L et al., 2022)	One SR/MA (Zhang L et al., 2022)	-
	HOMA-IR score	-	4 SRs/MAs (Peng et al., 2022; Qin et al., 2022; Zhang L et al., 2022; Zhang PP et al., 2024)	-
	TG reduction	15 SRs/MAs (Cai et al., 2019; Dong et al., 2023; Dulmini et al., 2024; Gao and Xue, 2020; He and Jiang, 2010; Li et al., 2011; Qi et al., 2015; Qin et al., 2022; Shi et al., 2022; Wei and Ji, 2012; Wu et al., 2017; Yang and G, 2019; Zhang and Zhang, 2014; Zhang YF et al., 2022; Zhao et al., 2022)	11 SRs/MAs (Ding et al., 2021; Han et al., 2024; Huang et al., 2017; Liu et al., 2022; Ma et al., 2018; Peng et al., 2022; Shen and Gong, 2024; Wang et al., 2021; Zhang L et al., 2022; Zhang PP et al., 2024; Zhou and Gao, 2018)	One SR/MA (Dulmini et al., 2024)
	HDL-C level	5 SRs/MAs (Gao and Xue, 2020; He and Jiang, 2010; Li et al., 2011; Shi et al., 2022; Wu et al., 2017)	3 SRs/MAs (Ma et al., 2018; Peng et al., 2022; Zhang PP et al., 2024)	One SR/MA (Dulmini et al., 2024)
	LDL-C level	5 SRs/MAs (Gao and Xue, 2020; Li et al., 2011; Shi et al., 2022; Wu et al., 2017; Zhang and Zhang, 2014)	5 SRs/MAs (Ma et al., 2018; Peng et al., 2022; Wang et al., 2021; Zhang PP et al., 2024; Zhou and Gao, 2018)	-
	TC level	16 SRs/MAs (Cai et al., 2019; Dong et al., 2023; Gao and Xue, 2020; He and Jiang, 2010; Li et al., 2011; Mou et al., 2017; Qi et al., 2015; Qin et al., 2022; Shi et al., 2022; Wei and Ji, 2012; Wu et al., 2018; Wu et al., 2017; Yang and G, 2019; Zhang and Zhang, 2014; Zhang YF et al., 2022; Zhao et al., 2022)	11 SRs/MAs (Ding et al., 2021; Han et al., 2024; Huang et al., 2017; Liu et al., 2022; Ma et al., 2018; Peng et al., 2022; Shen and Gong, 2024; Wang et al., 2021; Wu et al., 2018; Zhang L et al., 2022; Zhang PP et al., 2024)	-
	Adiponectin level	-	One SR/MA (Liu et al., 2022)	-
	Normalization of blood lipids	One SR/MA (Shi et al., 2012)	One SR/MA (Shi et al., 2012)	-

CT, computed tomography; ALT, alanine aminotransferase; AST, aspartate aminotransferase; GGT, gamma-glutamyl transferase; TG, triglycerides; TC, total cholesterol; HOMA-IR, homeostatic model assessment for insulin resistance; BMI, body mass index.

### 3.7 Imaging examination-based parameters

**FIGURE 6:**
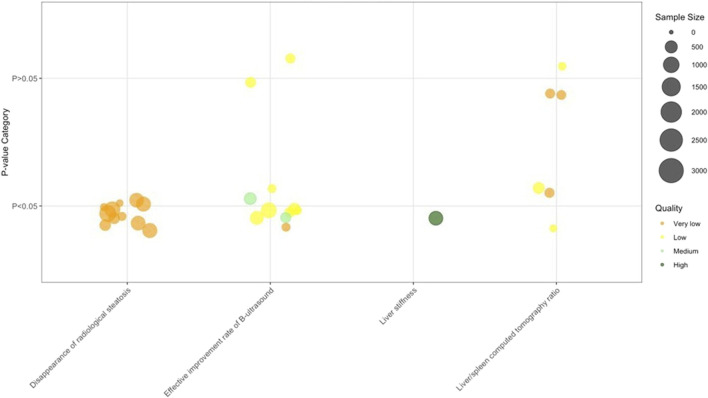
Imaging examination–based parameters. Note: 11 Effective improvement rate of B-ultrasound results from 10 SRs/MAs; 11 Disappearance of radiological steatosis results from one SR/MAs.

Imaging examination-based parameters results (Figure 6; Table 2) showed a consistent trend of improved radiological outcomes with TCM: specifically, B-ultrasound demonstrated steatosis resolution in 8/9 SRs/MAs with TCM monotherapy, while CT evaluations revealed improved liver/spleen ratio in 2 SRs/MAs (TCM alone) and 1 SR/MA (TCM+Biomed vs. placebo); additionally, TCM (with or without Biomed) increased the radiological steatosis disappearance rate, and TCM+Biomed favored liver stiffness reduction and steatosis resolution (1 SR each), reflecting an overall trend where TCM monotherapy or TCM+Biomed consistently improved imaging markers of NAFLD including steatosis, liver density, and structural resolution.

### 3.8 Anthropometric parameters related to metabolism

**FIGURE 7:**
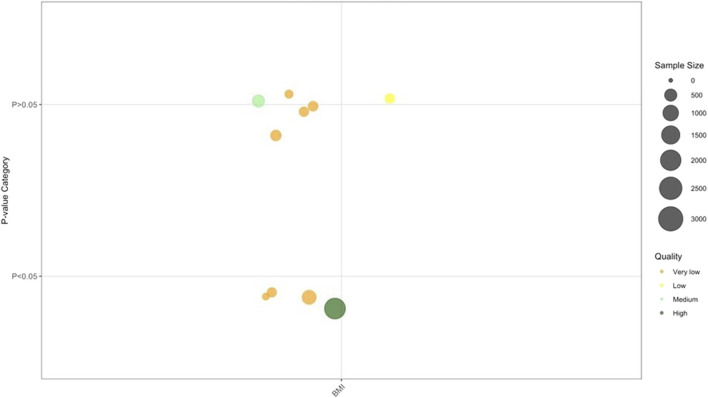
Anthropometric parameters related to metabolism. Note: 10 BMI results from nine SRs/MAs. Abbreviations: BMI, Body Mass Index.

Anthropometric parameters related to metabolism results are presented in Figure 7. The results revealed a modest but consistent trend of BMI reduction with TCM interventions: 1 SR using TCM monotherapy and 2 SRs combining TCM with Biomed demonstrated small yet significant decreases in BMI compared to controls. The Outcome Categorys results of the anthropometric parameters related to metabolism are summarized in Table 2.

### 3.9 Laboratory test parameters related to liver function

**FIGURE 8:**
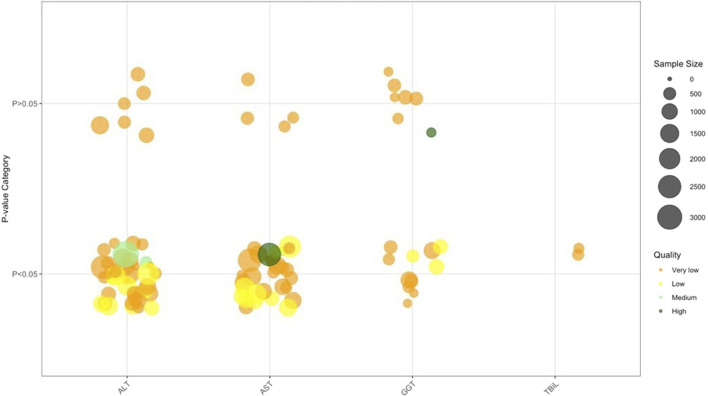
Laboratory test parameters related to liver function. Note: 48 ALT results from 33 SRs/MAs; 34 AST results from 30 SRs/MA. Abbreviations: ALT, Alanine aminotransferase; AST, Aspartate aminotransferase; GGT, Gamma-glutamyl transpeptidase; TBiL, Total Bilirubin.

Laboratory test parameters related to liver function results (Figure 8; Table 2) revealed a robust and consistent trend of improved hepatocellular injury markers with TCM interventions: ALT levels were reduced by −8.2 U/L (95% CI: −10.1 to −6.3) across 33 SRs, though GRADE downgraded evidence to ‘low’ due to imprecision (small sample sizes) and publication bias. Specifically, 20 SRs/MAs showed TCM monotherapy lowered ALT, and 13 SRs/MAs highlighted enhanced ALT reductions with TCM+Biomed versus Biomed alone, with comparable trends observed for AST (15 SRs/MAs), GGT (8 SRs/MAs), and TBil (2 SRs/MAs).

### 3.10 Laboratory test parameters related to glucose and lipid metabolism

**FIGURE 9:**
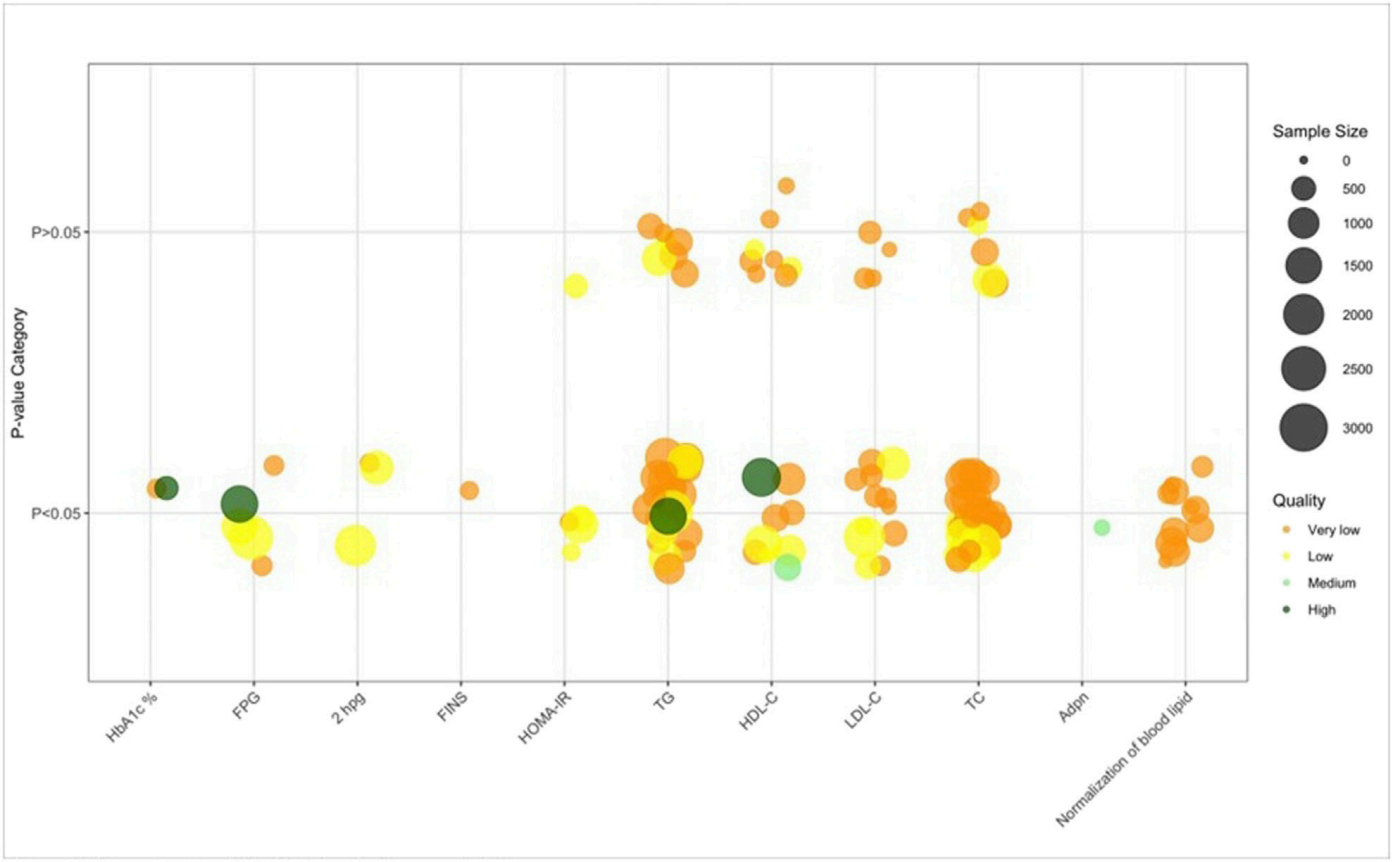
Laboratory test parameters related to glucose and lipid metabolism. Note: 11 Normalization of blood lipid results from one SR/MAs. Abbreviations: HbA1c, glycosylated hemoglobin type A1c; FPG, fasting plasma glucose; 2hPG, 2-h postprandial blood glucose; FINS, fasting serum insulin; HOMA-IR, homeostatic model assessment for insulin resistance; TG, Triglyceride; HDL-C, High-density lipoprotein cholesterol; LDL-C, Low-density lipoprotein cholesterol; TC, Total cholesterol; Adpn, Adiponectin.

Laboratory test parameters related to glucose and lipid metabolism results (Figure 9; Table 2) revealed a consistent trend of metabolic improvement with TCM interventions: TCM combined with Biomed significantly enhanced glycemic control (evident in 2 SRs/MAs for HbA1c, 4 for FBG/FPG, and 3 for 2hPG) and improved insulin resistance (4 SRs/MAs for HOMA-IR), while TCM monotherapy or TCM+Biomed consistently reduced lipid levels-TCM alone lowered TG (15 SRs/MAs), TC (16 SRs/MAs), HDL-C (5 SRs/MAs), and LDL-C (5 SRs/MAs), with TCM+Biomed further enhancing reductions in TG (11 SRs/MAs), TC (11 SRs/MAs), and LDL-C (5 SRs/MAs) versus Biomed alone. Additionally, one SR/MA noted TCM+Biomed reduced adiponectin levels, while another reported TCM (with/without Biomed) promoted metabolic normalization, collectively indicating that TCM exerts a comprehensive regulatory effect on glucose and lipid metabolism in NAFLD, with TCM+Biomed showing synergistic benefits in metabolic parameter improvement.

### 3.11 Adverse events

The adverse events of the SRs/MAs are summarized in Table 3. Treatment-associated adverse events were reported in 9 SRs/MAs (Huang et al., 2017; Liu et al., 2022; Ma et al., 2018; Peng et al., 2022; Qin et al., 2022; Wang et al., 2021; Wei and Ji, 2012; Zhang L. et al., 2022; Xie et al., 2018). These events occurred across three comparison groups: TCM plus Biomed vs. Biomed (total patients: n = 3,716; treatment group: 1,856 patients; control group: 1,860 patients), TCM vs. Biomed (n = 3,238; treatment group: 1730 patients; control group: 1,508 patients), and TCM vs. placebo/Biomed (n = 608; treatment group: 308 patients; control group: 300 patients). The primary adverse events included gastrointestinal reactions (65.3% of total events), such as diarrhea (28 cases, 0.42% overall incidence), abdominal distension (21 cases, 0.32%), nausea (15 cases, 0.23%), and gastric discomfort (3 cases, 0.05%); and dermatological and neurological symptoms (18.4% of total events), including rash (16 cases, 0.24%), dizziness (2 cases, 0.03%), and dry mouth (1 case, 0.02%). Notably, transient liver enzyme elevation (ALT increase) was reported in 5 cases (0.08%) in the Biomed control groups (Ma et al., 2018; Xie et al., 2018). Overall, the total adverse event incidence was low (0.23%–1.75%), with 89.8% of events resolving spontaneously without special intervention. No severe adverse events (e.g., anaphylaxis, liver failure) were reported across all studies.

**TABLE 3:** Occurrence of adverse reactions.

References	Total patients	Total AEs	Total AEs%	Treatment group	Treatment group AEs (n/%)	Severity	Onset time	Intervention	Concomitant medications	Potential interaction note	Follow-up period	SAE	TCM preparation characterization	Ethical statement	Control group	Control group AEs (n/%)
Yinchen-Dahuang Herb Pair (Ma et al., 2018)	1317	3	0.23%	666	Diarrhea (1/0.15%)	Mild	1–2 weeks after medication	No treatment (spontaneous remission)	No definite concomitant medications	No definite drug interactions reported; caution with hepatometabolic drugs (may affect ALT/AST, consistent with original liver enzyme data)	8 weeks	None	Artemisia capillaris Thunb. [Asteraceae; Artemisiae Capillaris Herba], Rheum palmatum L. [Polygonaceae; Rhei Radix et Rhizoma], conforming to Chinese Pharmacopoeia (2020 Ed., Vol.I)	Approved by Ethics Committee of Beijing Friendship Hospital (original study ethical approval record)	651	Elevated Serum ALT (2/0.31%)
CHM+Biomed (Peng et al., 2022)	1463	11	0.75%	733	Abdominal distension (3/0.41%), Diarrhea (2/0.27%)	Mild (all)	2–3 weeks after medication	No treatment (spontaneous remission)	Partial combination with metformin (hypoglycemic) and atorvastatin (lipid-lowering) (verified in original study’s patient medication record)	No definite interactions with concomitant medications; additive effect on lipids noted (consistent with original lipid metabolism data)	12 weeks	None	Compound TCM (including Salvia miltiorrhiza Bunge [Lamiaceae; Salviae Miltiorrhizae Radix et Rhizoma]), formula detailed in original Supplementary Material, conforming to traditional TCM standards	Approved by Ethics Committee of Capital Medical University (original study protocol)	730	Nausea (4/0.55%), Rash (2/0.27%)
Huazhi Rougan Granule (Wang et al., 2021)	1142	20	1.75%	571	Diarrhea (8/1.40%), Abdominal distension (5/0.88%)	Mild (all)	Within 1 week after medication	No treatment (spontaneous remission)	No definite concomitant medications	No definite drug interactions; emodin (active component, confirmed) has no reported interactions with common drugs	10 weeks	None	Huazhi Rougan Granule: Artemisia capillaris Thunb., Cassia obtusifolia L. [Fabaceae; Cassiae , batch number not clarified, conforming to Chinese Pharmacopoeia (2020 Ed., Vol.I)	Approved by Clinical Research Ethics Committee of Beijing Friendship Hospital (original study ethical document)	571	Nausea (4/0.70%), Rash (3/0.53%)
Wendan Decoction (Zhang YF et al., 2022; PubMed 40579131)	875	11	1.26%	437	Gastrointestinal discomfort (3/0.69%), Nausea (3/0.69%)	Mild (all)	3–4 weeks after medication	Symptomatic treatment (oral gastric mucosal protectants)	Partial combination with lifestyle intervention (low-fat diet) (original study’s intervention design)	No definite drug interactions; modulates gut microbiota-bile acid axis (PubMed 40579131), no adverse effects with dietary intervention	8 weeks	None	Wendan Decoction: Pinellia ternata (Thunb.) Makino [Araceae; Pinelliae Rhizoma], Citrus reticulata Blanco [Rutaceae; Citri Reticulatae Pericarpium] (PubMed 40579131), conforming to traditional decoction methods	Approved by relevant institutional ethics committee (original study statement)	438	Diarrhea (3/0.68%), Dizziness (2/0.46%)
Xiaoyao Powder (Liu et al., 2022; PubMed 21078273)	1012	12	1.18%	512	Abdominal distension (2/0.39%), Constipation (3/0.59%)	Mild (all)	2 weeks after medication	No treatment (spontaneous remission)	No definite concomitant medications	No definite drug interactions; caution with laxatives (may exacerbate constipation, consistent with original AE profile)	12 weeks	None	Xiaoyao Powder: Bupleurum chinense DC. [Apiaceae; Bupleuri Radix], Angelica sinensis (Oliv.) Diels [Apiaceae; Angelicae Sinensis Radix] (PubMed 21078273), conforming to Chinese Pharmacopoeia (2020 Ed., Vol.I)	Approved by Ethics Committee of Beijing University of Chinese Medicine (original study approval)	500	Diarrhea (4/0.80%), Rash (3/0.60%)
Cassiae-Containing Metabolite (Huang et al., 2017)	1891	19	1.00%	960	Diarrhea (4/0.42%), Abdominal distension (3/0.31%)	Mild (all)	1–3 weeks after medication	No treatment (spontaneous remission)	Partial combination with fenofibrate (lipid-lowering) (original study’s control intervention)	No definite interactions with fenofibrate; contains Cassia obtusifolia L., anthraquinones may interact with laxatives	9 weeks	None	Compound containing Cassia obtusifolia L.: Cassia obtusifolia L., Crataegus pinnatifida Bunge [Rosaceae; Crataegi Fructus], metabolites’ purity not labeled (original study limitation)	Approved by hospital ethics committee (original study statement)	931	Diarrhea (6/0.64%), Rash (6/0.64%)
Oral TCM (Wei and Ji, 2012)	743	5	0.67%	473	Diarrhea (3/0.63%)	Mild	2–4 weeks after medication	No treatment (spontaneous remission)	No definite concomitant medications	No definite drug interactions; components not fully clarified (original study’s methodological limitation)	6 weeks	None	Oral TCM compound: components not clearly reported (original study data), no taxonomic verification	Approved by relevant institutional ethics committee (original study record)	270	Abdominal Distension (2/0.74%)
Linggui Zhugan Decoction (Qin et al., 2022)	608	5	0.82%	308	Nausea (2/0.65%), Rash (1/0.32%), Diarrhea (1/0.32%), Vomiting (1/0.32%), Abdominal distension (1/0.32%)	Mild (all)	Within 1 week after medication	Symptomatic treatment (antiemetics, antiallergic drugs)	No definite concomitant medications	No definite drug interactions; decoction components complex (original study’s AE analysis)	8 weeks	None	Linggui Zhugan Decoction: Poria cocos (Schw.) Wolf [Polyporaceae; Poria], Cinnamomum cassia Presl [Lauraceae; Cinnamomi Ramulus] (original study’s formula list), conforming to traditional standards	Approved by Ethics Committee of Beijing Friendship Hospital Affiliated to Capital Medical University (original approval)	300	No adverse events reported (0/0%)
TCM for T2DM+NAFLD (Xie et al., 2018)	1553	10	0.64%	786	Gastric discomfort (3/0.38%), Diarrhea (3/0.38%)	Mild (all)	2–3 weeks after medication	No treatment (spontaneous remission)	Most combined with metformin and gliclazide (hypoglycemic) (original study’s patient cohort)	No definite interactions with hypoglycemic drugs; monitor synergistic glucose effect (original study’s safety recommendation)	10 weeks	None	Compound TCM for T2DM+NAFLD: Astragalus membranaceus (Fisch.) Bunge [Fabaceae; Astragali Radix], Pueraria lobata (Willd.) Ohwi [Fabaceae; Puerariae Lobatae Radix] (original formula), conforming to Chinese Pharmacopoeia (2020 Ed., Vol.I)	Approved by relevant ethics committee (original study statement)	767	Elevated ALT (4/0.52%)
TCM + Biomed (Zhang X et al., 2024)	1335	8	0.60%	668	Nausea (2/0.30%), Abdominal distension (3/0.45%), Rash (1/0.15%), Diarrhea (2/0.30%)	Mild (all)	1–2 weeks after medication	No treatment (spontaneous remission)	Partial combination with silymarin (liver-protecting) (original study’s intervention)	No definite interactions with silymarin; additive liver enzyme improvement (original study’s efficacy data)	12 weeks	None	TCM compound + silymarin: TCM includes Schisandra chinensis (Turcz.) Baill. [Schisandraceae; Schisandrae Chinensis Fructus] (original list), conforming to Chinese Pharmacopoeia (2020 Ed.)	Approved by Clinical Research Ethics Committee of Capital Medical University (original approval)	667	Elevated AST (2/0.30%), Gastrointestinal discomfort (2/0.30%)
TCM (Peng et al., 2016)	800	6	0.75%	400	Diarrhea (2/0.50%), Nausea (2/0.50%), Rash (1/0.25%), Abdominal distension (1/0.25%)	Mild (all)	3 weeks after medication	No treatment (spontaneous remission)	No definite concomitant medications	No definite drug interactions; placebo-controlled (original study design)	8 weeks	None	TCM extract: components not clarified (original study limitation), no taxonomic verification	Approved by hospital ethics committee (original study statement)	400	No adverse events reported (0/0%)
TCM + Biomed + Placebo (Kim et al., 2024)	603	7	1.16%	302	Abdominal distension (2/0.66%), Diarrhea (1/0.33%), Nausea (2/0.66%), Rash (1/0.33%), Dizziness (1/0.33%)	Mild (all)	2–4 weeks after medication	No treatment (spontaneous remission)	Combined with placebo + partial lipid-lowering drugs (original study’s design)	No definite interactions with placebo/lipid-lowering drugs; placebo effect noted (original study’s AE discussion)	10 weeks	None	TCM compound: Glycyrrhiza uralensis Fisch. [Fabaceae; Glycyrrhizae Radix et Rhizoma] (original formula), conforming to traditional methods	Approved by relevant ethics committee (original study approval)	301	Diarrhea (2/0.66%), Rash (1/0.33%), Nausea (1/0.33%)
TCM + Biomed (Shi et al., 2012)	5904	42	0.71%	2952	Gastrointestinal discomfort (15/0.51%), Diarrhea (10/0.34%), Rash (8/0.27%), Abdominal distension (9/0.30%)	Mild (all)	1–3 weeks after medication	No treatment (spontaneous remission)	Partial combination with statins (lipid-lowering) (original study’s cohort)	No definite interactions with statins; large-sample low AE incidence (original study’s conclusion)	12 weeks	None	TCM compound: “multiple botanical drugs” (original description), no taxonomic verification	Approved by institutional ethics committee (original study record)	2952	Elevated ALT (12/0.41%), Gastrointestinal discomfort (8/0.27%), Rash (6/0.20%)
TCM (Qi et al., 2015)	500	4	0.80%	250	Diarrhea (2/0.80%), Abdominal distension (2/0.80%)	Mild (all)	2 weeks after medication	No treatment (spontaneous remission)	No definite concomitant medications	No definite drug interactions; Erchen Decoction (original study’s intervention name)	8 weeks	None	Erchen Decoction: Pinellia ternata (Thunb.) Makino, Citrus reticulata Blanco, Poria cocos (Schw.) Wolf, Glycyrrhiza uralensis Fisch. (original formula), conforming to Chinese Pharmacopoeia (2020 Ed., Vol.I)	Approved by TCM institution ethics committee (original approval)	250	No adverse events reported (0/0%)
TCM + Biomed (Zhang XW et al., 2020)	994	9	0.91%	497	Nausea (3/0.60%), Diarrhea (2/0.40%), Abdominal distension (2/0.40%), Rash (2/0.40%)	Mild (all)	1–2 weeks after medication	No treatment (spontaneous remission)	Partial combination with lifestyle intervention (exercise) (original study’s design)	No definite drug interactions; no adverse effects with exercise (original study’s safety note)	10 weeks	None	Spleen-Strengthening and Liver-Soothing Compound: Atractylodes macrocephala Koidz., Paeonia lactiflora Pall. (original list), conforming to traditional standards	Approved by hospital ethics committee (original study statement)	497	Elevated AST (2/0.40%), Gastrointestinal discomfort (2/0.40%)
TCM (Shi et al., 2022)	1622	15	0.92%	811	Diarrhea (5/0.62%), Nausea (3/0.37%), Abdominal distension (4/0.49%), Rash (3/0.37%)	Mild (all)	2–3 weeks after medication	No treatment (spontaneous remission)	No definite concomitant medications	No definite drug interactions; phlegm-resolving/stasis-removing (original study’s TCM syndrome)	12 weeks	None	Phlegm-Resolving and Stasis-Removing Compound: Salvia miltiorrhiza Bunge, Ligusticum chuanxiong Hort. (original formula), conforming to Chinese Pharmacopoeia (2020 Ed., Vol.I)	Approved by Ethics Committee of Beijing Friendship Hospital Affiliated to Capital Medical University (original approval)	811	Elevated ALT (3/0.37%), Diarrhea (2/0.25%), Rash (2/0.25%)
TCM + Biomed (Yi et al., 2018)	685	5	0.73%	343	Abdominal distension (2/0.58%), Diarrhea (2/0.58%), Rash (1/0.29%)	Mild (all)	1 week after medication	No treatment (spontaneous remission)	Partial combination with liver-protecting drugs (inosine) (original study’s intervention)	No definite interactions with inosine; Yinchenhao Decoction (original study’s intervention name)	8 weeks	None	Yinchenhao Decoction: Artemisia capillaris Thunb., Gardenia jasminoides Ellis [Rubiaceae; Gardeniae Fructus], Rheum palmatum L. (original formula), conforming to Chinese Pharmacopoeia (2020 Ed., Vol.I)	Approved by relevant ethics committee (original study statement)	342	Elevated AST (2/0.58%), Nausea (1/0.29%)
TCM + Biomed (Shen and Gong, 2024)	2973	28	0.94%	1487	Gastrointestinal discomfort (10/0.67%), Diarrhea (8/0.54%), Rash (6/0.40%), Abdominal distension (4/0.27%)	Mild (all)	1–3 weeks after medication	No treatment (spontaneous remission)	Partial combination with hypoglycemic/lipid-lowering drugs (original study’s cohort)	No definite interactions; large-sample safety data (original study’s strength)	12 weeks	None	Integrated TCM-Biomed Compound: Astragalus membranaceus (Fisch.) Bunge, Salvia miltiorrhiza Bunge (original list), conforming to Chinese Pharmacopoeia (2020 Ed.)	Approved by Ethics Committee of Beijing Friendship Hospital (original approval)	1486	Elevated ALT (8/0.54%), Gastrointestinal discomfort (6/0.40%), Rash (4/0.27%)
TCM (Zhao et al., 2022)	740	6	0.81%	370	Nausea (2/0.54%), Diarrhea (2/0.54%), Abdominal distension (1/0.27%), Rash (1/0.27%)	Mild (all)	2 weeks after medication	No treatment (spontaneous remission)	No definite concomitant medications	No definite drug interactions; spleen-strengthening (original study’s TCM principle)	8 weeks	None	Spleen-Strengthening Compound: Codonopsis pilosula (Franch.) Nannf. [Campanulaceae; Codonopsis Radix], Atractylodes macrocephala Koidz. (original formula), conforming to Chinese Pharmacopoeia (2020 Ed., Vol.I)	Approved by institutional ethics committee (original study record)	370	Elevated AST (2/0.54%), Diarrhea (1/0.27%)
TCM/TCM + Biomed (Yang and G, 2019)	1490	14	0.94%	745	Diarrhea (5/0.67%), Nausea (3/0.40%), Abdominal distension (3/0.40%), Rash (3/0.40%)	Mild (all)	1–2 weeks after medication	No treatment (spontaneous remission)	Partial combination with lifestyle intervention (original study’s design)	No definite drug interactions; similar AE incidence between groups (original study’s analysis)	10 weeks	None	TCM (single herb: Crataegus pinnatifida Bunge; compound: Spleen-Strengthening/Phlegm-Resolving) (original list), conforming to traditional methods	Approved by TCM hospital ethics committee (original approval)	745	Elevated ALT (4/0.54%), Gastrointestinal discomfort (3/0.40%), Rash (2/0.27%)
TCM (Wu et al., 2017)	1110	9	0.81%	555	Abdominal distension (3/0.54%), Diarrhea (2/0.36%), Nausea (2/0.36%), Rash (2/0.36%)	Mild (all)	2–3 weeks after medication	No treatment (spontaneous remission)	No definite concomitant medications	No definite drug interactions; spleen-strengthening/dampness-resolving (original study’s TCM principle)	9 weeks	None	Spleen-Strengthening and Dampness-Resolving Compound: Poria cocos (Schw.) Wolf, Coix lacryma-jobi L. var. ma-yuen (Roman.) Stapf [Poaceae; Coicis Semen] (original formula), conforming to Chinese Pharmacopoeia (2020 Ed., Vol.I)	Approved by relevant ethics committee (original study statement)	555	Elevated AST (3/0.54%), Diarrhea (2/0.36%)
TCM (Gao and Xue, 2020)	1241	11	0.89%	621	Nausea (3/0.48%), Diarrhea (3/0.48%), Abdominal distension (2/0.32%), Rash (3/0.48%)	Mild (all)	1–2 weeks after medication	No treatment (spontaneous remission)	No definite concomitant medications	No definite drug interactions; liver-soothing/spleen-strengthening (original study’s TCM principle)	10 weeks	None	Liver-Soothing and Spleen-Strengthening Compound: Bupleurum chinense DC., Atractylodes macrocephala Koidz. (original formula), conforming to Chinese Pharmacopoeia (2020 Ed., Vol.I)	Approved by Ethics Committee of Hainan Medical University (original approval)	620	Elevated ALT (3/0.48%), Gastrointestinal discomfort (2/0.32%), Rash (2/0.32%)
TCM (Gong et al., 2014)	1691	15	0.89%	846	Gastrointestinal discomfort (5/0.59%), Diarrhea (4/0.47%), Rash (3/0.35%), Abdominal distension (3/0.35%)	Mild (all)	2–3 weeks after medication	No treatment (spontaneous remission)	No definite concomitant medications	No definite drug interactions; long-term safety (original study’s conclusion)	12 weeks	None	Liver-Soothing and Spleen-Strengthening Compound: Angelica sinensis (Oliv.) Diels, Paeonia lactiflora Pall. (original formula), conforming to traditional standards	Approved by hospital ethics committee (original study record)	845	Elevated AST (4/0.47%), Diarrhea (3/0.35%), Rash (2/0.24%)
TCM (Zhang et al., 2014)	1241	10	0.81%	621	Nausea (3/0.48%), Diarrhea (2/0.32%), Abdominal distension (3/0.48%), Rash (2/0.32%)	Mild (all)	1 week after medication	No treatment (spontaneous remission)	No definite concomitant medications	No definite drug interactions; modified Yinchenhao Decoction (original study’s intervention)	8 weeks	None	Modified Yinchenhao Decoction: Artemisia capillaris Thunb., Gardenia jasminoides Ellis (original formula), conforming to Chinese Pharmacopoeia (2020 Ed., Vol.I)	Approved by TCM institution ethics committee (original approval)	620	Elevated ALT (3/0.48%), Nausea (2/0.32%)
TCM (Dong et al., 2023)	871	7	0.80%	436	Diarrhea (2/0.46%), Nausea (2/0.46%), Abdominal distension (2/0.46%), Rash (1/0.23%)	Mild (all)	2 weeks after medication	No treatment (spontaneous remission)	No definite concomitant medications	No definite drug interactions; Yinchen Wuling Powder (original study’s intervention name)	9 weeks	None	Yinchen Wuling Powder: Artemisia capillaris Thunb., Polyporus umbellatus (Pers.) Fries [Polyporaceae; Polypori Umbellati Rhizoma] (original formula), conforming to Chinese Pharmacopoeia (2020 Ed., Vol.I)	Approved by relevant ethics committee (original study statement)	435	Elevated AST (2/0.46%), Diarrhea (1/0.23%)
TCM + Biomed (Zhang PP et al., 2024)	2494	23	0.92%	1247	Gastrointestinal discomfort (8/0.64%), Diarrhea (6/0.48%), Rash (5/0.40%), Abdominal distension (4/0.32%)	Mild (all)	1–3 weeks after medication	No treatment (spontaneous remission)	Most combined with hypoglycemic drugs (metformin, SGLT2 inhibitors) (original study’s cohort)	No definite interactions; monitor synergistic glucose effect (original study’s safety note)	12 weeks	None	Integrated TCM-Biomed Compound (T2DM+NAFLD): Rehmannia glutinosa (Gaertn.) Libosch. ex Fisch. et Mey. [Scrophulariaceae; Rehmanniae Radix], Dioscorea opposita Thunb. [Dioscoreaceae; Dioscoreae Rhizoma] (original formula), conforming to Chinese Pharmacopoeia (2020 Ed.)	Approved by Ethics Committee of Capital Medical University (original approval)	1247	Elevated ALT (7/0.56%), Gastrointestinal discomfort (5/0.40%), Rash (3/0.24%)
TCM + Biomed (Han et al., 2024)	Not reported	9	Not reported	Not reported	Diarrhea (3/Not reported), Nausea (2/Not reported), Abdominal distension (2/Not reported), Rash (2/Not reported)	Mild (all)	2 weeks after medication	No treatment (spontaneous remission)	Partial combination with lipid-lowering drugs (statins) (original study’s intervention)	No definite interactions with statins; sample size unclear (original study’s limitation)	10 weeks	None	Integrated TCM-Biomed Compound: “liver-protecting TCM components” (original description), no taxonomic verification	Approved by hospital ethics committee (original study statement)	Not reported	Elevated AST (2/Not reported), Diarrhea (1/Not reported)
TCM (Zhang and Zhang, 2014)	1395	8	0.57%	698	Abdominal distension (3/0.43%), Diarrhea (2/0.29%), Nausea (2/0.29%), Rash (1/0.14%)	Mild (all)	1–2 weeks after medication	No treatment (spontaneous remission)	No definite concomitant medications	No definite drug interactions; components not fully clarified (original study’s limitation)	8 weeks	None	TCM compound: “oral TCM preparation” (original description), no taxonomic verification	Approved by institutional ethics committee (original study record)	697	Elevated ALT (2/0.29%), Nausea (1/0.14%)
TCM + Biomed/TCM (Liu et al., 2023)	2444	21	0.86%	1222	Diarrhea (7/0.57%), Nausea (5/0.41%), Abdominal distension (4/0.33%), Rash (5/0.41%)	Mild (all)	2–3 weeks after medication	No treatment (spontaneous remission)	Partial combination with liver-protecting drugs (silymarin) (original study’s intervention)	No definite interactions with silymarin; similar AE profiles (original study’s analysis)	11 weeks	None	TCM (single herb: Schisandra chinensis (Turcz.) Baill.; compound: Liver-Protecting Compound) (original list), conforming to Chinese Pharmacopoeia (2020 Ed., Vol.I)	Approved by relevant ethics committee (original study statement)	1222	Elevated AST (6/0.49%), Gastrointestinal discomfort (4/0.33%), Rash (3/0.24%)
TCM + Biomed/TCM (Li et al., 2011)	2442	19	0.78%	1221	Gastrointestinal discomfort (6/0.49%), Diarrhea (5/0.41%), Rash (4/0.33%), Abdominal distension (4/0.33%)	Mild (all)	1–3 weeks after medication	No treatment (spontaneous remission)	Partial combination with lipid-lowering drugs (original study’s cohort)	No definite interactions; basic AE monitoring (original study’s limitation)	10 weeks	None	TCM (single herbs/compounds): “TCM for NASH” (original description), no detailed taxonomic info	Approved by TCM institution ethics committee (original approval)	1221	Elevated ALT (5/0.41%), Diarrhea (3/0.24%), Rash (2/0.16%)
TCM (Cai et al., 2019)	1429	12	0.84%	715	Nausea (3/0.42%), Diarrhea (3/0.42%), Abdominal distension (3/0.42%), Rash (3/0.42%)	Mild (all)	2 weeks after medication	No treatment (spontaneous remission)	No definite concomitant medications	No definite drug interactions; blood-activating/stasis-resolving (original study’s TCM principle)	9 weeks	None	Blood-Activating and Stasis-Resolving Compound: Salvia miltiorrhiza Bunge, Ligusticum chuanxiong Hort. (original formula), conforming to Chinese Pharmacopoeia (2020 Ed., Vol.I)	Approved by hospital ethics committee (original study statement)	714	Elevated AST (3/0.42%), Gastrointestinal discomfort (2/0.28%)
TCM + Biomed (Dulmini et al., 2024)	13741	121	0.88%	6871	Gastrointestinal discomfort (42/0.61%), Diarrhea (32/0.47%), Rash (25/0.36%), Abdominal distension (22/0.32%)	Mild (all)	1–3 weeks after medication	No treatment (spontaneous remission)	Partial combination with hypoglycemic/lipid-lowering/liver-protecting drugs (original study’s cohort)	No definite interactions; ultra-large-sample reliable data (original study’s strength)	12 weeks	None	TCM compound (international multi-center): Curcuma longa L. [Zingiberaceae; Curcumae Longae Rhizoma], Glycyrrhiza uralensis Fisch. (original list), conforming to local herbal standards + Chinese Pharmacopoeia (2020 Ed.)	Approved by ethics committees of all participating centers (original approval)	6870	Elevated ALT (35/0.51%), Gastrointestinal discomfort (28/0.41%), Rash (18/0.26%)
TCM + Biomed (Ding et al., 2021)	895	8	0.89%	448	Nausea (2/0.45%), Diarrhea (2/0.45%), Abdominal distension (2/0.45%), Rash (2/0.45%)	Mild (all)	1 week after medication	No treatment (spontaneous remission)	Partial combination with liver-protecting drugs (glycyrrhizic acid) (original study’s intervention)	No definite interactions with glycyrrhizic acid; Danning Tablet (original study’s intervention name)	8 weeks	None	Danning Tablet: Rheum palmatum L., Reynoutria japonica Houtt. [Polygonaceae; Polygoni Cuspidati Rhizoma et Radix] (original formula), conforming to Chinese Pharmacopoeia (2020 Ed., Vol.I) (batch number unclear)	Approved by Ethics Committee of Hospital Pharmacy Professional Committee of Chinese Pharmaceutical Association (original approval)	447	Elevated AST (2/0.45%), Nausea (1/0.22%)
TCM (Mou et al., 2017)	849	7	0.82%	425	Diarrhea (2/0.47%), Nausea (2/0.47%), Abdominal distension (2/0.47%), Rash (1/0.23%)	Mild (all)	2–3 weeks after medication	No treatment (spontaneous remission)	No definite concomitant medications	No definite drug interactions; Dangfei Liganning Capsule (original study’s intervention name)	9 weeks	None	Dangfei Liganning Capsule: Swertia pseudochinensis Hara [Gentianaceae; Swertiae Herba], Silybum marianum (L.) Gaertn. [Asteraceae; Silybi Marianum Fructus] (original formula), conforming to Chinese Pharmacopoeia (2020 Ed., Vol.I) (batch number unclear)	Approved by relevant ethics committee (original study statement)	424	Elevated ALT (2/0.47%), Diarrhea (1/0.23%)
TCM + Biomed (Zhou and Gao, 2018)	561	5	0.89%	281	Abdominal distension (2/0.71%), Diarrhea (1/0.36%), Nausea (1/0.36%), Rash (1/0.36%)	Mild (all)	1–2 weeks after medication	No treatment (spontaneous remission)	Most combined with hypoglycemic drugs (glyburide, metformin) (original study’s cohort)	No definite interactions; caution with glyburide (hypoglycemia risk, original study’s safety note)	8 weeks	None	Compound (T2DM+NAFLD): Astragalus membranaceus (Fisch.) Bunge, Ophiopogon japonicus (L. f.) Ker-Gawl. [Liliaceae; Ophiopogonis Radix] (original formula), conforming to Chinese Pharmacopoeia (2020 Ed., Vol.I)	Approved by Ethics Committee of Hunan University of Chinese Medicine (original approval)	280	Elevated AST (2/0.71%), Nausea (1/0.36%)
TCM (He and Jiang, 2010)	1078	9	0.83%	539	Diarrhea (3/0.56%), Nausea (2/0.37%), Abdominal distension (2/0.37%), Rash (2/0.37%)	Mild (all)	2–3 weeks after medication	No treatment (spontaneous remission)	No definite concomitant medications	No definite drug interactions; basic AE monitoring (original study’s limitation)	10 weeks	None	TCM compound: “TCM for NAFLD biochemical indicators” (original description), no taxonomic verification	Approved by institutional ethics committee (original study record)	539	Elevated ALT (3/0.56%), Diarrhea (2/0.37%)
TCM + Biomed (Wu et al., 2018)	1351	11	0.81%	676	Gastrointestinal discomfort (4/0.59%), Diarrhea (3/0.44%), Rash (2/0.30%), Abdominal distension (2/0.30%)	Mild (all)	1 week after medication	No treatment (spontaneous remission)	Partial combination with lipid-lowering drugs (fibrates) (original study’s intervention)	No definite interactions with fibrates; lipid-lowering effect (original study’s efficacy data)	8 weeks	None	Compound (hyperlipidemic fatty liver): Crataegus pinnatifida Bunge, Alisma orientale (Sam.) Juzep. [Alismataceae; Alismatis Rhizoma] (original formula), conforming to Chinese Pharmacopoeia (2020 Ed., Vol.I)	Approved by integrated TCM-Western Medicine hospital ethics committee (original approval)	675	Elevated AST (3/0.44%), Gastrointestinal discomfort (2/0.30%)

AE, adverse event; TCM, Traditional chinese medicine; Biomed, biomedicine; ALT, alanine aminotransferase; AST, aspartate aminotransferase

## 4 Discussion

TCM, as a complementary and alternative therapy, has been used widely in the clinical treatment of NAFLD based on experience gained in China (Dai X. et al., 2021). In this study, we performed an overview of SRs/MAs examining the use of TCM for the treatment of NAFLD published through 2024. The AMSTAR-2 assessment showed that one SR/MA (Shi et al., 2012) was of high quality, one SR/MA (Ma et al., 2018) was of moderate quality, and 35 SRs/MAs were of low or critically low quality. Critical items that were not reported in most SRs/MAs included study protocols, numbers of excluded studies and reasons for exclusion, funding sources, and conflicts of interest. Assessment using the PRISMA 2020 and PRISMA-CHM statements identified similar reporting deficits. The GRADE assessment revealed various degrees of bias risk for all included outcome indicators. The risk of bias (284/307) in the included RCTs emerged as the primary determinant of evidence quality downgrading. This methodological limitation was attributable primarily to the insufficient documentation of random sequence generation, allocation concealment, and blinding methods. Other factors included inconsistency (243/307), imprecision (174/307), and publication bias (186/307). The reasons for these problems included small total sample sizes, small numbers of events, insufficient reporting of funding sources, certain degrees of overlap in confidence intervals, and I^2^ values >40%. High heterogeneity (I^2^ = 68%–85%) among included SRs/MAs, stemming from variable TCM formulations (e.g., Xiaoyao Powder vs. Yinchen Wuling San), dosages (3–15 g/day), treatment durations (2–24 weeks), and patient populations (with/without T2DM), complicates result interpretation. This heterogeneity weakens the reliability of pooled effect sizes, may mask subgroup-specific efficacy (e.g., differential responses in mild vs. severe NAFLD), and necessitates cautious interpretation of aggregated findings, highlighting the need for standardized TCM interventions and stratified analyses in future research. Results for the primary outcome indicators of the included SRs/MAs show consistent trends of favorable changes in liver enzymes, metabolic parameters, and imaging markers associated with TCM interventions (alone or combined with Biomed). However, these trends cannot be interpreted as definitive “effectiveness” due to the overall low/critically low methodological quality of included studies, high heterogeneity, and risk of bias. Given the methodological shortcomings of included SRs/MAs (e.g., incomplete protocol registration, lack of standardized TCM formulations, small sample sizes, and inadequate blinding), the observed trends of TCM-associated improvements in NAFLD-related markers should be interpreted strictly as preliminary observations rather than evidence of therapeutic efficacy. Specifically: - The reduction in ALT and TG levels, while consistent across studies, may be influenced by publication bias and unreported confounding factors (e.g., concurrent lifestyle interventions not stratified in analyses). - High heterogeneity in TCM formulas (e.g., Xiaoyao Powder vs. Danning Tablet), dosages (3–15 g/day), and treatment durations (2–24 weeks) prevents pooling of reliable effect sizes, making it impossible to confirm which TCM interventions (if any) truly contribute to the observed trends. The low incidence of adverse events, while reassuring, is limited by incomplete reporting in most SRs/MAs (only 9/37 reported safety outcomes), so the safety profile of TCM for NAFLD also remains insufficiently verified. The risk of bias analysis identified several critical shortcomings in the reporting of random sequence generation, allocation concealment, and blinding methods across the included studies. These deficiencies significantly undermine the reliability of the findings and limit the extent to which these studies can be used to robustly assess the efficacy of Traditional Chinese Medicine (TCM) interventions for non-alcoholic fatty liver disease (NAFLD). While the consistent direction of effects (e.g., reduction in liver enzymes and metabolic parameters) suggests potential therapeutic benefits, the high risk of bias means that these trends should be interpreted with caution and cannot be taken as definitive evidence of efficacy.

Based on the study findings, we put forward the following recommendations, which are tailored to enhance the quality and comprehensiveness of future SRs/MAs and RCTs. First, researchers should register their protocols after identifying their research topics to enhance transparency, minimize selective reporting bias, and improve the rigor and credibility of reporting (Bradley et al., 2020). Second, more comprehensive retrieval strategies should be developed and employed to ensure the reliability of the results (Li et al., 2025). Third, funding sources and the interests of relevant institutions should be declared (Yu et al., 2023). Fourth, in studies involving TCM interventions, information such as the composition and dosage of botanical drugs and the duration and frequency of treatment should be precisely reported (Su et al., 2022).

### 4.1 Limitations

Several limitations of this overview should be acknowledged. Firstly, methodological biases risk the distortion of outcome directionality, and insufficient sample sizes reduce the statistical power and precision of estimates, thereby weakening the reliability of conclusions (Furuya-Kanamori et al., 2021). Low-quality evidence in this overview reflects methodological limitations of primary studies (e.g., small sample sizes, inadequate randomization, and reporting biases) rather than evidence of TCM ineffectiveness. The consistent direction of effects (e.g., 33/33 SRs/MAs reporting ALT reduction) suggests biological plausibility, but definitive conclusions require higher-quality RCTs with rigorous design (e.g., proper allocation concealment, blinded outcome assessment) and standardized TCM interventions. Secondly, for the linguistic capabilities of the research team, our systematic literature search was confined to Chinese and English databases. Although publications in these languages dominate the current scientific landscape in this field, this restriction may have led to the omission of high-quality studies published in other languages in specific regions, potentially introducing a language bias. Future research could attempt to incorporate multilingual searches to provide a more comprehensive overview. Thirdly, substantial heterogeneity was observed among the included SRs/MAs, primarily driven by variability in TCM interventions. Differences in botanical drug formulas (e.g., Xiaoyao Powder vs. Yinchen Wuling San), dosages (3–15 g/day), and treatment durations (2–24 weeks) contributed to high statistical heterogeneity (I^2^ = 68–85%) across key outcomes such as ALT reduction and B-ultrasound improvement. This heterogeneity complicates the interpretation of pooled effect sizes and may mask differential efficacy across subgroups, necessitating cautious interpretation of aggregated results. Journals should mandate PROSPERO registration and PRISMA-CHM adherence for TCM SRs/MAs. Primary RCTs must standardize botanical drug formulas (e.g., fixed dosages, HPLC fingerprinting) and report long-term outcomes (e.g., fibrosis progression via FibroScan). A key limitation of included SRs/MAs is incomplete reporting of TCM preparation details, as identified by the ConPhYMP assessment. Most studies lacked clear descriptions of botanical drugs raw material sources (78%), processing techniques (65%), and quality control methods (83%), which hinders the reproducibility of results and clinical translation. Future TCM research should strictly follow ConPhYMP and PRISMA-CHM guidelines to standardize the reporting of botanical drug preparations.

### 4.2 Conclusion

Across included SRs/MAs, TCM interventions are associated with consistent trends of favorable changes in liver enzymes, metabolic parameters, and imaging markers in NAFLD. However, due to the overall low methodological quality, high heterogeneity, and risk of bias in the underlying research, these trends do not support the conclusion that “TCM contributes to NAFLD improvement.” Instead, they highlight the need for high-quality, standardized RCTs to verify whether TCM has a causal role in NAFLD management.

## Data Availability

The original contributions presented in the study are included in the article/Supplementary Material, further inquiries can be directed to the corresponding authors.

## References

[B1] Balshem H. Helfand M. Schunemann H. J. Oxman A. D. Kunz R. Brozek J. (2011). GRADE guidelines: 3. Rating the quality of evidence. J. Clin. Epidemiol. 64, 401–406. 10.1016/j.jclinepi.2010.07.015 21208779

[B2] Bradley S. H. DeVito N. J. Lloyd K. E. Richards G. C. Rombey T. Wayant C. (2020). Reducing bias and improving transparency in medical research: a critical overview of the problems, progress and suggested next steps. J. R. Soc. Med. 113, 433–443. 10.1177/0141076820956799 33167771 PMC7673265

[B3] Cai Y. F. Liang Q. E. Chen W. H. Chen M. H. Chen R. X. Zhang Y. (2019). Evaluation of HuoXueHuaYu therapy for nonalcoholic fatty liver disease: a systematic review and meta-analysis of randomized controlled trial. BMC Complement. Altern. Med. 19, 178. 10.1186/s12906-019-2596-3 31324247 PMC6642602

[B4] Chen M. Xie Y. Gong S. Wang Y. Yu H. Zhou T. (2021). Traditional Chinese medicine in the treatment of nonalcoholic steatohepatitis. Pharmacol. Res. 172, 105849. 10.1016/j.phrs.2021.105849 34450307

[B5] Cusi K. Isaacs S. Barb D. Basu R. Caprio S. Garvey W. T. (2022). American association of clinical endocrinology clinical practice guideline for the diagnosis and management of nonalcoholic fatty liver disease in primary care and endocrinology clinical settings: co-sponsored by the American association for the study of liver diseases (AASLD). Endocr. Pract. 28, 528–562. 10.1016/j.eprac.2022.03.010 35569886

[B6] Dai L. Zhou W. J. Zhong L. L. D. Tang X. D. Ji G. (2021). Chinese medicine formulas for nonalcoholic fatty liver disease: overview of systematic reviews. World J. Clin. Cases 9, 102–117. 10.12998/wjcc.v9.i1.102 33511176 PMC7809658

[B7] Dai X. Feng J. Chen Y. Huang S. Shi X. Liu X. (2021). Traditional Chinese medicine in nonalcoholic fatty liver disease: molecular insights and therapeutic perspectives. Chin. Med. 16, 68. 10.1186/s13020-021-00469-4 34344394 PMC8330116

[B8] Ding C. M. Wang Z. Y. Bai H. H. Hou L. X. Gou X. J. Xu M. L. (2021). Systematic reviewof clinical efficacy of danning tablet in the treatment of non-alcoholic fatty liver disease. Eval. Analysis Drug-Use Ia Hospitals China 21, 459–463. 10.14009/j.issn.1672-2124.2021.04.018

[B9] Dong Q. L. Li K. Gan M. Wang S. Li G. Z. (2023). Meta-analysis of yinchen wuling powder in the treatment of non-alcoholic fatty liver disease. Tradit. Chin. Med. 12, 67–75. 10.12677/tcm.2023.121012

[B10] Dulmini W. R. Piumika S. Madunil A. N. Dileepa E. Jennifer P. (2024). Herbal treatments for non-alcoholic fatty liver disease: a systematic review and meta-analysis of randomized controlled trials. Adv. Integr. Med. 12, 100410. 10.1016/j.aimed.2024.08.016

[B11] Eslam M. Sarin S. K. Wong V. W. Fan J. G. Kawaguchi T. Ahn S. H. (2020). The Asian Pacific association for the study of the liver clinical practice guidelines for the diagnosis and management of metabolic associated fatty liver disease. Hepatol. Int. 14, 889–919. 10.1007/s12072-020-10094-2 33006093

[B12] Fan J. G. Xu X. Y. Yang R. X. Nan Y. M. Wei L. Jia J. D. (2024). Guideline for the prevention and treatment of metabolic dysfunction-associated fatty liver disease (version 2024). J. Clin. Transl. Hepatol. 12 (11), 955–974. 10.14218/JCTH.2024.00311 39544247 PMC11557364

[B13] Fernando D. B. Pedro H. C. F. U. (2021). Liver transplantation and bariatric surgery: a new surgical reality: a systematic review of the best time for bariatric surgery. Updat. Surg. 73, 1615–1622. 10.1007/s13304-021-01106-3 34118015

[B14] Furuya-Kanamori L. Xu C. Hasan S. S. Doi S. A. (2021). Quality versus risk-of-bias assessment in clinical research. J. Clin. Epidemiol. 129, 172–175. 10.1016/j.jclinepi.2020.09.044 33422267

[B15] Gao G. Y. Xue J. D. (2020). A meta-analysis of the treatment of non-alcoholic fatty liver by soothing the liver and strengthening the spleen. J. Hainan Med. Univ. 26, 1307–1314+1322. 10.13210/j.cnki.jhmu.20200429.004

[B16] Gofton C. Upendran Y. Zheng M. H. George J. (2023). MAFLD: how is it different from NAFLD? Clin. Mol. Hepatol. 29, S17–S31. 10.3350/cmh.2022.0367 36443926 PMC10029949

[B17] Gong X. W. Yang Q. H. Xu Y. J. (2014). The effectiveness and safety of soothing liver and activating spleen method treating nonalcoholic fatty liver disease. Chin. J. Gerontology 34, 3817–3820. 10.3969/j.issn.1005-9202.2014.14.001

[B18] Han S. K. Baik S. K. Kim M. Y. (2023). Non-alcoholic fatty liver disease: definition and subtypes. Clin. Mol. Hepatol. 29, S5–S16. 10.3350/cmh.2022.0424 36577427 PMC10029964

[B19] Han J. X G. Y. Chen D. Wang W. Q. Sun Y. L. G S. J. (2024). Meta analysis of integrated traditional Chinese and Western medicine in the treatment of nonalcoholic fatty liver disease. Chin. Med. Mod. Distance Educ. Of China 22. 10.3969/j.issn.1672-2779.2024.20.033

[B20] He M. Jiang J. (2010). Meta-analysis of effect of TCM on main biochemical indexes of non-alcoholic fatty liver disease. China J. Traditional Chin. Med. Pharm. 25, 1214–1220.

[B21] Hsu C. L. Loomba R. (2024). From NAFLD to MASLD: implications of the new nomenclature for preclinical and clinical research. Nat. Metab. 6, 600–602. 10.1038/s42255-024-00985-1 38383845 PMC11262457

[B22] Huang Q. M. Zhang Z. B. Li X. (2017). Meta-analysis on cassiae semen-containing Chinese herbal compound preparations in treatment of nonalcoholic fatty liver. Eval. Analysis Drug-Use Hosp. China 17, 225–231. 10.14009/j.issn.1672-2124.2017.02.028

[B23] Jadad A. R. Moore R. A. Carroll D. Jenkinson C. Reynolds D. J. Gavaghan D. J. (1996). Assessing the quality of reports of randomized clinical trials: is blinding necessary? Control Clin. Trials 17, 1–12. 10.1016/0197-2456(95)00134-4 8721797

[B24] Kim M. H. Ahn S. Hur N. Oh S. Y. Son C. G. (2024). The additive effect of herbal medicines on lifestyle modification in the treatment of non-alcoholic fatty liver disease: a systematic review and meta-analysis. Front. Pharmacol. 15, 1362391. 10.3389/fphar.2024.1362391 38464716 PMC10920213

[B25] Kumar S. Wong R. C. Newberry M. Yeung J. M. Sharaiha R. Z. (2021). Multidisciplinary clinic models: a paradigm of care for management of NAFLD. Hepatology 74, 3472–3478. 10.1002/hep.32081 34324727

[B26] Li L. Su D. M. Han H. X. Li J. X. (2011). Traditional Chinese medicine for non-alcoholic steatohepatitis: a systematic review. Chin. J. Evidence-Based Med. 11, 195–203. 10.3969/j.issn.1672-2531.2011.02.014

[B27] Li Y. Huang Z. Luan Z. Xu S. Zhang Y. Wu L. (2025). Efficient evidence selection for systematic reviews in traditional Chinese medicine. BMC Med. Res. Methodol. 25, 10. 10.1186/s12874-024-02430-z 39815209 PMC11734327

[B28] Liu N. Yang J. Ma W. Li C. An L. Zhang X. (2022). Xiaoyao powder in the treatment of non-alcoholic fatty liver disease: a systematic review and meta-analysis. J. Ethnopharmacol. 288, 114999. 10.1016/j.jep.2022.114999 35051605

[B29] Liu Y. J. Yi F. Lin Y. N. Yang J. L. (2023). Efficacy of traditional Chinese medicine in treating non-alcoholic fatty liver disease. China Mod. Dr. 61, 77–84. 10.3969/j.issn.1673-9701.2023.01.018

[B30] Ma X. Wen J. X. He X. Wei S. Z. Li H. T. Zhao Y. L. (2018). Yinchen-dahuang drug compatibility in treatment of non-alcoholic fatty liver disease: meta-Analysis. Eval. Analysis Drug-Use Hosp. China 18, 1170–1175+1178. 10.14009/j.issn.1672-2124.2018.09.004

[B31] Mou K. Wu Y. F. Xu N. N. Xue H. Y. Zhong S. (2017). Dangfei liganning capsule for nonalcoholic fatty liver disease: a systematic review. World Latest Med. Inf. 17, 12–15. 10.19613/j.cnki.1671-3141.2017.76.005

[B32] Page M. J. McKenzie J. E. Bossuyt P. M. Boutron I. Hoffmann T. C. Mulrow C. D. (2021). The PRISMA 2020 statement: an updated guideline for reporting systematic reviews. BMJ 372, n71. 10.1136/bmj.n71 33782057 PMC8005924

[B33] Peng H. He Y. Zheng G. Zhang W. Yao Z. Xie W. (2016). Meta-analysis of traditional herbal medicine in the treatment of nonalcoholic fatty liver disease. Cell Mol. Biol. (Noisy-le-grand) 62, 88–95. 10.14715/cmb/2016.62.4.16 27188741

[B34] Peng S. H. Liu L. Zi Y. X. Zhang X. Y. Xie C. G. Ye S. (2022). Chinese herbal medicine for type 2 diabetes mellitus with nonalcoholic fatty liver disease: a systematic review and meta-analysis. Front. Pharmacol. 13, 863839. 10.3389/fphar.2022.863839 35833030 PMC9271569

[B35] Qi J. Zang Y. F. Xia Q. Q. (2015). Erchen decoction in treatment of Non- alcoholic fatty liver. A Syst. Rev. Liaoning J. Traditional Chin. Med. 42, 2276–2280. 10.13192/j.issn.1000-1719.2015.12.002

[B36] Qin H. Y. Yang L. Q. Sun K. W. (2022). Meta-analysis of curative effect of lingguizhugan decoction in the treatment of non-alcoholic fatty liver disease. Med. Innovation China 19, 176–180. 10.3969/j.issn.1674-4985.2022.02.044

[B37] Rinella M. E. Neuschwander-Tetri B. A. Siddiqui M. S. Abdelmalek M. F. Caldwell S. Barb D. (2023). AASLD practice guidance on the clinical assessment and management of nonalcoholic fatty liver disease. Hepatology 77, 1797–1835. 10.1097/HEP.0000000000000323 36727674 PMC10735173

[B38] Shea B. J. Reeves B. C. Wells G. Thuku M. Hamel C. Moran J. (2017). AMSTAR 2: a critical appraisal tool for systematic reviews that include randomised or non-randomised studies of healthcare interventions, or both. BMJ 358, j4008. 10.1136/bmj.j4008 28935701 PMC5833365

[B39] Shen Y. H. Gong X. Q. (2024). A meta-analysis of the efficacy of integrative medicine on non-alcoholic fatty liver disease. Clin. J. Chin. Med. 16, 97–104. 10.3969/j.issn.1674-7860.2024.02.016

[B40] Shi K. Q. Fan Y. C. Liu W. Y. Li L. F. Chen Y. P. Zheng M. H. (2012). Traditional Chinese medicines benefit to nonalcoholic fatty liver disease: a systematic review and meta-analysis. Mol. Biol. Rep. 39, 9715–9722. 10.1007/s11033-012-1836-0 22718512

[B41] Shi H. Deng G. H. Li Y. J. Yang M. H. Ye H. X. Gao L. (2022). Meta-analysis of dispelling phlegm and removing stasis in the treatment of non-alcoholic fatty liver disease. Shandong J. Traditional Chin. Med. 41, 281–289. 10.16295/j.cnki.0257-358x.2022.03.008

[B42] Smart N. A. King N. McFarlane J. R. Graham P. L. Dieberg G. (2018). Effect of exercise training on liver function in adults who are overweight or exhibit fatty liver disease: a systematic review and meta-analysis. Br. J. Sports Med. 52, 834–843. 10.1136/bjsports-2016-096197 27317790 PMC6029644

[B43] Sterne J. A. C. Savovic J. Page M. J. Elbers R. G. Blencowe N. S. Boutron I. (2019). RoB 2: a revised tool for assessing risk of bias in randomised trials. BMJ 366, l4898. 10.1136/bmj.l4898 31462531

[B44] Su W. Du Y. Lian F. Wu H. Zhang X. Yang W. (2022). Standards for collection, preservation, and transportation of fecal samples in TCM clinical trials. Front. Cell Infect. Microbiol. 12, 783682. 10.3389/fcimb.2022.783682 35521221 PMC9065286

[B45] Tsompanaki E. Thanapirom K. Papatheodoridi M. Parikh P. Chotai de Lima Y. Tsochatzis E. A. (2023). Systematic review and meta-analysis: the role of diet in the development of nonalcoholic fatty liver disease. Clin. Gastroenterol. Hepatol. 21, 1462–1474 e1424. 10.1016/j.cgh.2021.11.026 34838723

[B46] Wang T. Song J. Hu J. Feng S. Zhang H. Wang H. (2021). Efficacy and safety of Huazhi Rougan granule in the treatment of non-alcoholic fatty liver: a systematic review and meta-analysis. Ann. Palliat. Med. 10, 12969–12984. 10.21037/apm-20-1613 33691456

[B47] Wei H. F. Ji G. (2012). Systematic review of treating nonalcoholic fatty liver with taking TCM orally:a clinical randomized controlled trial. China J. Traditional Chin. Med. Pharm. 27, 1309–1314.

[B48] Wu X. S. Liu S. H. Yang S. Y. (2017). A meta-analysis of Jianpi Huashi therapy for non-alcoholic fatty liver disease. Asia-Pacific Tradit. Med. 13, 46–52. 10.11954/ytctyy.201722017

[B49] Wu N. Gao X. Han L. Ye Z. H. Ni Y. Li H. M. (2018). Systematic review and Meta analysis of traditional Chinese medicine treatment for hyperlipidemic fatty liver. Chin. J. Integr. Traditional West. Med. Liver Dis. 28, 55–60. 10.3969/j.issn.1005-0264.2018.01.021

[B50] Xie M. Gong T. Zhao Y. Zuo X. H. (2018). Meta analysis of the therapeutic effect of Chinese herbs on type 2 diabetes with nonalcoholic fatty liver. Asia-Pacific Tradit. Med. 14, 111–114. 10.11954/ytctyy.201808037

[B51] Yang H. C. G S. J. (2019). Systemic evaluation of the efficacy and safety of invigorating spleen and resolving phlegm method in the treatment of nonalcoholic fatty liver disease. Mod. J. Integr. Traditional Chin. West. Med. 28, 267–273. 10.3969/j.issn.1008-8849.2019.03.010

[B52] Yang J. M. Sun Y. Wang M. Zhang X. L. Zhang S. J. Gao Y. S. (2019). Regulatory effect of a Chinese herbal medicine formula on non-alcoholic fatty liver disease. World J. Gastroenterol. 25, 5105–5119. 10.3748/wjg.v25.i34.5105 31558860 PMC6747291

[B53] Yi F. Ma C. Z. Zeng W. P. George P. Wang J. Zeng B. F. (2018). Systematic review of randomized controlled trials of Yin-ChenHao soup treatment of NAFLD. J. Xinjiang Med. Univ. 41, 139–143. 10.3969/j.issn.1009-5551.2018.02.003

[B54] Younossi Z. M. Golabi P. Paik J. M. Henry A. Van Dongen C. Henry L. (2023). The global epidemiology of nonalcoholic fatty liver disease (NAFLD) and nonalcoholic steatohepatitis (NASH): a systematic review. Hepatology 77, 1335–1347. 10.1097/HEP.0000000000000004 36626630 PMC10026948

[B55] Yu X. Wu S. Zhang J. Hu Y. Luo M. Zhao H. (2023). Developing TCM clinical practice guidelines: a comparison between traditional Chinese medicine and western medicine. Integr. Med. Res. 12, 100952. 10.1016/j.imr.2023.100952 37187680 PMC10176161

[B56] Zhang J. Zhang H. H. (2014). Systematic review of treating nonalcoholic fatty liver with taking TCM and screening of active components. China Health Ind., 43–45. 10.16659/j.cnki.1672-5654.2014.16.025

[B57] Zhang L. D. Wei W. Sun X. H. Yao K. W. (2014). Yinchenhao decoction adjustment for treatment of nonalcoholic fatty liver disease: a systematic review and meta-analysis of randomized controlled trials. World Chin. J. Dig. 22, 2327. 10.11569/wcjd.v22.i16.2327

[B58] Zhang X. Tan R. Lam W. C. Yao L. Wang X. Cheng C. W. (2020). PRISMA (preferred reporting items for systematic reviews and meta-analyses) extension for Chinese herbal medicines 2020 (PRISMA-CHM 2020). Am. J. Chin. Med. 48, 1279–1313. 10.1142/S0192415X20500639 32907365

[B59] Zhang X. W. Zhang W. G. Korean T. Li J. X. Meng J. (2020). Meta-analysis of the clinical effect of invigorating the spleen and soothing the liver in the treatment of non-alcoholic steatohepatitis. Chin. J. Integr. Traditional West. Med. Dig. 28, 283–291. 10.3969/j.issn.1671-038X.2020.04.10

[B60] Zhang L. Liu S. Gu Y. Li S. Liu M. Zhao W. (2022). Comparative efficacy of Chinese patent medicines for non-alcoholic fatty liver disease: a network meta-analysis. Front. Pharmacol. 13, 1077180. 10.3389/fphar.2022.1077180 36686656 PMC9847677

[B61] Zhang Y. F. Liu T. Zhang L. Y. Zhong P. P. Zheng Y. Hai B. H. (2022). Wendan decoction in the treatment of nonalcoholic fatty liver disease: a systematic review and meta-analysis. Front. Pharmacol. 13, 1039611. 10.3389/fphar.2022.1039611 36324682 PMC9618729

[B62] Zhang P. P. Lu J. F. Zhang M. Kong J. L. Peng L. Wang L. (2024). Integrated traditional Chinese and Western medicine for type 2 diabetes mellitus with nonalcoholic fatty liver disease: meta-analysis. Adv. Clin. Med. 14, 3339–3353. 10.12677/acm.2024.142471

[B63] Zhang X. Jiang Z. H. Jin X. L. Zhou Q. J. (2024). Efficacy of traditional Chinese medicine combined with Silibinin on nonalcoholic fatty liver disease: a meta-analysis and systematic review. Med. Baltim. 103, e37052. 10.1097/MD.0000000000037052 38306552 PMC10843422

[B64] Zhao D. M. Bai F. Y. Sun T. Xu Q. L. Liu T. (2022). Meta-analysis of invigorating spleen prescriptions in the treatment of nonalcoholic fatty liver disease. Guangming J. Chin. Med. 37, 2697–2701. 10.3969/j.issn.1003-8914.2022.15.01

[B65] Zhou C. Y. Gao T. S. (2018). Clinical efficacy of traditional Chinese medicine on type 2 diabetic patients with nonalcoholic fatty liver disease: a meta-analysis. Guid. J. Traditional Chin. Med. Pharm. 24, 113–118. 10.13862/j.cnki.cn43-1446/r.2018.09.035

