# Supplementary material for: Traditional Chinese medicine for non-alcoholic fatty liver disease: an overview of systematic reviews with evidence mapping and metabolic outcome assessment
Source: Front Pharmacol. 2025 Dec 10;16:1675793. doi: 10.3389/fphar.2025.1675793 (PMC12728050; doi:10.3389/fphar.2025.1675793)
Supplement: Supplementary file 1 [file Table1.docx]

**Supplementary material 1: Search strategy.**

| **PubMed** | ("systematic reviews"(MeSH Terms) OR "Meta-Analyses"(Title/Abstract)) AND ("metabolic related fatty liver disease"(Title/Abstract) OR "Fatty Liver"(Title/Abstract) OR "Non-alcoholic Fatty Liver Disease"(Title/Abstract) OR "fatty liver alcoholic"(Title/Abstract) OR ("Fatty Liver"(MeSH Terms) OR "Non-alcoholic Fatty Liver Disease"(MeSH Terms) OR "fatty liver, alcoholic"(MeSH Terms))) |
| --- | --- |
| **Web of Science** | (((TS=(Metabolic related fatty liver disease or Non-alcoholic Fatty Liver Disease or Fatty liver alcoholic or Fatty Liver or Fatty liver, alcoholi)) OR TI=((Metabolic related fatty liver disease or Non-alcoholic Fatty Liver Disease or Fatty liver alcoholic or Fatty Liver or Fatty liver, alcoholi))) OR AB=((Metabolic related fatty liver disease or Non-alcoholic Fatty Liver Disease or Fatty liver alcoholic or Fatty Liver or Fatty liver, alcoholi))) and (((TS=(Medicine, chinese traditional or Chinese herbal medicine or Acupuncture or Herbal or drugs chinese herbal or herbal medicine)) or TI=(Medicine, chinese traditional or Chinese herbal medicine or Acupuncture or Herbal or drugs chinese herbal or herbal medicine)) or AB=(Medicine, chinese traditional or Chinese herbal medicine or Acupuncture or Herbal or drugs chinese herbal or herbal medicine)) and (((TS=(systematic review or meta)) or TI=(systematic review or meta)) or AB=(systematic review or meta)) |
| **Excerpta Medica Database** | #1 'medicine, chinese traditional'/exp  #2 'Fatty Liver'/exp  #3 'systematic reviews'/exp  #4 'medicine, chinese traditional':ab,ti OR 'chinese herbal medicine':ab,ti OR 'Herbal ':ab,ti OR herbal medicine:ab,ti OR 'drugs chinese herbal':ab,ti  #5 ' metabolic related fatty liver disease':ab,ti OR ' Fatty Liver':ab,ti OR ' Non-alcoholic Fatty Liver Disease':ab,ti OR ' fatty liver alcoholic':ab,ti OR ' fatty liver, alcoholic':ab,ti  #6 'systematic reviews':ab,ti OR 'meta':ab,ti  #6 #1 OR #4  #7 #2 OR #5  #8 #3 OR #6  #9 #6 AND #7 AND #8 |
| **The Cochrane Library** | #1 MeSH descriptor: (Fatty Liver) explode all trees  #2 (Metabolic related fatty liver disease or Non-alcoholic Fatty Liver Disease or Fatty liver alcoholic or Fatty Liver or Fatty liver, alcoholi):ti,ab, kw  #3 MeSH descriptor: (Medicine, chinese traditional) explode all trees  #4 (Medicine, chinese traditional or Chinese herbal medicine or Acupuncture or Herbal or drugs chinese herbal or herbal medicine):ti,ab,kw  #5 #1 OR #2  #6 #3 OR #4  #7 #5 AND #6 |
| **China National Knowledge Infrastructure** | (SU=’脂肪肝 + 非酒精性脂肪性肝病 + 代谢相关脂肪性肝病 + 肥气 + 肝痞’ or TI=’脂肪肝 + 非酒精性脂肪性肝病 + 代谢相关脂肪性肝病 + 肥气 + 肝痞’ or KY=’脂肪肝 + 非酒精性脂肪性肝病 + 代谢相关脂肪性肝病 + 肥气 + 肝痞’ or AB=’脂肪肝 + 非酒精性脂肪性肝病 + 代谢相关脂肪性肝病 + 肥气 + 肝痞’) AND (SU=' 系统评价 + meta' or TI=' 系统评价 + meta' or KY=' 系统评价 + meta' or AB=' 系统评价 + meta' ) |
| **Wanfang Database** | (题名或关键词： (("脂肪肝" OR "非酒精性脂肪性肝病" or "代谢相关脂肪性肝病" or "肥气" or "肝痞") and ("中药" or "中医药") and (随机))) or (主题: (("脂肪肝" or "非酒精性脂肪性肝病" or "代谢相关脂肪性肝病" or "肥气" or "肝痞") and ("系统评价" or "meta"))) |
| **VIP Database for Chinese Technical Periodicals** | (M=系统评价 OR R=系统评价 OR M=meta OR R=meta) AND (M=脂肪肝 OR R=脂肪肝 OR M=非酒精性脂肪性肝病 OR R=非酒精性脂肪性肝病 OR M=代谢相关脂肪性肝病 OR R=代谢相关脂肪性肝病 OR M=肥气 OR R=肥气 OR M=肝痞 OR R=肝痞) |
| **China Biology Medicine Database** | (("非酒精性脂肪性肝病"(常用字段) OR "代谢相关脂肪性肝病"(常用字段) OR "脂肪肝"(主题词) OR "肥气"(常用字段) OR "肝痞"(常用字段))) AND (("系统评价"(常用字段) OR "meta"(主题词))) |
| **Scopus Abstract and Citation Database** | ( TITLE-ABS-KEY ( "systematic review*" OR "meta-analy*" ) )  AND  ( TITLE-ABS-KEY (  "metabolic related fatty liver disease" OR  "fatty liver" OR  "non-alcoholic fatty liver disease" OR  "alcoholic fatty liver" OR  "non-alcoholic steatohepatitis" OR  "nafld" OR  "mafld" OR  "afl"  ) ) |

**Supplementary material 2. The assessment of GRADE.**

| **References** | **Intervention** | **comparator** | **Outcome**  **indicators** | **Numberofincludedtrials** | **Riskofbias** | **Indirectness** | **Inconsistency** | **Imprecision** | **Publicationbias** | **Certainty** | **Relative effect** |
| --- | --- | --- | --- | --- | --- | --- | --- | --- | --- | --- | --- |
| **Ma et al. (2018)** | TCM+Biomed | Biomed | Overall efficacy | 12 | -1 | 0 | -1 | -1 | -1 | Very low | 1.24( 1.11, 1.40) |
| **Ma et al. (2018)** | TCM+Biomed | Biomed | ALT | 15 | -1 | 0 | -1 | -1 | -1 | Very low | -9.87 (-13.48, 6.26) |
| **Ma et al. (2018)** | TCM+Biomed | Biomed | AST | 15 | -1 | 0 | -1 | -1 | -1 | Very low | -7.55 (-11-11,-3.96) |
| **Ma et al. (2018)** | TCM+Biomed | Biomed | GGT | 9 | -1 | 0 | -1 | -1 | -1 | Very low | -12.32 (-18.64, -6.00) |
| **Ma et al. (2018)** | TCM+Biomed | Biomed | TC | 13 | -1 | 0 | -1 | -1 | -1 | Very low | -0.53 (-0.89, -0.18) |
| **Ma et al. (2018)** | TCM+Biomed | Biomed | TG | 15 | -1 | 0 | -1 | -1 | -1 | Very low | -0.78 (-1.27, -0.29) |
| **Ma et al. (2018)** | TCM+Biomed | Biomed | HDL-C | 6 | -1 | 0 | -1 | -1 | -1 | Very low | 0.08 (-0.07,-0.23) |
| **Ma et al. (2018)** | TCM+Biomed | Biomed | LDL-C | 8 | -1 | 0 | -1 | -1 | -1 | Very low | -0.40 (-0.59, -0.20) |
| **Qi et al. (2015)** | TCM | Biomed | Overall efficacy | 4 | 0 | 0 | -1 | -1 | -1 | Very low | 5.93 (3.5, 10.07) |
| **Qi et al. (2015)** | TCM | Biomed | TC | 3 | -1 | 0 | 0 | -1 | -1 | Very low | -0.54 (-0.73, -0.36) |
| **Qi et al. (2015)** | TCM | Biomed | TG | 3 | -1 | 0 | 0 | -1 | -1 | Very low | -0.39 (-0.7, -0.07) |
| **Qi et al. (2015)** | TCM | Biomed | GGT | 2 | -1 | 0 | -1 | 0 | -1 | Very low | -23.18 (-25.41, -20.95) |
| **Qi et al. (2015)** | TCM | Biomed | ALT | 2 | -1 | 0 | 0 | 0 | -1 | Low | -4.41 (-6.38, -2.43) |
| **Huang et al. (2017)** | TCM+Biomed | Biomed | Overall efficacy | 20 | -1 | 0 | 0 | -1 | -1 | Very low | 1.24 (1.18, 1.31) |
| **Huang et al. (2017)** | TCM+Biomed | Biomed | ALT | 17 | 0 | 0 | -1 | -1 | -1 | Very low | -14.12 (-18.91, -9.33) |
| **Huang et al. (2017)** | TCM+Biomed | Biomed | AST | 16 | 0 | 0 | -1 | -1 | -1 | Very low | -13.67 (-17,-10.35) |
| **Huang et al. (2017)** | TCM+Biomed | Biomed | TC | 19 | 0 | 0 | -1 | -1 | -1 | Very low | -0.83 (-1.23, -0.43) |
| **Huang et al. (2017)** | TCM+Biomed | Biomed | TG | 19 | 0 | 0 | -1 | -1 | -1 | Very low | -0.43 (-0.6, -0.26) |
| **Zhang et al. (2022)** | TCM | Biomed | Overall efficacy | 19 | 0 | 0 | 0 | -1 | -1 | Low | 3.52 (2.33, 5.41) |
| **Zhang et al. (2022)** | TCM | Biomed | BMI | 3 | -1 | 0 | -1 | -1 | -1 | Very low | -0.88 (1.52, -0.23) |
| **Zhang et al. (2022)** | TCM | Biomed | Effective improvement rate of B-ultrasound | 2 | -1 | 0 | 0 | -1 | -1 | Very low | 2.65 (1, 7.03) |
| **Zhang et al. (2022)** | TCM | Biomed | ALT | 5 | -1 | 0 | 0 | -1 | -1 | Very low | -11.44 (14.35, -8.54) |
| **Zhang et al. (2022)** | TCM | Biomed | AST | 4 | -1 | 0 | 0 | -1 | -1 | Very low | 12.72 (-15.58, -9.87) |
| **Zhang et al. (2022)** | TCM | Biomed | TG | 5 | -1 | 0 | 0 | -1 | -1 | Very low | -0.41 (-0.55, -0.27) |
| **Zhang et al. (2022)** | TCM | Biomed | TC | 3 | -1 | 0 | 0 | -1 | -1 | Very low | -1.09 (-1.25, -0.93) |
| **Zhang et al. (2022)** | TCM | Biomed | GGT | 2 | -1 | 0 | 0 | -1 | -1 | Very low | -26.01 (-31.07, -20.06) |
| **Shi et al. (2022)** | TCM | Biomed | Overall efficacy | 17 | -1 | 0 | -1 | -1 | -1 | Very low | 2.87 (2.21, 3.73) |
| **Shi et al. (2022)** | TCM | Biomed | ALT | 14 | -1 | 0 | -1 | -1 | -1 | Very low | 13.62 (-18.35, -8.89) |
| **Shi et al. (2022)** | TCM | Biomed | AST | 11 | -1 | 0 | -1 | -1 | -1 | Very low | 15.4 (-20.48, -10.36) |
| **Shi et al. (2022)** | TCM | Biomed | GGT | 3 | -1 | 0 | -1 | -1 | -1 | Very low | -17.69 (-36.80, 1.43) |
| **Shi et al. (2022)** | TCM | Biomed | TBiL | 2 | -1 | 0 | -1 | -1 | -1 | Very low | 11.07 (-19.20, -2.94) |
| **Shi et al. (2022)** | TCM | Biomed | TG | 14 | -1 | 0 | -1 | -1 | -1 | Very low | -0.53 (-0.72, -0.34) |
| **Shi et al. (2022)** | TCM | Biomed | TC | 12 | -1 | 0 | -1 | -1 | -1 | Very low | -0.61 (-0.79, -0.43) |
| **Shi et al. (2022)** | TCM | Biomed | HDL-C | 5 | -1 | 0 | -1 | -1 | -1 | Very low | 0.19 (0.09, 0.29) |
| **Shi et al. (2022)** | TCM | Biomed | LDL-C | 4 | -1 | 0 | -1 | -1 | -1 | Very low | -0.31 (-0.60, -0.02) |
| **Shi et al. (2022)** | TCM | Biomed | Liver/spleen computed tomography ratio | 3 | -1 | 0 | -1 | -1 | -1 | Very low | 0.14 (0.06, 0.23) |
| **Yi et al. (2018)** | TCM | Biomed | Overall efficacy | 3 | -1 | 0 | 0 | -1 | -1 | Very low | 6.49 (3.27, 12.9) |
| **Yi et al. (2018)** | TCM+Biomed | Biomed | Overall efficacy | 2 | -1 | 0 | 0 | -1 | -1 | Very low | 5.4 (2.27, 12.87) |
| **Shen et al. (2024)** | TCM+Biomed | Biomed | Overall efficacy | 34 | -1 | 0 | 0 | -1 | 0 | Low | 1.23 (1.19, 1.27) |
| **Shen et al. (2024)** | TCM+Biomed | Biomed | AST | 30 | -1 | 0 | -1 | -1 | 0 | Very low | -13.17 (-15.29, -11.05) |
| **Shen et al. (2024)** | TCM+Biomed | Biomed | ALT | 29 | -1 | 0 | -1 | -1 | 0 | Very low | -11.22 (-12.80, -9.64) |
| **Shen et al. (2024)** | TCM+Biomed | Biomed | TC | 23 | -1 | 0 | -1 | -1 | 0 | Very low | -0.72 (-0.86, -0.58) |
| **Shen et al. (2024)** | TCM+Biomed | Biomed | TG | 22 | -1 | 0 | -1 | -1 | 0 | Very low | 0.96 (-1.13, -0.8) |
| **Shen et al. (2024)** | TCM+Biomed | Biomed | BMI | 3 | -1 | 0 | -1 | -1 | 0 | Very low | -3.13 (-8.06, 1.81) |
| **Xie et al. (2018)** | TCM+Biomed | Biomed | Overall efficacy | 10 | -1 | 0 | -1 | 0 | -1 | Very low | 1.51, (1.31, 1.73) |
| **Xie et al. (2018)** | TCM+Biomed | Biomed | FPG | 9 | -1 | 0 | -1 | 0 | -1 | Very low | -0.05,(-0.36, 0.27) |
| **Xie et al. (2018)** | TCM+Biomed | Biomed | 2hpg | 5 | -1 | 0 | 0 | 0 | -1 | Very low | -0.35,(-0.62, -0.08) |
| **Xie et al. (2018)** | TCM+Biomed | Biomed | HOMA-IR | 5 | -1 | 0 | -1 | 0 | -1 | Very low | -0.84,(-1.23, -0.45) |
| **Xie et al. (2018)** | TCM+Biomed | Biomed | ALT | 10 | -1 | 0 | -1 | 0 | -1 | Very low | -1.17,(-1.70, -0.64) |
| **Xie et al. (2018)** | TCM+Biomed | Biomed | AST | 10 | -1 | 0 | -1 | 0 | -1 | Very low | -1.06,(-1.55, -0.56) |
| **Xie et al. (2018)** | TCM+Biomed | Biomed | TC | 10 | -1 | 0 | -1 | 0 | -1 | Very low | -0.48,(-1.09, 0.13) |
| **Xie et al. (2018)** | TCM+Biomed | Biomed | TG | 9 | -1 | 0 | -1 | 0 | -1 | Very low | -0.42,(-0.86, 0.02) |
| **Xie et al. (2018)** | TCM+Biomed | Biomed | BMI | 5 | -1 | 0 | 0 | 0 | -1 | Low | -0.61,(-0.80, -0.41) |
| **Wei et al. (2012)** | TCM | Biomed | Overall efficacy | 8 | -1 | 0 | -1 | 0 | -1 | Very low | 1.05 (0.9, 1.23) |
| **Wei et al. (2012)** | TCM | Placebo | Overall efficacy | 1 | -1 | 0 | -1 | 0 | -1 | Very low | 2.95 (1.17, 5.08) |
| **Wei et al. (2012)** | TCM | Biomed | TG | 10 | -1 | 0 | -1 | 0 | -1 | Very low | -1.16(-1.82, -0.50) |
| **Wei et al. (2012)** | TCM | Biomed | TC | 10 | -1 | 0 | -1 | 0 | -1 | Very low | -1.20(-1.79, -0.62) |
| **Wei et al. (2012)** | TCM | Biomed | ALT | 11 | -1 | 0 | -1 | -1 | -1 | Very low | -0.67(-1.11, -0.22) |
| **Wei et al. (2012)** | TCM | Biomed | AST | 11 | -1 | 0 | -1 | 0 | -1 | Very low | -0.51 (-0.81 , -0.21) |
| **Wei et al. (2012)** | TCM | Biomed | BMI | 2 | -1 | 0 | -1 | 0 | -1 | Very low | -0.32(-0.62, -0.01) |
| **Wei et al. (2012)** | TCM | Placebo | BMI | 1 | -1 | 0 | -1 | 0 | -1 | Very low | -1.55 (-2.10 , -0.99) |
| **Wei et al. (2012)** | TCM | Biomed | Effective improvement rate of B-ultrasound | 2 | -1 | 0 | 0 | 0 | -1 | Low | -0.12(-0.51, 0.27) |
| **Wei et al. (2012)** | TCM | Biomed | Effective improvement rate of B-ultrasound | 3 | -1 | 0 | 0 | 0 | -1 | Low | 1.26(1.12, 1.42) |
| **Wei et al. (2012)** | TCM | Biomed | Liver/spleen computed tomography ratio | 1 | -1 | 0 | 0 | 0 | -1 | Low | 1.04(0.95, 1.15) |
| **Zhou et al. (2018)** | TCM+Biomed | Biomed | Overall efficacy | 5 | -1 | 0 | -1 | -1 | -1 | Very low | 3.88 (2.39, 6.32) |
| **Zhou et al. (2018)** | TCM+Biomed | Biomed | ALT | 6 | -1 | 0 | -1 | 0 | -1 | Very low | -3.43 (-3.83, -3.03) |
| **Zhou et al. (2018)** | TCM+Biomed | Biomed | AST | 5 | -1 | 0 | -1 | 0 | -1 | Very low | -0.06 (-0.21, 0.99) |
| **Zhou et al. (2018)** | TCM+Biomed | Biomed | GGT | 2 | -1 | 0 | -1 | 0 | -1 | Very low | -0.04 (-0.26, -0.10) |
| **Zhou et al. (2018)** | TCM+Biomed | Biomed | FPG | 4 | -1 | 0 | -1 | 0 | -1 | Very low | -0.83(-1.05, -0.6) |
| **Zhou et al. (2018)** | TCM+Biomed | Biomed | 2hpg | 3 | -1 | 0 | -1 | 0 | -1 | Very low | -2.8 (-3.23, -2.37) |
| **Zhou et al. (2018)** | TCM+Biomed | Biomed | HbA1c | 3 | -1 | 0 | -1 | 0 | -1 | Very low | -0.74(-1.24, -0.47) |
| **Zhou et al. (2018)** | TCM+Biomed | Biomed | TC | 3 | -1 | 0 | -1 | 0 | -1 | Very low | 0.17 (-0.08, 0.42) |
| **Zhou et al. (2018)** | TCM+Biomed | Biomed | TG | 5 | -1 | 0 | -1 | 0 | -1 | Very low | -0.73 (-0.82, -0.63) |
| **Zhou et al. (2018)** | TCM+Biomed | Biomed | LDL-C | 4 | -1 | 0 | -1 | 0 | -1 | Very low | -0.38 (-0.6, -0.16) |
| **Wu et al. (2018)** | TCM+Biomed | Biomed | Overall efficacy | 14 | -1 | 0 | -1 | -1 | -1 | Very low | 1.27(1. 20, 1.34) |
| **Wu et al. (2018)** | TCM | Biomed | TC | 8 | -1 | 0 | -1 | -1 | -1 | Very low | -0.85 (-1.38, -0.32) |
| **Wu et al. (2018)** | TCM+Biomed | Biomed | TC | 6 | -1 | 0 | -1 | -1 | -1 | Very low | － 1.01(-1. 77, -0.25) |
| **Wu et al. (2018)** | TCM | Biomed | ALT | 6 | -1 | 0 | -1 | -1 | -1 | Very low | -0.55(-0.72, -0.38) |
| **Wu et al. (2018)** | TCM+Biomed | Biomed | ALT | 4 | -1 | 0 | -1 | -1 | -1 | Very low | -1.20(-1.45,-0.95) |
| **He et al. (2010)** | TCM | Biomed | ALT | 11 | -1 | 0 | -1 | 0 | -1 | Very low | -9.55(-12.45, -6.65) |
| **He et al. (2010)** | TCM | Biomed | AST | 11 | -1 | 0 | -1 | 0 | -1 | Very low | -9.40 (-12.96, -5.85) |
| **He et al. (2010)** | TCM | Biomed | GGT | 11 | -1 | 0 | -1 | 0 | -1 | Very low | -18.31 (-27.06, -9.56) |
| **He et al. (2010)** | TCM | Biomed | TC | 11 | -1 | 0 | -1 | 0 | -1 | Very low | 1.12 (-1.80, -0.44) |
| **He et al. (2010)** | TCM | Biomed | TG | 11 | -1 | 0 | -1 | 0 | -1 | Very low | 0.39 (-0.64, -0.15) |
| **He et al. (2010)** | TCM | Biomed | HDL-C | 11 | -1 | 0 | -1 | 0 | -1 | Very low | 0.21 (0.14, 0.28) |
| **Peng et al. (2022)** | TCM+Biomed | Biomed | TG | 15 | -1 | 0 | -1 | 0 | -1 | Low | -0.35 (-0.51, -0.19) |
| **Peng et al. (2022)** | TCM+Biomed | Biomed | TC | 17 | -1 | 0 | -1 | 0 | -1 | Low | -0.58 (-0.80, -0.36) |
| **Peng et al. (2022)** | TCM+Biomed | Biomed | LDL-C | 15 | -1 | 0 | -1 | 0 | 0 | Low | -0.37 (-0.47,-0.26) |
| **Peng et al. (2022)** | TCM+Biomed | Biomed | HDL-C | 13 | -1 | 0 | -1 | 0 | 0 | Low | 0.20 (0.10,0.29) |
| **Peng et al. (2022)** | TCM+Biomed | Biomed | ALT | 17 | -1 | 0 | -1 | -1 | 0 | Very low | -4.99 (-6.64,-3.33) |
| **Peng et al. (2022)** | TCM+Biomed | Biomed | AST | 17 | -1 | 0 | -1 | -1 | 0 | Very low | -4.76 (-6.35,-3.16) |
| **Peng et al. (2022)** | TCM+Biomed | Biomed | HOMA-IR | 9 | -1 | 0 | -1 | 0 | 0 | Low | -1.01 (-1.22, -0.79) |
| **Peng et al. (2022)** | TCM+Biomed | Biomed | FPG | 18 | -1 | 0 | -1 | 0 | 0 | Low | -0.87 (-1.13, -0.61) |
| **Peng et al. (2022)** | TCM+Biomed | Biomed | 2hpg | 14 | -1 | 0 | -1 | 0 | 0 | Low | -1.45 (-2.00, -0.91) |
| **Peng et al. (2022)** | TCM+Biomed | Biomed | BMI | 11 | -1 | 0 | -1 | -1 | -1 | Very low | -0.73 (-1.35, -0.12) |
| **Peng et al. (2022)** | TCM+Biomed | Biomed | Overall efficacy | 16 | -1 | 0 | -1 | 0 | -1 | Low | 1.37 (1.29, 1.46) |
| **Wang et al. (2021)** | TCM+Biomed | Biomed | ALT | 6 | -1 | 0 | 0 | 0 | -1 | Low | -10.49 ( -17.09, -3.90) |
| **Wang et al. (2021)** | TCM+Biomed | Biomed | AST | 5 | -1 | 0 | -1 | 0 | -1 | Very low | -9.44 (-14.62, -4.26) |
| **Wang et al. (2021)** | TCM+Biomed | Biomed | TG | 6 | -1 | 0 | -1 | 0 | -1 | Very low | -0.40 (-0.56, -0.24) |
| **Wang et al. (2021)** | TCM+Biomed | Biomed | TC | 6 | -1 | 0 | -1 | 0 | -1 | Very low | -0.77 (-0.94, -0.60) |
| **Wang et al. (2021)** | TCM+Biomed | Biomed | HDL-C | 2 | -1 | 0 | 0 | 0 | -1 | Low | 0.38 ( -0.33, 1.09) |
| **Wang et al. (2021)** | TCM+Biomed | Biomed | LDL-C | 2 | -1 | 0 | -1 | 0 | -1 | Very low | -0.50 (-0.68, -0.31) |
| **Wang et al. (2021)** | TCM+Biomed | Biomed | Overall efficacy | 8 | -1 | 0 | -1 | 0 | -1 | Very low | 1.25 ( 1.16, 1.36) |
| **Zhang et al. (2024)** | TCM+Biomed | Biomed | Overall efficacy | 14 | 0 | 0 | -1 | -1 | 0 | Low | 1.25 ( 1.19, 1.32) |
| **Zhang et al. (2024)** | TCM+Biomed | Biomed | TC | 12 | 0 | 0 | -1 | -1 | 0 | Low | -0.38 ( -0.53, -0.23) |
| **Zhang et al. (2024)** | TCM+Biomed | Biomed | TG | 14 | 0 | 0 | -1 | -1 | 0 | Low | -0.38 ( -0.48, -0.27) |
| **Zhang et al. (2024)** | TCM+Biomed | Biomed | ALT | 14 | 0 | 0 | -1 | -1 | 0 | Low | -9.06 ( -11.25, 6.87) |
| **Zhang et al. (2024)** | TCM+Biomed | Biomed | AST | 14 | 0 | 0 | -1 | -1 | 0 | Low | -9.06 (-11.25, -6.87) |
| **Zhang et al. (2024)** | TCM+Biomed | Biomed | GGT | 7 | 0 | 0 | -1 | -1 | 0 | Low | -11.15( -17.39 , -4.92) |
| **Peng et al. (2016)** | TCM | placebo | Overall efficacy | 4 | -1 | 0 | 0 | 0 | -1 | Low | 4.35 (2.68, 7.06) |
| **Peng et al. (2016)** | TCM | placebo | ALT | 4 | -1 | 0 | -1 | 0 | -1 | Very low | 1.01 (-1.65, 3.67) |
| **Peng et al. (2016)** | TCM | placebo | AST | 4 | -1 | 0 | -1 | 0 | -1 | Very low | 0.31 (-3.04, 3.67) |
| **Peng et al. (2016)** | TCM | placebo | TBiL | 3 | -1 | 0 | -1 | 0 | -1 | Very low | 0.03 (-2.48, 2.55) |
| **Peng et al. (2016)** | TCM | placebo | TG | 7 | -1 | 0 | -1 | 0 | -1 | Very low | 0.01 (-0.19, 0.22) |
| **Peng et al. (2016)** | TCM | placebo | TC | 7 | -1 | 0 | -1 | 0 | -1 | Very low | 0.06 (-0.07, 0.19) |
| **Peng et al. (2016)** | TCM | placebo | LDL-C | 3 | -1 | 0 | -1 | 0 | -1 | Very low | -0.04 (-0.19, 0.12) |
| **Peng et al. (2016)** | TCM | placebo | HDL-C | 3 | -1 | 0 | -1 | 0 | -1 | Very low | 0.04 (-0.01, 0.09) |
| **Peng et al. (2016)** | TCM | placebo | Liver/spleen computed tomography ratio | 2 | -1 | 0 | -1 | 0 | -1 | Very low | 0.00 (-0.06, 0.05), |
| **Kim et al. (2024)** | TCM+Biomed | placebo+Biomed | Effective improvement rate of B-ultrasound | 3 | 0 | 0 | -1 | -1 | 0 | Low | 7.9( 0.7, 95.2) |
| **Kim et al. (2024)** | TCM+Biomed | placebo+Biomed | Liver/spleen computed tomography ratio | 2 | 0 | 0 | -1 | -1 | 0 | Low | 0.22(-0.1, 0.6) |
| **Kim et al. (2024)** | TCM+Biomed | placebo+Biomed | ALT | 7 | 0 | 0 | -1 | 0 | 0 | Medium | -10.4(-23.0, 2.3) |
| **Kim et al. (2024)** | TCM+Biomed | placebo+Biomed | AST | 7 | 0 | 0 | -1 | 0 | 0 | Medium | -7.5 (-13.4, -1.7) |
| **Kim et al. (2024)** | TCM+Biomed | placebo+Biomed | GGT | 3 | 0 | 0 | 0 | 0 | 0 | High | -0.6 (-6.7, 5.5) |
| **Kim et al. (2024)** | TCM+Biomed | placebo+Biomed | TG | 7 | 0 | 0 | -1 | -1 | 0 | Low | 15.7 (-50.3,19.0) |
| **Kim et al. (2024)** | TCM+Biomed | placebo+Biomed | TC | 7 | 0 | 0 | -1 | -1 | 0 | Low | -16.0 (-32.7, 0.7) |
| **Kim et al. (2024)** | TCM+Biomed | placebo+Biomed | BMI | 7 | 0 | 0 | -1 | 0 | 0 | Medium | -0.5 (-1.2, 0.2) |
| **Kim et al. (2024)** | TCM+Biomed | placebo+Biomed | HOMA-IR | 6 | 0 | 0 | -1 | -1 | 0 | Low | -0.6 (-1.5, 0.3) |
| **Zhang et al. (2022)** | TCM+Biomed | Biomed | Overall efficacy | 7 | -1 | 0 | 0 | -1 | 0 | Low | 4.21 (2.63, –6.72) |
| **Zhang et al. (2022)** | TCM+Biomed | Biomed | ALT | 5 | -1 | 0 | -1 | -1 | -1 | Very low | -12.48 (-20.14, -4.8) |
| **Zhang et al. (2022)** | TCM+Biomed | Biomed | AST | 5 | -1 | 0 | -1 | -1 | -1 | Very low | -10.96(-16.31, -5.62) |
| **Zhang et al. (2022)** | TCM+Biomed | Biomed | GGT | 2 | -1 | 0 | -1 | -1 | -1 | Very low | -9.59 (-30.75, 11.56) |
| **Zhang et al. (2022)** | TCM+Biomed | Biomed | TC | 6 | -1 | 0 | -1 | -1 | -1 | Very low | -0.61 (-1.17, -0.05) |
| **Zhang et al. (2022)** | TCM+Biomed | Biomed | TG | 6 | -1 | 0 | -1 | -1 | -1 | Very low | -0.56 (-0.71, -0.40) |
| **Zhang et al. (2022)** | TCM+Biomed | Biomed | LDL-C | 3 | -1 | 0 | -1 | 0 | -1 | Very low | -0.28 (-0.96, 0.39) |
| **Zhang et al. (2022)** | TCM+Biomed | Biomed | HDL-C | 2 | -1 | 0 | -1 | 0 | -1 | Very low | -0.07 (-0.25, 0.11) |
| **Zhang et al. (2022)** | TCM+Biomed | Biomed | FPG | 3 | -1 | 0 | -1 | -1 | -1 | Very low | -3.46 (-6.28, -0.63) |
| **Zhang et al. (2022)** | TCM+Biomed | Biomed | FINS | 2 | -1 | 0 | 0 | -1 | -1 | Very low | -2.52(-3.07, -1.96) |
| **Zhang et al. (2022)** | TCM+Biomed | Biomed | HOMA-IR | 2 | -1 | 0 | -1 | -1 | -1 | Very low | -1.41 (-2.67, -0.16) |
| **Liu et al. (2022)** | TCM+Biomed | Biomed | Overall efficacy | 5 | -1 | 0 | 0 | -1 | 0 | Low | 1.31 (1.18, 1.46) |
| **Liu et al. (2022)** | TCM | Biomed | Overall efficacy | 7 | -1 | 0 | 0 | -1 | 0 | Low | 1.16 (1.09, 1.24) |
| **Liu et al. (2022)** | TCM+Biomed | Biomed | TC | 11 | -1 | 0 | -1 | -1 | 0 | Very low | -0.94(-1.18, -0.71) |
| **Liu et al. (2022)** | TCM+Biomed | Biomed | TG | 11 | -1 | 0 | -1 | -1 | 0 | Very low | -0.63 (-0.84,-0.42) |
| **Liu et al. (2022)** | TCM+Biomed | Biomed | ALT | 11 | -1 | 0 | -1 | 0 | 0 | Low | -16.73(-21.32, -12.15) |
| **Liu et al. (2022)** | TCM+Biomed | Biomed | AST | 10 | -1 | 0 | -1 | 0 | 0 | Low | -14.36 (-19.18, -9.54) |
| **Liu et al. (2022)** | TCM+Biomed | Biomed | GGT | 2 | -1 | 0 | -1 | -1 | 0 | Low | -12.32 (-19.67, -4.98) |
| **Liu et al. (2022)** | TCM+Biomed | Biomed | Adpn | 5 | -1 | 0 | 0 | 0 | 0 | Medium | 2.04 (1.47, 2.60) |
| **Liu et al. (2022)** | TCM+Biomed | Biomed | HDL-C | 4 | -1 | 0 | -1 | -1 | 0 | Very low | 0.04I(-0.19, 0.28) |
| **Liu et al. (2022)** | TCM+Biomed | Biomed | LDL-C | 2 | -1 | 0 | -1 | -1 | 0 | Very low | -0.42 (-0.87, 0.04) |
| **Liu et al. (2022)** | TCM+Biomed | Biomed | BMI | 4 | -1 | 0 | -1 | -1 | 0 | Very low | -2.23 (-4.73, 0.26) |
| **Liu et al. (2022)** | TCM+Biomed | Biomed | Overall efficacy | 4 | -1 | 0 | 0 | -1 | 0 | Low | 1.17 (1.08, 1.27) |
| **Liu et al. (2022)** | TCM+Biomed | Biomed | Overall efficacy | 4 | -1 | 0 | 0 | -1 | 0 | Low | 1.28(1.08, 1.27) |
| **Liu et al. (2022)** | TCM+Biomed | Biomed | Overall efficacy | 6 | -1 | 0 | 0 | -1 | 0 | Low | 1.22(1.11, 1.35) |
| **Liu et al. (2022)** | TCM+Biomed | Biomed | Overall efficacy | 5 | -1 | 0 | 0 | -1 | 0 | Low | 1.20(1.14, 1.27) |
| **Shi et al. (2012)** | TCM | Biomed | ALT | 10 | -1 | 0 | -1 | -1 | -1 | Very low | 1.73 (1.34, 2.23) |
| **Shi et al. (2012)** | TCM | Biomed | ALT | 8 | -1 | 0 | -1 | -1 | -1 | Very low | 1.45(1.05, 1.98) |
| **Shi et al. (2012)** | TCM+Biomed | Biomed | ALT | 4 | -1 | 0 | -1 | -1 | -1 | Very low | 1.55 (1.08, 2.23) |
| **Shi et al. (2012)** | TCM+Biomed | Biomed | ALT | 1 | -1 | 0 | -1 | -1 | -1 | Very low | 1.67(0.53, 5.28) |
| **Shi et al. (2012)** | TCM+Biomed | Biomed | ALT | 1 | -1 | 0 | -1 | -1 | -1 | Very low | 3.31(0.82, 13.42) |
| **Shi et al. (2012)** | TCM | Biomed | ALT | 8 | -1 | 0 | -1 | -1 | -1 | Very low | 2.36(1.55, 3.6) |
| **Shi et al. (2012)** | TCM+Biomed | Biomed | ALT | 2 | -1 | 0 | -1 | -1 | -1 | Very low | 1.47(0.84, 2,56) |
| **Shi et al. (2012)** | TCM | Biomed | ALT | 8 | -1 | 0 | -1 | -1 | -1 | Very low | 1.43(1.03, 2) |
| **Shi et al. (2012)** | TCM+Biomed | Biomed | ALT | 7 | -1 | 0 | -1 | -1 | -1 | Very low | 1.51(1.11, 2.05) |
| **Shi et al. (2012)** | TCM | Biomed | ALT | 9 | -1 | 0 | -1 | -1 | -1 | Very low | 1.48(1.15, 1.92) |
| **Shi et al. (2012)** | TCM+Biomed | Biomed | ALT | 5 | -1 | 0 | -1 | -1 | -1 | Very low | 1.62(1.06, 2.46) |
| **Shi et al. (2012)** | TCM | Biomed | Normalization of blood lipid | 10 | -1 | 0 | -1 | -1 | -1 | Very low | 3.31(0.81, 13.42) |
| **Shi et al. (2012)** | TCM | Biomed | Normalization of blood lipid | 8 | -1 | 0 | -1 | -1 | -1 | Very low | 1.74(1.34, 2.26) |
| **Shi et al. (2012)** | TCM+Biomed | Biomed | Normalization of blood lipid | 4 | -1 | 0 | -1 | -1 | -1 | Very low | 1.57(0.88, 2.8) |
| **Shi et al. (2012)** | TCM+Biomed | Biomed | Normalization of blood lipid | 1 | -1 | 0 | -1 | -1 | -1 | Very low | 1.65(0.71, 3.87) |
| **Shi et al. (2012)** | TCM+Biomed | Biomed | Normalization of blood lipid | 1 | -1 | 0 | -1 | -1 | -1 | Very low | 1.67(0.53, 5.28) |
| **Shi et al. (2012)** | TCM | Biomed | Normalization of blood lipid | 8 | -1 | 0 | -1 | -1 | -1 | Very low | 2.13(1.34, 3.39) |
| **Shi et al. (2012)** | TCM+Biomed | Biomed | Normalization of blood lipid | 2 | -1 | 0 | -1 | -1 | -1 | Very low | 1.83(0.89, 3.75) |
| **Shi et al. (2012)** | TCM | Biomed | Normalization of blood lipid | 8 | -1 | 0 | -1 | -1 | -1 | Very low | 1.26(0.85, 1.87) |
| **Shi et al. (2012)** | TCM+Biomed | Biomed | Normalization of blood lipid | 7 | -1 | 0 | -1 | -1 | -1 | Very low | 1.32(0.91, 1.93) |
| **Shi et al. (2012)** | TCM | Biomed | Normalization of blood lipid | 9 | -1 | 0 | -1 | -1 | -1 | Very low | 1.53(0.94, 2.51) |
| **Shi et al. (2012)** | TCM+Biomed | Biomed | Normalization of blood lipid | 5 | -1 | 0 | -1 | -1 | -1 | Very low | 1.67(0.95, 2.94) |
| **Shi et al. (2012)** | TCM | Biomed | Disappearance of radiological steatosis | 10 | -1 | 0 | -1 | -1 | -1 | Very low | 5.51(0.25, 119.50) |
| **Shi et al. (2012)** | TCM | Biomed | Disappearance of radiological steatosis | 8 | -1 | 0 | -1 | -1 | -1 | Very low | 2.43(1.48, 3.97) |
| **Shi et al. (2012)** | TCM+Biomed | Biomed | Disappearance of radiological steatosis | 4 | -1 | 0 | -1 | -1 | -1 | Very low | 1.92(1.2, 3.07) |
| **Shi et al. (2012)** | TCM+Biomed | Biomed | Disappearance of radiological steatosis | 1 | -1 | 0 | -1 | -1 | -1 | Very low | 1.94(1.28, 2.96) |
| **Shi et al. (2012)** | TCM+Biomed | Biomed | Disappearance of radiological steatosis | 1 | -1 | 0 | -1 | -1 | -1 | Very low | 1.67(0.53, 5.28) |
| **Shi et al. (2012)** | TCM | Biomed | Disappearance of radiological steatosis | 8 | -1 | 0 | -1 | -1 | -1 | Very low | 2.35(1.61, 3.41) |
| **Shi et al. (2012)** | TCM+Biomed | Biomed | Disappearance of radiological steatosis | 2 | -1 | 0 | -1 | -1 | -1 | Very low | 1.8(0.76, 4.29) |
| **Shi et al. (2012)** | TCM | Biomed | Disappearance of radiological steatosis | 8 | -1 | 0 | -1 | -1 | -1 | Very low | 1.76(1.3, 2.37) |
| **Shi et al. (2012)** | TCM+Biomed | Biomed | Disappearance of radiological steatosis | 7 | -1 | 0 | -1 | -1 | -1 | Very low | 2.13(1.42, 3.19) |
| **Shi et al. (2012)** | TCM | Biomed | Disappearance of radiological steatosis | 9 | -1 | 0 | -1 | -1 | -1 | Very low | 1.81(1.27, 2.58) |
| **Shi et al. (2012)** | TCM+Biomed | Biomed | Disappearance of radiological steatosis | 5 | -1 | 0 | -1 | -1 | -1 | Very low | 1.77(1.1, 2.84) |
| **Ding et al. (2021)** | TCM+Biomed | Biomed | Overall efficacy | 9 | -1 | 0 | 0 | -1 | 0 | Low | 6.07 (3.63, 10.15) |
| **Ding et al. (2021)** | TCM+Biomed | Biomed | ALT | 9 | -1 | 0 | 0 | -1 | 0 | Low | -8.38(-9.56, -7.21) |
| **Ding et al. (2021)** | TCM+Biomed | Biomed | AST | 9 | -1 | 0 | 0 | -1 | 0 | Low | -9.77(-10.93, -8.61) |
| **Ding et al. (2021)** | TCM+Biomed | Biomed | TG | 9 | -1 | 0 | 0 | -1 | 0 | Low | -0.48(-0.53, -0.43) |
| **Ding et al. (2021)** | TCM+Biomed | Biomed | TC | 9 | -1 | 0 | 0 | -1 | 0 | Low | -0.73(-0.81, -0.65) |
| **Mou et al. (2017)** | TCM | Biomed/placebo | Overall efficacy | 6 | -1 | 0 | -1 | -1 | 0 | Very low | 0.37(0.29, 0.49) |
| **Mou et al. (2017)** | TCM | Biomed/placebo | AST | 8 | -1 | 0 | -1 | -1 | 0 | Very low | -8.96(-20.62, 2.69) |
| **Mou et al. (2017)** | TCM | Biomed/placebo | ALT | 8 | -1 | 0 | -1 | -1 | 0 | Very low | -6.87(-15.36, 1.63) |
| **Mou et al. (2017)** | TCM | Biomed/placebo | TG | 7 | -1 | 0 | -1 | -1 | 0 | Very low | -0.33(-0.66, 0.01) |
| **Mou et al. (2017)** | TCM | Biomed/placebo | TC | 8 | -1 | 0 | -1 | -1 | 0 | Very low | -0.76(-1.21, -0.31) |
| **Mou et al. (2017)** | TCM | Biomed/placebo | Liver/spleen computed tomography ratio | 3 | -1 | 0 | 0 | -1 | 0 | Low | 0.12(0.02,0.23) |
| **Zhao et al. (2022)** | TCM | Biomed | Overall efficacy | 10 | -1 | 0 | 0 | -1 | 0 | Low | 3.58(2.38, 5.39) |
| **Zhao et al. (2022)** | TCM | Biomed | ALT | 11 | -1 | 0 | -1 | -1 | 0 | Very low | 3.27(-5.95, -0.59) |
| **Zhao et al. (2022)** | TCM | Biomed | AST | 10 | -1 | 0 | -1 | -1 | 0 | Very low | -3.23(-6.11, -0.35) |
| **Zhao et al. (2022)** | TCM | Biomed | TC | 9 | -1 | 0 | -1 | -1 | 0 | Very low | -0.61(-0.94, -0.28) |
| **Zhao et al. (2022)** | TCM | Biomed | TG | 10 | -1 | 0 | -1 | -1 | 0 | Very low | -0.37(-0.59, -0.16) |
| **Zhao et al. (2022)** | TCM | Biomed | Effective improvement rate of B-ultrasound | 2 | -1 | 0 | 0 | -1 | 0 | Low | 3.06(1.35, 6.91) |
| **Yang et al. (2019)** | TCM | Biomed | Overall efficacy | 17 | -1 | 0 | -1 | -1 | -1 | Very low | 1.30(1.16, 1.46) |
| **Yang et al. (2019)** | TCM+Biomed | Biomed | Overall efficacy | 2 | -1 | 0 | 0 | 0 | 0 | Medium | 1.33(1.14, 1.55) |
| **Yang et al. (2019)** | TCM | Biomed | ALT | 15 | -1 | 0 | -1 | -1 | -1 | Very low | -8.55(-12.76, -4.34) |
| **Yang et al. (2019)** | TCM | Biomed | AST | 13 | -1 | 0 | -1 | 0 | 0 | Low | -3.60(-5.83,-1.37) |
| **Yang et al. (2019)** | TCM | Biomed | TC | 13 | -1 | 0 | -1 | 0 | -1 | Very low | -0.88(-1.15, -0.61) |
| **Yang et al. (2019)** | TCM | Biomed | TG | 14 | -1 | 0 | -1 | 0 | -1 | Very low | -0.4 7(-0.65, -0.30) |
| **Wu et al. (2017)** | TCM | Biomed | Overall efficacy | 14 | -1 | 0 | 0 | -1 | 0 | Low | 1.20(1.11, 1.31) |
| **Wu et al. (2017)** | TCM | Biomed | ALT | 14 | -1 | 0 | -1 | -1 | 0 | Very low | -4.51(-5.79, -2.23) |
| **Wu et al. (2017)** | TCM | Biomed | AST | 13 | -1 | 0 | -1 | -1 | 0 | Very low | -2.57(-4.07, -1.06) |
| **Wu et al. (2017)** | TCM | Biomed | GGT | 14 | -1 | 0 | -1 | -1 | 0 | Very low | -10.52(-19.92, -1.73) |
| **Wu et al. (2017)** | TCM | Biomed | TC | 12 | -1 | 0 | -1 | -1 | 0 | Very low | -0.59(-0.84, -0.34) |
| **Wu et al. (2017)** | TCM | Biomed | TG | 13 | -1 | 0 | -1 | -1 | 0 | Very low | -0.35(-0.52, -0.17) |
| **Wu et al. (2017)** | TCM | Biomed | HDL-C | 5 | -1 | 0 | -1 | -1 | 0 | Very low | 0.2(0.06, 0.33) |
| **Wu et al. (2017)** | TCM | Biomed | LDL-C | 4 | -1 | 0 | -1 | -1 | 0 | Very low | -0.51(-0.9, -0.13) |
| **Wu et al. (2017)** | TCM | Biomed | Effective improvement rate of B-ultrasound | 4 | -1 | 0 | 0 | -1 | 0 | Low | 2.74(1.36, 5.55) |
| **Qin et al. (2022)** | TCM | Biomed | ALT | 6 | -1 | 0 | -1 | -1 | 0 | Very low | -11.69(15.31, -8.06) |
| **Qin et al. (2022)** | TCM | Biomed | AST | 5 | -1 | 0 | -1 | -1 | 0 | Very low | -8.48(-13.62, -3.35) |
| **Qin et al. (2022)** | TCM | Biomed | TC | 4 | -1 | 0 | -1 | -1 | 0 | Very low | -0.88(-1.19, -0.56) |
| **Qin et al. (2022)** | TCM | Biomed | TG | 5 | -1 | 0 | -1 | -1 | 0 | Very low | -0.98(-1.60, -0.36) |
| **Qin et al. (2022)** | TCM | Biomed | HOMA-IR | 2 | -1 | 0 | 0 | -1 | 0 | Low | -1.39(-1.62, -1.15) |
| **Gao et al. (2020)** | TCM | Biomed | Overall efficacy | 12 | -1 | 0 | 0 | 0 | 0 | Medium | 4.19(2.92, 6.01) |
| **Gao et al. (2020)** | TCM | Biomed | ALT | 12 | -1 | 0 | -1 | 0 | 0 | Low | -15.36(-21.51,-9.20) |
| **Gao et al. (2020)** | TCM | Biomed | AST | 12 | -1 | 0 | -1 | 0 | 0 | Low | -11.70(-15.96, -7.43) |
| **Gao et al. (2020)** | TCM | Biomed | GGT | 9 | -1 | 0 | -1 | 0 | 0 | Low | -24.74(-32.47, -17.02) |
| **Gao et al. (2020)** | TCM | Biomed | TC | 12 | -1 | 0 | -1 | 0 | 0 | Low | -1.03(-1.46, -0.60) |
| **Gao et al. (2020)** | TCM | Biomed | TG | 12 | -1 | 0 | -1 | 0 | 0 | Low | -0.70(-0.94, -0.45) |
| **Gao et al. (2020)** | TCM | Biomed | HDL-C | 7 | -1 | 0 | 0 | 0 | 0 | Medium | 0.27(0.24, 0.30) |
| **Gao et al. (2020)** | TCM | Biomed | LDL-C | 7 | -1 | 0 | -1 | 0 | 0 | Low | -0.46(-0.67, -0.25) |
| **Gao et al. (2020)** | TCM | Biomed | Effective improvement rate of B-ultrasound | 2 | -1 | 0 | 0 | 0 | 0 | Medium | 3.38(1.99, 5.76) |
| **Gao et al. (2020)** | TCM | Biomed | BMI | 4 | -1 | 0 | -1 | 0 | 0 | Low | -1.57(-2.64, 0.50) |
| **Gong et al. (2014)** | TCM | Biomed | Overall efficacy | 17 | -1 | 0 | 0 | 0 | 0 | Medium | 4.33(3.26, 5.74) |
| **Gong et al. (2014)** | TCM | Biomed | ALT | 13 | -1 | 0 | -1 | 0 | 0 | Low | -14.80(-21.63, 7.96) |
| **Gong et al. (2014)** | TCM | Biomed | AST | 12 | -1 | 0 | -1 | 0 | 0 | Low | -9.48(-13.95, -5.01) |
| **Gong et al. (2014)** | TCM | Biomed | TC | 12 | -1 | 0 | -1 | 0 | 0 | Low | -0.70(-1.01, 0.39) |
| **Gong et al. (2014)** | TCM | Biomed | TG | 12 | -1 | 0 | -1 | 0 | 0 | Low | -0.72(-0.958, 0.149) |
| **Gong et al. (2014)** | TCM | Biomed | Effective improvement rate of B-ultrasound | 5 | -1 | 0 | 0 | 0 | 0 | Medium | 2.33(1.41, 3.82) |
| **Zhang et al. (2014)** | TCM | Biomed | ALT | 13 | -1 | 0 | -1 | 0 | -1 | Very low | -13.61(-20.11, -7.10) |
| **Zhang et al. (2014)** | TCM | Biomed | AST | 12 | -1 | 0 | -1 | 0 | -1 | Very low | -8.88(-14.78, -2.97) |
| **Zhang et al. (2014)** | TCM | Biomed | GGT | 8 | -1 | 0 | -1 | 0 | -1 | Very low | -9.78(15.43, -4.13) |
| **Zhang et al. (2014)** | TCM | Biomed | TG | 12 | -1 | 0 | -1 | 0 | -1 | Very low | -0.85(-1.48, -0.22) |
| **Zhang et al. (2014)** | TCM | Biomed | TC | 12 | -1 | 0 | -1 | 0 | -1 | Very low | -0.43(-0.11, -0.14) |
| **Zhang et al. (2014)** | TCM | Biomed | HDL-C | 5 | -1 | 0 | -1 | 0 | -1 | Very low | 0.01(-0.11, 0.14) |
| **Zhang et al. (2014)** | TCM | Biomed | LDL-C | 5 | -1 | 0 | -1 | 0 | -1 | Very low | -0.50(-0.77, -0.23) |
| **Zhang et al. (2014)** | TCM | Biomed | BMI | 2 | -1 | 0 | -1 | 0 | -1 | Very low | 0..38(-3.94, 4.69) |
| **Zhang et al. (2014)** | TCM | Biomed | Liver/spleen computed tomography ratio | 3 | -1 | 0 | -1 | 0 | -1 | Very low | -3.63(-6.60, -0.65) |
| **Zhang et al. (2014)** | TCM | Biomed | Overall efficacy | 12 | -1 | 0 | 0 | 0 | -1 | low | 0.07(-0.01, 0.16) |
| **Zhang et al. (2014)** | TCM | Biomed | Effective improvement rate of B-ultrasound | 5 | -1 | 0 | 0 | 0 | -1 | low | 0.31(0.19, 0.50) |
| **Dong et al. (2023)** | TCM | Biomed | Overall efficacy | 7 | -1 | 0 | -1 | 0 | -1 | Very low | 1.28(1.18, 1.39) |
| **Dong et al. (2023)** | TCM | Biomed | ALT | 7 | -1 | 0 | -1 | 0 | -1 | Very low | -12.31(-18.54, -6.07) |
| **Dong et al. (2023)** | TCM | Biomed | AST | 7 | -1 | 0 | -1 | 0 | -1 | Very low | -13.02(-20.14, -5.91) |
| **Dong et al. (2023)** | TCM | Biomed | GGT | 6 | -1 | 0 | -1 | 0 | -1 | Very low | -21.75(-23.17, -20.34) |
| **Dong et al. (2023)** | TCM | Biomed | TG | 5 | -1 | 0 | -1 | 0 | -1 | Very low | -21.96(-22.77, -21.16) |
| **Dong et al. (2023)** | TCM | Biomed | TC | 5 | -1 | 0 | -1 | 0 | -1 | Very low | -0.63(-0.74, -0.53) |
| **Zhang et al. (2024)** | TCM+Biomed | Biomed | ALT | 31 | -1 | 0 | -1 | 0 | 0 | Low | -6.03(-7.51, -5.09 |
| **Zhang et al. (2024)** | TCM+Biomed | Biomed | AST | 31 | -1 | 0 | -1 | 0 | 0 | Low | -5.26(-6.21, -4.32) |
| **Zhang et al. (2024)** | TCM+Biomed | Biomed | FPG | 34 | -1 | 0 | -1 | 0 | 0 | Low | -0.68(-0.83, -0.53) |
| **Zhang et al. (2024)** | TCM+Biomed | Biomed | 2hpg | 29 | -1 | 0 | -1 | 0 | 0 | Low | -1.16(-1.44, -0.88) |
| **Zhang et al. (2024)** | TCM+Biomed | Biomed | HOMA-IR | 22 | -1 | 0 | -1 | 0 | 0 | Low | -0.99(-1.25, -0.74) |
| **Zhang et al. (2024)** | TCM+Biomed | Biomed | TG | 33 | -1 | 0 | -1 | 0 | 0 | Low | -0.56(-0.69 -0.43) |
| **Zhang et al. (2024)** | TCM+Biomed | Biomed | TC | 31 | -1 | 0 | -1 | 0 | 0 | Low | -0.65(-0.84, -0.46) |
| **Zhang et al. (2024)** | TCM+Biomed | Biomed | LDL-C | 30 | -1 | 0 | -1 | 0 | 0 | Low | -0.44(-0.56, -0.32) |
| **Zhang et al. (2024)** | TCM+Biomed | Biomed | HDL-C | 25 | -1 | 0 | -1 | 0 | 0 | Low | 0.19(0.09, 0.29) |
| **Han et al. (2024)** | TCM+Biomed | Biomed | ALT | 21 | -1 | 0 | -1 | 0 | 0 | Low | -15.99(-19.47, -12.52) |
| **Han et al. (2024)** | TCM+Biomed | Biomed | AST | 21 | -1 | 0 | -1 | 0 | 0 | Low | -13.39(-16.79, -9.99) |
| **Han et al. (2024)** | TCM+Biomed | Biomed | GGT | 21 | -1 | 0 | -1 | 0 | 0 | Low | -14.37(-19.59, -9.16) |
| **Han et al. (2024)** | TCM+Biomed | Biomed | TC | 21 | -1 | 0 | -1 | 0 | 0 | Low | -0.80(-1.04, -0.57) |
| **Han et al. (2024)** | TCM+Biomed | Biomed | TG | 21 | -1 | 0 | -1 | 0 | 0 | Low | -0.47(-0.55, -0.38) |
| **Zhang et al. (2014)** | TCM | Biomed | Overall efficacy | 10 | -1 | 0 | 0 | 0 | 0 | Medium | 1.51(1.41, 1.62) |
| **Liu et al. (2023)** | TCM | Biomed | ALT | 18 | -1 | 0 | -1 | 0 | 0 | low | -10.20 (-11.27, -9.13) |
| **Liu et al. (2023)** | TCM+Biomed | Biomed | ALT | 7 | -1 | 0 | -1 | 0 | 0 | low | -26.65 (-29.85, -23.45) |
| **Liu et al. (2023)** | TCM | Biomed | AST | 18 | -1 | 0 | -1 | 0 | 0 | low | -10.03 (-11.00, -9.06) |
| **Liu et al. (2023)** | TCM+Biomed | Biomed | AST | 7 | -1 | 0 | -1 | 0 | 0 | low | -17.65 (-19.67, -15.62) |
| **Liu et al. (2023)** | TCM | Biomed | Effective improvement rate of B-ultrasound | 12 | -1 | 0 | 0 | 0 | 0 | low | 2.63 (1.91, 3.61) |
| **Li et al. (2011)** | TCM | Biomed | Overall efficacy | 5 | -1 | 0 | -1 | -1 | -1 | Very low | 1.43(0.97, 2.10) |
| **Li et al. (2011)** | TCM | Biomed | Overall efficacy | 11 | -1 | 0 | -1 | -1 | -1 | Very low | 2.05(1.43, 2.93) |
| **Li et al. (2011)** | TCM | Biomed | Overall efficacy | 2 | -1 | 0 | 0 | 0 | -1 | low、 | 1.29(1.08, 1.53) |
| **Li et al. (2011)** | TCM | Biomed | Overall efficacy | 2 | -1 | 0 | -1 | -1 | -1 | Very low | 1.48(1.15, 1.91) |
| **Li et al. (2011)** | TCM | Biomed | ALT | 6 | -1 | 0 | -1 | -1 | -1 | Very low | 0.99(0.92, 1.07) |
| **Li et al. (2011)** | TCM | Biomed | ALT | 8 | -1 | 0 | -1 | -1 | -1 | Very low | 1.39(1.30, 1.50) |
| **Li et al. (2011)** | TCM | Biomed | ALT | 3 | -1 | 0 | -1 | -1 | -1 | Very low | 1.24(1.10, 1.39) |
| **Li et al. (2011)** | TCM | Biomed | ALT | 1 | -1 | 0 | -1 | -1 | -1 | Very low | 1.35(1.14, 1.60) |
| **Li et al. (2011)** | TCM | Biomed | AST | 3 | -1 | 0 | -1 | -1 | -1 | Very low | -9.94(-24.52, 4.65) |
| **Li et al. (2011)** | TCM | Biomed | AST | 7 | -1 | 0 | -1 | -1 | -1 | Very low | -25.08(-44.81, -5.36) |
| **Li et al. (2011)** | TCM | Biomed | AST | 3 | -1 | 0 | -1 | -1 | -1 | Very low | -20.73(-34.27, -7.19) |
| **Li et al. (2011)** | TCM | Biomed | AST | 1 | -1 | 0 | -1 | -1 | -1 | Very low | -17.10(-21.03, -13.17) |
| **Li et al. (2011)** | TCM | Biomed | GGT | 4 | -1 | 0 | -1 | -1 | -1 | Very low | -0.88(-7.78, 6.02) |
| **Li et al. (2011)** | TCM | Biomed | GGT | 4 | -1 | 0 | -1 | -1 | -1 | Very low | -8.07(-14.47, -1.67) |
| **Li et al. (2011)** | TCM | Biomed | GGT | 3 | -1 | 0 | -1 | 0 | -1 | Very low | -23.59(-30.01, -17.16) |
| **Li et al. (2011)** | TCM | Biomed | GGT | 1 | -1 | 0 | -1 | -1 | -1 | Very low | -16.20(-20.96,-11.44) |
| **Li et al. (2011)** | TCM | Biomed | TG | 6 | -1 | 0 | -1 | -1 | -1 | Very low | 0.04(-0.51, 0.60) |
| **Li et al. (2011)** | TCM | Biomed | TG | 6 | -1 | 0 | -1 | -1 | -1 | Very low | -0.88(-1.80, 0.05) |
| **Li et al. (2011)** | TCM | Biomed | TG | 3 | -1 | 0 | -1 | 0 | -1 | Very low | -0.89(-1.14, -0.65) |
| **Li et al. (2011)** | TCM | Biomed | TG | 2 | -1 | 0 | -1 | -1 | -1 | Very low | -0.11(-0.36, 0.13) |
| **Li et al. (2011)** | TCM | Biomed | LDL-C | 1 | -1 | 0 | -1 | -1 | -1 | Very low | 0.04(-0.51, 0.60) |
| **Li et al. (2011)** | TCM | Biomed | LDL-C | 2 | -1 | 0 | 0 | 0 | -1 | low | -0.88(-1.80, 0.05) |
| **Li et al. (2011)** | TCM | Biomed | LDL-C | 3 | -1 | 0 | -1 | 0 | -1 | Very low | -0.89(-1.14, -0.65) |
| **Li et al. (2011)** | TCM | Biomed | LDL-C | 1 | -1 | 0 | -1 | 0 | -1 | Very low | -0.11(-0.36, 0.13) |
| **Li et al. (2011)** | TCM | Biomed | HDL-C | 2 | -1 | 0 | -1 | -1 | -1 | Very low | -0.30(-0.65, 0.05) |
| **Li et al. (2011)** | TCM | Biomed | HDL-C | 3 | -1 | 0 | 0 | 0 | -1 | low | -0.36(-0.61, -0.10) |
| **Li et al. (2011)** | TCM | Biomed | HDL-C | 2 | -1 | 0 | -1 | -1 | -1 | Very low | -0.63(-0.64, -0.62) |
| **Li et al. (2011)** | TCM | Biomed | HDL-C | 1 | -1 | 0 | 0 | 0 | -1 | low | -0.39(-0.65, -0.13) |
| **Li et al. (2011)** | TCM | Biomed | TC | 6 | -1 | 0 | -1 | -1 | -1 | Very low | 0.24(-0.19, 0.67) |
| **Li et al. (2011)** | TCM | Biomed | TC | 6 | -1 | 0 | -1 | -1 | -1 | Very low | 0.02(-0.07, 0.11) |
| **Li et al. (2011)** | TCM | Biomed | TC | 3 | -1 | 0 | -1 | -1 | -1 | Very low | -0.02(-0.73, 0.68) |
| **Li et al. (2011)** | TCM | Biomed | TC | 2 | -1 | 0 | -1 | -1 | -1 | Very low | 0.12(0.06, 0.18) |
| **Cai et al. (2019)** | TCM | Biomed | Effective improvement rate of B-ultrasound | 7 | -1 | 0 | 0 | -1 | 0 | LOW | 2.33(1.60, 3.40) |
| **Cai et al. (2019)** | TCM | Biomed | TC | 5 | -1 | 0 | -1 | -1 | 0 | Very low | 0.38(-0.48, -0.291 |
| **Cai et al. (2019)** | TCM | Biomed | TG | 6 | -1 | 0 | -1 | -1 | 0 | Very low | -0.31(-0.37, -0.24) |
| **Cai et al. (2019)** | TCM | Biomed | ALT | 6 | -1 | 0 | -1 | -1 | 0 | Very low | -1.69(-2.24, -1.14) |
| **Cai et al. (2019)** | TCM | Biomed | AST | 5 | -1 | 0 | -1 | -1 | 0 | Very low | -22.53(-33.16, -11.90) |
| **Cai et al. (2019)** | TCM | Biomed | Overall efficacy | 12 | -1 | 0 | 0 | -1 | 0 | LOW | 3.55(2.65, 4.76) |
| **D.W. Rathnayake et al. (2024)** | TCM+Biomed | Biomed+placebo | ALT | 39 | 0 | 0 | -1 | 0 | 0 | Medium | -7.23 (-4.82, -9.64) |
| **D.W. Rathnayake et al. (2024)** | TCM+Biomed | Biomed+placebo | AST | 29 | 0 | 0 | 0 | 0 | 0 | High | -3.08 (-5.23, -0.92) |
| **D.W. Rathnayake et al. (2024)** | TCM+Biomed | Biomed+placebo | BMI | 27 | 0 | 0 | 0 | 0 | 0 | High | -0.38 (-0.71, -0.05) |
| **D.W. Rathnayake et al. (2024)** | TCM+Biomed | Biomed+placebo | TG | 18 | 0 | 0 | 0 | 0 | 0 | High | -20.05 (-29.67, -10.42) |
| **D.W. Rathnayake et al. (2024)** | TCM+Biomed | Biomed+placebo | HDL-C | 19 | 0 | 0 | 0 | 0 | 0 | High | 0.53 (0.04, 1.02) |
| **D.W. Rathnayake et al. (2024)** | TCM+Biomed | Biomed+placebo | FPG | 18 | 0 | 0 | 0 | 0 | 0 | High | -2.03(-4.10, -0.01) |
| **D.W. Rathnayake et al. (2024)** | TCM+Biomed | Biomed+placebo | HbA1c | 4 | 0 | 0 | 0 | 0 | 0 | High | -0.01 (-0.12, -0.11) |

**TCM:** Traditional Chinese Medicine; **Biomed:** botanical drug; **ALT:** Alanine aminotransferase; **AST:** Aspartate aminotransferase; **GGT:** Gamma-glutamyl transpeptidase; **TBiL:**Total Bilirubin; **TC:** Total cholesterol; **TG:**Triglyceride; **HDL-C:** High-density lipoprotein cholesterol; **LDL-C:** Low-density lipoprotein cholesterol; **FBG:** fasting blood glucose; **FINS:**fasting serum insulin; **2hPG:**2-h postprandial blood glucose; **HOMA-IR:** homeostatic model assessment for insulin resistance; **HbA1c:** glycosylated hemoglobin type A1c; **Adpn:**adiponectin ; **NAFLD:**non-alcoholic fatty liver disease; **CT：**computerized tomography;

**Supplementary Material 3. The 16 items of AMSTAR-2.**

| **Item #** | **Checklist item** |
| --- | --- |
| 1 | Did the research questions and inclusion criteria for the review include the components of PICO (Population, Intervention, Comparison, Outcome)? |
| 2 | Did the report of the review contain an explicit statement that review methods were established before conduct of the review? . Did the report justify any significant deviations from the protocol?* |
| 3 | Did review authors explain their selection of study designs for inclusion in the review? |
| 4 | Did review authors use a comprehensive literature search strategy?* |
| 5 | Did review authors perform study selection in duplicate? |
| 6 | Did review authors perform data extraction in duplicate? |
| 7 | Did review authors provide a list of excluded studies and justify exclusions?* |
| 8 | Did review authors describe the included studies in adequate detail? |
| 9 | Did review authors use a satisfactory technique for assessing risk of bias (RoB) in individual studies that were included in the review?* |
| 10 | Did review authors report on sources of funding for studies included in the review? |
| 11 | If meta-analysis was performed, did review authors use appropriate methods for statistical combination of results?* |
| 12 | If meta-analysis was performed, did review authors assess the potential impact of RoB of individual studies on results of the meta-analysis or other evidence synthesis? |
| 13 | Did review authors account for RoB in individual studies when interpreting/ discussing results of the review?* |
| 14 | Did review authors provide a satisfactory explanation for, and discussion of, any heterogeneity observed in results of the review? |
| 15 | If they performed quantitative synthesis, did review authors carry out an adequate investigation of publication bias (smallstudy bias) and discuss its likely impact on the results of the review?* |
| 16 | Did review authors report any potential conflicts of interest,including any funding received for conducting the review? |

**Supplementary Material 4. The checklists of PRISMA2020.**

| **Section and Topic** | **Item #** | **Checklist item** |
| --- | --- | --- |
| **TITLE** | | |
| Title | 1 | Identify the report as a systematic review. |
| **ABSTRACT** | | |
| Abstract | 2 | See the PRISMA 2020 for Abstracts checklist. |
| **INTRODUCTION** | | |
| Rationale | 3 | Describe the rationale for the review in the context of existing knowledge. |
| Objectives | 4 | Provide an explicit statement of the objective(s) or question(s) the review addresses. |
| **METHODS** | | |
| Eligibility criteria | 5 | Specify the inclusion and exclusion criteria for the review and how studies were grouped for the syntheses. |
| Information sources | 6 | Specify all databases, registers, websites, organisations, reference lists and other sources searched or consulted to identify studies. Specify the date when each source was last searched or consulted. |
| Search strategy | 7 | Present the full search strategies for all databases, registers and websites, including any filters and limits used. |
| Selection process | 8 | Specify the methods used to decide whether a study met the inclusion criteria of the review, including how many reviewers screened each record and each report retrieved, whether they worked independently, and if applicable, details of automation tools used in the process. |
| Data collection process | 9 | Specify the methods used to collect data from reports, including how many reviewers collected data from each report, whether they worked independently, any processes for obtaining or confirming data from study investigators, and if applicable, details of automation tools used in the process. |
| Data items | 10a | List and define all outcomes for which data were sought. Specify whether all results that were compatible with each outcome domain in each study were sought (e.g. for all measures, time points, analyses), and if not, the methods used to decide which results to collect. |
|  | 10b | List and define all other variables for which data were sought (e.g. participant and intervention characteristics, funding sources). Describe any assumptions made about any missing or unclear information. |
| Study risk of bias assessment | 11 | Specify the methods used to assess risk of bias in the included studies, including details of the tool(s) used, how many reviewers assessed each study and whether they worked independently, and if applicable, details of automation tools used in the process. |
| Effect measures | 12 | Specify for each outcome the effect measure(s) (e.g. risk ratio, mean difference) used in the synthesis or presentation of results. |
| Synthesis methods | 13a | Describe the processes used to decide which studies were eligible for each synthesis (e.g. tabulating the study intervention characteristics and comparing against the planned groups for each synthesis (item #5)). |
|  | 13b | Describe any methods required to prepare the data for presentation or synthesis, such as handling of missing summary statistics, or data conversions. |
|  | 13c | Describe any methods used to tabulate or visually display results of individual studies and syntheses. |
|  | 13d | Describe any methods used to synthesize results and provide a rationale for the choice(s). If meta-analysis was performed, describe the model(s), method(s) to identify the presence and extent of statistical heterogeneity, and software package(s) used. |
|  | 13e | Describe any methods used to explore possible causes of heterogeneity among study results (e.g. subgroup analysis, meta-regression). |
|  | 13f | Describe any sensitivity analyses conducted to assess robustness of the synthesized results. |
| Reporting bias assessment | 14 | Describe any methods used to assess risk of bias due to missing results in a synthesis (arising from reporting biases). |
| Certainty assessment | 15 | Describe any methods used to assess certainty (or confidence) in the body of evidence for an outcome. |
| **RESULTS** | | |
| Study selection | 16a | Describe the results of the search and selection process, from the number of records identified in the search to the number of studies included in the review, ideally using a flow diagram. |
|  | 16b | Cite studies that might appear to meet the inclusion criteria, but which were excluded, and explain why they were excluded. |
| Study characteristics | 17 | Cite each included study and present its characteristics. |
| Risk of bias in studies | 18 | Present assessments of risk of bias for each included study. |
| Results of individual studies | 19 | For all outcomes, present, for each study: (a) summary statistics for each group (where appropriate) and (b) an effect estimate and its precision (e.g. confidence/credible interval), ideally using structured tables or plots. |
| Results of syntheses | 20a | For each synthesis, briefly summarise the characteristics and risk of bias among contributing studies. |
|  | 20b | Present results of all statistical syntheses conducted. If meta-analysis was done, present for each the summary estimate and its precision (e.g. confidence/credible interval) and measures of statistical heterogeneity. If comparing groups, describe the direction of the effect. |
|  | 20c | Present results of all investigations of possible causes of heterogeneity among study results. |
|  | 20d | Present results of all sensitivity analyses conducted to assess the robustness of the synthesized results. |
| Reporting biases | 21 | Present assessments of risk of bias due to missing results (arising from reporting biases) for each synthesis assessed. |
| Certainty of evidence | 22 | Present assessments of certainty (or confidence) in the body of evidence for each outcome assessed. |
| **DISCUSSION** | | |
| Discussion | 23a | Provide a general interpretation of the results in the context of other evidence. |
|  | 23b | Discuss any limitations of the evidence included in the review. |
|  | 23c | Discuss any limitations of the review processes used. |
|  | 23d | Discuss implications of the results for practice, policy, and future research. |
| **OTHER INFORMATION** | | |
| Registration and protocol | 24a | Provide registration information for the review, including register name and registration number, or state that the review was not registered. |
|  | 24b | Indicate where the review protocol can be accessed, or state that a protocol was not prepared. |
|  | 24c | Describe and explain any amendments to information provided at registration or in the protocol. |
| Support | 25 | Describe sources of financial or non-financial support for the review, and the role of the funders or sponsors in the review. |
| Competing interests | 26 | Declare any competing interests of review authors. |
| Availability of data, code and other materials | 27 | Report which of the following are publicly available and where they can be found: template data collection forms; data extracted from included studies; data used for all analyses; analytic code; any other materials used in the review. |

**Supplementary Material 5. The checklists of PRISMA2020 for Abstracts.**

| **Section and topic** | **Item #** | **Checklist item** |
| --- | --- | --- |
| **Title** | | |
| Title | 1 | Identify the report as a systematic review. |
| **Background** | | |
| bjectives | 2 | Provide an explicit statement of the main objective(s) or question(s) the review addresses. |
| **Methods** | | |
| Eligibility criteria | 3 | Specify the inclusion and exclusion criteria for the review. |
| Information sources | 4 | Specify the information sources (e.g. databases, registers) used to identify studies and the date when each was last searched. |
| Risk of bias | 5 | Specify the methods used to assess risk of bias in the included studies. |
| Synthesis of results | 6 | Specify the methods used to present and synthesise results. |
| **Results** | | |
| Included studies | 7 | Give the total number of included studies and participants and summarise relevant characteristics of studies. |
| Synthesis of results | 8 | Present results for main outcomes, preferably indicating the number of included studies and participants for each. If meta-analysis was done, report the summary estimate and confidence/credible interval. If comparing groups, indicate the direction of the effect (i.e. which group is favoured). |
| **Discussion** | | |
| Limitations of evidence | 9 | Provide a brief summary of the limitations of the evidence included in the review (e.g. study risk of bias, inconsistency and imprecision). |
| Interpretation | 10 | Provide a general interpretation of the results and important implications. |
| **Other** | | |
| Funding | 11 | Specify the primary source of funding for the review. |
| Registration | 12 | Provide the register name and registration number. |

**Supplementary Material 6. The checklists of PRISMA-CHM.**

| **Section/Topic** | **Item Number** | **PRISMA Checklist Item** | **Extension for CHM** |
| --- | --- | --- | --- |
| **TITLE** | | | |
| Title | 1 | Identify the report as a systematic review, metaanalysis, or both. | 1a. Specify the name of the CHM intervention, in terms of (1) Chinese medicinal substance(s), and/or (2) CHM formula(s). 1b. State whether the review targets (1) Western medicine-deﬁned disease(s), or (2) Western medicine-deﬁned disease(s) with speciﬁc TCM Pattern(s), or (3) TCM Pattern(s). |
| **ABSTRACT** | | | |
| Structured summary | 2 | Provide a structured summary including, as applicable: background; objectives; data sources; study eligibility criteria, participants, and interventions; study appraisal and synthesis methods; results; limitations; conclusions and implications of key ﬁndings; systematic review registration number. | Provide the name and form of the CHM inter-vention(s) used, and the TCM Pattern applied (if any). |
| **INTRODUCTION** | | | |
| Rationale | 3 | Describe the rationale for the review in the context of what is already known. | 3a. State the rationale of using particular CHM intervention(s) to target the speciﬁc disease(s) and/or TCM Pattern (if any),ideally in terms of TCM theory. 3b. State the importance of the review. |
| Objectives | 4 | Provide an explicit statement of questions being addressed with reference to participants, interventions, comparisons, out- comes, and study design (PICOS). | State whether the CHM intervention(s) targets a Western medicine-deﬁned disease, a TCM Pattern, or a Western medicine-deﬁned disease with a speciﬁc TCM Pattern. |
| **METHODS** | | | |
| Protocol and registration | 5 | Indicate if a review protocol exists, if and where it can be accessed (e.g., Web address), and, if available, provide registration information including registration number. |  |
| Eligibility criteria | 6 | Specify study characteristics (e.g., PICOS, length of follow-up) and report characteristics (e.g., years considered, language, publication status) used as criteria for eligibility, giving rationale. | 6a. As applicable, state whether participants with a speciﬁc TCM Pattern will be in-cluded, in terms of (1) diagnostic criteria,and (2) inclusion and exclusion criteria. All criteria utilized should be universally recognized, or reference(s) where detailed explanation(s) can be found should be given. 6b. Specify the detailed requirements of CHM intervention(s), considering (1) types, such as whether CHM formulas are ﬁxed, individualized, or patent proprietary; (2) composition, such as main herb(s) in a CHM formula; (3) dosage form, such as decoction, granules, powder; and (4) treatment duration. 6c. Specify the types of control group(s), if any, such as placebo control, active control, other treatment control or blank control. 6d. State whether TCM-related outcome(s) will be included, and if so, describe the change of degree and scope of symptoms and signs related to TCM Pattern differentiation.  6d. State whether TCM-related outcome(s) will be included, and if so, describe the change of degree and scope of symptoms and signs related to TCM Pattern differentiation. |
| Information sources | 7 | Describe all information sources (e.g., data- bases with dates of coverage, contact with study authors to identify additional studies) in the search and date last searched. |  |
| Search | 8 | State full electronic search strategy for at least one database, including any limits |  |
| Study selection | 9 | used, such that it could be repeated. State the process for selecting studies(i.e., screening, eligibility, included in systematic review, and, if applicable, included in the meta-analysis). |  |
| Data collection process | 10 | Describe method of data extraction from reports (e.g., piloted forms, indepen- dently, in duplicate) and any processes for obtaining and conﬁrming data from investigators. |  |
| Data items | 11 | List and deﬁne all variables for which data were sought (e.g., PICOS, funding sour- ces) and any assumptions and simpliﬁ- cations made. | 11a. State details of the participants with a speciﬁc TCM Pattern (if any), consid- ering (1) diagnostic criteria; and (2)baseline characteristics. 11b. State details of the CHM intervention(s), including (1) name, source, and dosage form; (2) name, source, processing method, and dosage of each medical substance, if applicable, names of the parts of the substances; (3) quality control information; (4) dosage,administration route and time; (5) information about the production method, authentication method, and safety as- sessment, if any; (6) for CHM formulas, the principles, rationale, and interpreta- tion of forming and/or modifying theformula; and (7) for patented proprietary CHM formulas, the name of the product and manufacturer. 11c. State details of any placebo of CHM used, considering (1) if/how it is physically identical and pharmacologically inert; (2) administration route, regimen, and dosage; (3) success of blinding, if any. 11d. State the TCM-related outcome (if any), considering (1) name and measuring methods; (2) measuring time points and length of follow-up, if applicable. |
| Risk of bias in individual studies | 12 | Describe methods used for assessing risk of bias of individual studies (including speciﬁcation of whether this was done at the study or outcome level), and how this information is to be used in any data synthesis |  |
| Summary measures | 13 | State the principal summary measures (e.g., risk ratio, difference in means). |  |
| Synthesis of results | 14 | Describe the methods of handling data and combining results of studies, if done, in- cluding measures of consistency (e.g., I2) for each meta-analysis. | When combining trial results from different studies, describe whether CHM intervention(s) matched the TCM Pattern(s) of participants, if applicable. |
| Risk of bias across studies | 15 | Specify any assessment of risk of bias that may affect the cumulative evidence (e.g., publication bias, selective reporting within studies). |  |
| Additional analyses | 16 | Describe methods of additional analyses (e.g., sensitivity or subgroup analyses, meta-regression), if done, indicating which were pre-speciﬁed. | Describe methods of subgroup analyses in terms of the CHM intervention(s) and participants, considering at least (1) the types, composi- tions, dosage, dosage form, and treatment duration of the CHM intervention(s); and (2)participants with different TCM Patterns, if any. |
| **RESULTS** | | | |
| Study selection | 17 | Give numbers of studies screened, assessed for eligibility, and included in the review, with reasons for exclusions at each stage, ideally with a ﬂow diagram. |  |
| Study characteristics | 18 | For each study, state characteristics for which data were extracted (e.g., study size, PICOS, follow-up period) and provide the citations.PICOS, follow-up period) and provide the citations. | 18a. State characteristics of participants witha specific TCM Pattern (if any), in cluding diagnostic criteria and baseline data. 18b. State characteristics of the CHM inter vention(s), including (1) name, source,and dosage form; (2) name, source,processing method, and dosage of eachmedical substance; (3) quality control information; (4) dosage, administration route and time; (5) information about the production method, authentication method, and safety assessment, if available; (6) for CHM formulas, how the formula has been modified, if ap plicable; and (7) for patent proprietary CHM formulas, the name of the product and manufacturer. 18c. State characteristics of the placebo of CHM (if any), including (1) whether physically identical and pharmacologi- cally inert; (2) administration route, regimen, and dosage; and (3) success of blinding. 18d. State ch aracteristics of the TCM-related outcome (if any), including (1) name and measuring methods; (2) measuring time points and length of follow-up. |
| Risk of bias within studies | 19 | Present data on risk of bias of each study and, if available, any outcome-level assess ment (see Item 12). |  |
| Results of individual studies | 20 | For all outcomes considered (benefits or harms), present, for each study: (a) simple summary data for each intervention group and (b) effect estimates and confidence intervals, ideally with a forest plot. |  |
| Synthesis of results | 21 | Present results of each meta-analysis done, including confidence intervals and measures of consistency. | Give results of each meta-analysis based on the consistency of PICOS, considering (1) participants with TCM Patterns, if any; (2) CHM intervention(s); (3) comparators (e.g. CHM placebo); and (4) TCM-related outcome(s), if any. |
| Risk of bias across studies | 22 | Present results of any assessment of risk of bias across studies (see Item 15). |  |
| Additional analysis | 23 | Give results of additional analyses, if done (e.g., sensitivity or subgroup analyses, meta-regression (see Item 16)). | Give results of subgroup analyses based on the different categories of CHM intervention(s) and participants with TCM Patterns (if any), if done. |
| **DISCUSSION** | | | |
| Summary of evidence | 24 | Summarize the main findings including the strength of evidence for each main out come; consider their relevance to key groups (e.g., health care providers, users, and policy makers). | Summarize how the CHM intervention(s) worked on different TCM Pattern(s) or Western medicine–defined disease(s) with specific TCM Pattern(s). Interpret the main findings in terms of TCM theory, if applicable. |
| Limitations | 25 | Discuss limitations at study and outcome level (e.g., risk of bias), and at review level (e.g., incomplete retrieval of iden tified research, reporting bias). |  |

Notes: CHM = Chinese herbal medicines; PRISMA = Preferred Reporting Items for Systematic Reviews and Meta-analyses; TCM = traditional Chinese medicine.

**Supplementary Material 7. Summary of Key TCM Formulations.**

| **TCM Formulation**  **(English/Pinyin)** | **Core Composition**  **(Botanical Name + Family + Pinyin Name)** | **Pharmacopeial Reference**  **(Chinese Pharmacopoeia 2020 Edition)** | **Dosage**  **(Per Day)** | **Dosage Form** | **Administration Details** | **Typical Course** | **Reporting Completeness in Original Study** | **Key Limitation for Reproducibility** |
| --- | --- | --- | --- | --- | --- | --- | --- | --- |
| Yinchenhao Decoction (Yīnchénhāo Tāng) | - *Artemisia capillaris* Thunb. (Asteraceae, Yīnchén) | Chinese Pharmacopoeia 2020 Edition, Vol.Ⅰ, p. 238 (Yīnchén), Vol.Ⅰ, p. 22 (Dàhuáng), Vol.Ⅰ, p. 196 (Zhīzǐ) | Artemisia capillaris: 12–30 g/day; Rheum palmatum: 5–12 g/day | Decoction | Oral (decoction), 1–2 times/day | 8 weeks–48 weeks | Partial (basic information reported; no processing/quality control) | No decoction parameters (temperature, duration, botanical drug-to-water ratio); limited reproducibility |
|  | - *Rheum palmatum* L. (Polygonaceae, Dàhuáng) |  |  |  |  |  |  |  |
|  | - *Gardenia jasminoides* J.Ellis (Rubiaceae, Zhīzǐ) |  |  |  |  |  |  |  |
| Yinchenhao Decoction Modified (Yīnchénhāo Tāng Jiājiǎn) | - *Artemisia capillaris* Thunb. (Asteraceae, Yīnchén) | Chinese Pharmacopoeia 2020 Edition, Vol.Ⅰ, p. 238 (Yīnchén), Vol.Ⅰ, p. 22 (Dàhuáng), Vol.Ⅰ, p. 196 (Zhīzǐ), Vol.Ⅰ, p. 76 (Dānsēn) | Artemisia capillaris: 12 g/day; Rheum palmatum: 9 g/day | Decoction | Oral (decoction), 1 time/day | 24 weeks | Partial (basic information reported; no processing/quality control) | No decoction parameters (temperature, duration, botanical drug-to-water ratio); limited reproducibility |
|  | - *Rheum palmatum* L. (Polygonaceae, Dàhuáng) |  |  |  |  |  |  |  |
|  | - *Gardenia jasminoides* J.Ellis (Rubiaceae, Zhīzǐ) |  |  |  |  |  |  |  |
|  | - *Salvia miltiorrhiza* Bunge (Lamiaceae, Dānsēn) |  |  |  |  |  |  |  |
| Xiezhuo Jiedu Decoction (Xièzhuó Jiědú Tāng) | - *Artemisia capillaris* Thunb. (Asteraceae, Yīnchén) | Chinese Pharmacopoeia 2020 Edition, Vol.Ⅰ, p. 238 (Yīnchén), Vol.Ⅰ, p. 22 (Dàhuáng), Vol.Ⅰ, p. 129 (Fúlíng), Vol.Ⅰ, p. 288 (Cháihú) | Artemisia capillaris: 20 g/day; Rheum palmatum: 9 g/day | Decoction | Oral (decoction), 1 time/day | 8 weeks | Partial (basic information reported; no processing/quality control) | No decoction parameters (temperature, duration, botanical drug-to-water ratio); limited reproducibility |
|  | - *Rheum palmatum* L. (Polygonaceae, Dàhuáng) |  |  |  |  |  |  |  |
|  | - *Poria cocos* (Schw.) Wolf (Polyporaceae, Fúlíng) |  |  |  |  |  |  |  |
|  | - *Bupleurum chinense* DC. (Apiaceae, Cháihú) |  |  |  |  |  |  |  |
| Tiaozhi Huoxue Jiangtang Decoction (Tiáozhī Huóxuè Jiàngtáng Tāng) | - *Astragalus membranaceus* (Fisch.) Bunge (Fabaceae, Huángqí) | Chinese Pharmacopoeia 2020 Edition, Vol.Ⅰ, p. 76 (Dānsēn), Vol.Ⅰ, p. 28 (Huángqí), Vol.Ⅰ, p. 117 (Cāngzhú), Vol.Ⅰ, p. 74 (Xuánshēn) | 15–20 g/day (decoction) | Decoction | Oral (decoction), 2 times/day | 3 months | Partial (basic information reported; no processing/quality control) | No decoction parameters (temperature, duration, botanical drug-to-water ratio); limited reproducibility |
|  | - *Atractylodes chinensis* (DC.) Koidz. (Asteraceae, Cāngzhú) |  |  |  |  |  |  |  |
|  | - *Scrophularia ningpoensis* Hemsl. (Scrophulariaceae, Xuánshēn) |  |  |  |  |  |  |  |
|  | - *Salvia miltiorrhiza* Bunge (Lamiaceae, Dānsēn) |  |  |  |  |  |  |  |
| Liuwei Dihuang Pill (Liùwèi Dìhuáng Wán) | - *Rehmannia glutinosa* (Gaertn.) DC. (Scrophulariaceae, Shúdì) | Chinese Pharmacopoeia 2020 Edition, Vol.Ⅰ, p. 106 (Shúdì), Vol.Ⅰ, p. 297 (Shānyào), Vol.Ⅰ, p. 177 (Shānzhūyú), Vol.Ⅰ, p. 127 (Zéxiè) | 8 pills/day | Pill | Oral (pills), 3 times/day | 3 months | Partial (basic information reported; no processing/quality control) | No processing details or quality control; limited reproducibility |
|  | - *Dioscorea opposita* Thunb. (Dioscoreaceae, Shānyào) |  |  |  |  |  |  |  |
|  | - *Cornus officinalis* Sieb. et Zucc. (Cornaceae, Shānzhūyú) |  |  |  |  |  |  |  |
|  | - *Alisma orientale* (Sam.) Juz. (Alismataceae, Zéxiè) |  |  |  |  |  |  |  |
| Huazhuo Granule (Huàzhuó Kēlì) | - *Coptis chinensis* Franch. (Ranunculaceae, Huánglián) | Chinese Pharmacopoeia 2020 Edition, Vol.Ⅰ, p. 76 (Dānsēn), Vol.Ⅰ, p. 124 (Huánglián), Vol.Ⅰ, p. 208 (Huángbǎi), Vol.Ⅰ, p. 184 (Shānzhā) | 1 package/day (granule) | Granule | Oral (granules), 3 times/day | 6 months | Partial (basic information reported; no processing/quality control) | No processing details or quality control; limited reproducibility |
|  | - *Phellodendron amurense* Rupr. (Rutaceae, Huángbǎi) |  |  |  |  |  |  |  |
|  | - *Crataegus pinnatifida* Bunge (Rosaceae, Shānzhā) |  |  |  |  |  |  |  |
|  | - *Salvia miltiorrhiza* Bunge (Lamiaceae, Dānsēn) |  |  |  |  |  |  |  |
| Huazhi Rougan Granule (Huàzhì Róugān Kēlì) | - *Artemisia capillaris* Thunb. (Asteraceae, Yīnchén) | Chinese Pharmacopoeia 2020 Edition, Vol.Ⅰ, p. 238 (Yīnchén), Vol.Ⅰ, p. 22 (Dàhuáng), Vol.Ⅰ, p. 127 (Zéxiè), Vol.Ⅰ, p. 186 (Juémíngzǐ) | 1 bag/day (granule) | Granule | Oral (granules), 3 times/day | 3 months | Partial (basic information reported; no processing/quality control) | No processing details or quality control; limited reproducibility |
|  | - *Cassia obtusifolia* L. (Fabaceae, Juémíngzǐ) |  |  |  |  |  |  |  |
|  | - *Rheum palmatum* L. (Polygonaceae, Dàhuáng) |  |  |  |  |  |  |  |
|  | - *Alisma orientale* (Sam.) Juz. (Alismataceae, Zéxiè) |  |  |  |  |  |  |  |
| Baogan Jiangzhi Decoction (Bǎogān Jiàngzhī Tāng) | - *Astragalus membranaceus* (Fisch.) Bunge (Fabaceae, Huángqí) | Chinese Pharmacopoeia 2020 Edition, Vol.Ⅰ, p. 76 (Dānsēn), Vol.Ⅰ, p. 28 (Huángqí), Vol.Ⅰ, p. 184 (Shānzhā) | 15-30 g/day (decoction) | Decoction | Oral (decoction), 2 times/day | 3 months | Partial (basic information reported; no processing/quality control) | No decoction parameters (temperature, duration, botanical drug-to-water ratio); limited reproducibility |
|  | - *Salvia miltiorrhiza* Bunge (Lamiaceae, Dānsēn) |  |  |  |  |  |  |  |
|  | - *Crataegus pinnatifida* Bunge (Rosaceae, Shānzhā) |  |  |  |  |  |  |  |
|  | - *Polygonum cuspidatum* Sieb. & Zucc. (Polygonaceae, Hǔzhàng) |  |  |  |  |  |  |  |
| Danshen (Dānsēn) | - *Salvia miltiorrhiza* Bunge (Lamiaceae, Dānsēn) | Chinese Pharmacopoeia 2020 Edition, Vol.Ⅰ, p. 76 (Dānsēn) | 10–15 g/day (decoction); 0.5–1 g/day (extract) | Decoction, Extract | Oral, 1–2 times/day | 14-90 days | Partial (basic information reported; no processing/quality control) | No marker component quantification (danshensu, tanshinone IIA); unreported extraction method |
| Wendan Decoction (Wēndǎn Tāng) | - *Pinellia ternata* (Thunb.) Breit. (Araceae, Bànxià) | Chinese Pharmacopoeia 2020 Edition, Vol.Ⅰ, p. 149 (Bànxià), p. 312 (Zhúrú), p. 130 (Zhǐshí), p. 132 (Chénpí), p. 129 (Fúlíng), p. 72 (Gāncǎo) | 10–20 g/botanical drug (total decoction) | Decoction | Oral, 2 times/day | 2-3 months | Partial (basic information reported; no processing/quality control) | Unclear individual botanical drug dosage; no quality control for Bànxià (alkaloid content limit) |
|  | - *Bambusa tuldoides* Munro (Poaceae, Zhúrú) |  |  |  |  |  |  |  |
|  | - *Citrus aurantium* L. (Rutaceae, Zhǐshí) |  |  |  |  |  |  |  |
|  | - *Citrus reticulata* Blanco (Rutaceae, Chénpí) |  |  |  |  |  |  |  |
|  | - *Poria cocos* (Schw.) Wolf (Polyporaceae, Fúlíng) |  |  |  |  |  |  |  |
|  | - *Glycyrrhiza uralensis* Fisch. (Fabaceae, Gāncǎo) |  |  |  |  |  |  |  |
| Xiaoyao Powder (Xiāoyáo Sǎn) | - *Bupleurum chinense* DC. (Apiaceae, Cháihú) | Chinese Pharmacopoeia 2020 Edition, Vol.Ⅰ, p. 288 (Cháihú), p. 108 (Dāngguī), p. 105 (Báisháo), p. 118 (Báizhú), p. 129 (Fúlíng), p. 72 (Gāncǎo), p. 333 (Bòhe), p. 341 (Shēngjiāng) | 9 g/botanical drug (decoction); 6 g/pill (honeyed pill, 3 pills/day) | Decoction, Honeyed pill | Oral, 2–3 times/day | 8 weeks-6 months | Partial (basic information reported; no processing/quality control) | Lack of processing parameters (e.g., stir-frying of Báizhú); no HPLC fingerprinting for marker components |
|  | - *Angelica sinensis* (Oliv.) Diels (Apiaceae, Dāngguī) |  |  |  |  |  |  |  |
|  | - *Paeonia lactiflora* Pall. (Paeoniaceae, Báisháo) |  |  |  |  |  |  |  |
|  | - *Atractylodes macrocephala* Koidz. (Asteraceae, Báizhú) |  |  |  |  |  |  |  |
|  | - *Poria cocos* (Schw.) Wolf (Polyporaceae, Fúlíng) |  |  |  |  |  |  |  |
|  | - *Glycyrrhiza uralensis* Fisch. (Fabaceae, Gāncǎo) |  |  |  |  |  |  |  |
|  | - *Mentha haplocalyx* Briq. (Lamiaceae, Bòhe) |  |  |  |  |  |  |  |
|  | - *Zingiber officinale* Rosc. (Zingiberaceae, Shēngjiāng) |  |  |  |  |  |  |  |
| Erchen Decoction (Èrchén Tāng) | - *Pinellia ternata* (Thunb.) Breit. (Araceae, Bànxià) | Chinese Pharmacopoeia 2020 Edition, Vol.Ⅰ, p. 129 (Fúlíng), Vol.Ⅰ, p. 72 (Gāncǎo), Vol.Ⅰ, p. 132 (Chénpí), Vol.Ⅰ, p. 149 (Bànxià) | 6-15 g/day per botanical drug (decoction) | Decoction | Oral (decoction), 2 times/day | 1-2 months | Partial (basic information reported; no processing/quality control) | No decoction parameters (temperature, duration, botanical drug-to-water ratio); limited reproducibility |
|  | - *Citrus reticulata* Blanco (Rutaceae, Chénpí) |  |  |  |  |  |  |  |
|  | - *Poria cocos* (Schw.) Wolf (Polyporaceae, Fúlíng) |  |  |  |  |  |  |  |
|  | - *Glycyrrhiza uralensis* Fisch. (Fabaceae, Gāncǎo) |  |  |  |  |  |  |  |
| Lingguizhugan Decoction (Língguì Zhúgān Tāng) | - *Poria cocos* (Schw.) Wolf (Polyporaceae, Fúlíng) | Chinese Pharmacopoeia 2020 Edition, Vol.Ⅰ, p. 129 (Fúlíng), Vol.Ⅰ, p. 118 (Báizhú), Vol.Ⅰ, p. 72 (Gāncǎo), Vol.Ⅰ, p. 165 (Guìzhī) | 10-15 g/day per botanical drug (decoction) | Decoction | Oral (decoction), 1 time/day | 12 weeks | Partial (basic information reported; no processing/quality control) | No decoction parameters (temperature, duration, botanical drug-to-water ratio); limited reproducibility |
|  | - *Cinnamomum cassia* Presl (Lauraceae, Guìzhī) |  |  |  |  |  |  |  |

Notes:

1. All botanical names follow the Flora of China and Chinese Pharmacopoeia (2020 Edition) for taxonomic consistency.
2. "Pharmacopeial Reference" indicates the specific volume and page number in the Chinese Pharmacopoeia (2020 Edition, Volume Ⅰ) where quality standards for each herbal ingredient are defined.

3. "Reporting Completeness" categories:
 - Complete: All key information (composition, dosage, processing, quality control) reported
 - Partial: Core information (composition + dosage) reported, non-core details missing
 - Incomplete: Only basic information reported, critical details missing
4. "Key Limitation for Reproducibility" summarizes unresolved gaps that prevent consistent replication of the TCM formulation.
